# Effect of HPV vaccination and cervical cancer screening in England by ethnicity: a modelling study

**DOI:** 10.1016/S2468-2667(17)30238-4

**Published:** 2017-12-19

**Authors:** Helen C Johnson, Erin I Lafferty, Rosalind M Eggo, Karly Louie, Kate Soldan, Jo Waller, W John Edmunds

**Affiliations:** a Centre for the Mathematical Modelling of Infectious Diseases, London School of Hygiene & Tropical Medicine, London, UK; b Wolfson Institute of Preventive Medicine, Queen Mary University of London, London, UK; c National Centre for Infectious Disease Surveillance and Control (CIDSC), London, UK; d Health Behaviour Research Centre, London, UK

## Abstract

**Background:**

Health equality is increasingly being considered alongside overall health gain when assessing public health interventions. However, the trade-off between the direct effects of vaccination and herd immunity could lead to unintuitive consequences for the distribution of disease burden within a population. We used a transmission dynamic model of human papillomavirus (HPV) to investigate the effect of ethnic disparities in vaccine and cervical screening uptake on inequality in disease incidence in England.

**Methods:**

We developed an individual-based model of HPV transmission and disease, parameterising it with the latest data for sexual behaviour (from National Survey of Sexual Attitudes and Lifestyles [Natsal-3]) and vaccine and screening uptake by ethnicity (from Public Health England [PHE]) and fitting it to data for HPV prevalence (from ARTISTIC, PHE, Natsal-3) and HPV-related disease incidence (from National Cancer Registry [ONS]). The outcome of interest was the age-adjusted incidence of HPV-related cancer (both cervical and non-cervical) in all women in England in view of differences and changes in vaccination and screening uptake by ethnicity in England, over time. We also studied three potential public health interventions aimed at reducing inequality in HPV-related disease incidence: increasing uptake in black and Asian females to match that in whites for vaccination; cervical screening in women who turn 25 in 2018 or later; and cervical screening in all ages.

**Findings:**

In the pre-vaccination era, before 2008, women from ethnic minorities in England reported a disproportionate share of cervical disease. Our model suggests that Asian women were 1·7 times (95% credibility interval [CI] 1·1–2·7) more likely to be diagnosed with cervical cancer than white women (22·8 *vs* 13·4 cases per 100 000 women). Because HPV vaccination uptake is lower in ethnic minorities, we predict an initial widening of this gap, with cervical cancer incidence in Asian women up to 2·5 times higher (95% CI 1·3–4·8) than in white women 20 years after vaccine introduction (corresponding to an additional 10·8 [95% CI 10·1–11·5] cases every year). In time, we predict that herd immunity benefits will diffuse from the larger white sub-population and the disparity will narrow. Increased cervical screening uptake in vaccinated women from ethnic minorities would lead to rapid improvement in equality with parity in incidence after 20 years of HPV vaccination.

**Interpretation:**

Our study suggests that the introduction of HPV vaccination in England will initially widen a pre-existing disparity in the incidence of HPV-related cancer by ethnicity, partly due to herd immunity disproportionately benefiting subgroups with high vaccination rates. Although in time this induced disparity will narrow, increasing cervical screening uptake in girls from ethnic minorities should be encouraged to eliminate the inequality in cervical cancer incidence in the medium term. We recommend that dynamic effects should be considered when estimating the effect of public health programmes on equality.

**Funding:**

Cancer Research UK.

## Introduction

HPV infection is implicated in more than 99% of cervical cancer cases^1^ with roughly 70% caused by types HPV-16 and HPV-18.^2^ Since 2006, two highly effective prophylactic vaccines against HPV-16 and HPV-18 have been available worldwide^3^ and cervical screening remains an effective secondary prevention strategy. HPV infection has also been implicated in some anal, genital, and head and neck cancers.^3^

In 2008, the UK initiated school-based vaccination of 12–13 year-old-girls, with more than 86% of girls receiving a full course of vaccination between 2012 and 2014.^4^ However, findings of demographic studies have consistently reported substantially higher levels of vaccination uptake in white girls than in those from minority ethnic backgrounds.^5^, ^6^ An equivalent disparity is reported in cervical screening attendance: white women are more than twice as likely to have attended screening as women of another ethnic origin.^7^, ^8^ Indeed, a positive association has been recorded between uptake of vaccination and of cervical cancer screening in both empirical^9^, ^10^ and survey-based^5^, ^9^ studies in the UK. Herd immunity, the indirect protection afforded to unvaccinated individuals through reduced pathogen circulation, might mitigate this effect. However, findings of a recent study have shown clear patterns of so-called like-with-like sexual mixing (ie, choosing a sexual partner like oneself) by ethnicity in Britain,^11^ raising concerns that the herd immunity benefit might be concentrated in ethnic subgroups with high HPV vaccination rates.

**Research in context**

**Evidence before this study**

We searched PubMed on Sept 16, 2017, with no language or date restrictions for articles seeking to quantify the effects of HPV vaccination on health equality with the following search terms: “HPV”, “model OR model(l)ing”, and “(in)equity OR (in)equality”. In the past 5 years, modelling studies have suggested that HPV vaccination might cause an increase in health inequality but we found none that used behavioural data to assess the interplay between sexual mixing and health-care-seeking behaviour.

**Added value of this study**

We modelled the interdependency between sexual mixing and vaccination and screening uptake, using data stratified by ethnicity. Data suggest that people are more likely to choose sexual partners from within a group with shared patterns of health-seeking behaviour. We believe this to be the first study to base the evaluation of the dynamic effects of such like-with-like mixing on behavioural data. Our modelling study suggests that the introduction of the HPV vaccine in England will initially widen a pre-existing disparity in the incidence of HPV-related cancer by ethnicity. We predict that over time herd immunity effects will redress this gap at a rate dependent on the degree to which sexual partnerships are formed between groups. We show that increasing cervical screening uptake among vaccinated girls from ethnic minorities could eliminate the inequality in cervical cancer incidence within 30 years.

**Implications of all the available evidence**

The case study of HPV, cancer, and ethnicity highlights important factors in assessing the effect on health equality of new and existing public health programmes. Our modelling analysis shows that the interplay of infection dynamics with disparities in vaccination and screening uptake could lead to increased health inequality. We recommend that dynamic modelling studies, parameterised with behavioural data, be used to better understand these trade-offs.

To understand the complicated nature of infection dynamics and disease progression, and the multiple behavioural and demographic factors such as those associated with HPV and cervical cancer, mathematical models are often used.^12^, ^13^, ^14^ Recent studies have considered the hypothetical effect of HPV vaccination on health inequality^14^, ^15^, ^16^, ^17^ and others have questioned the implications of difference in vaccination uptake by ethnicity;^18^, ^19^ however, none to our knowledge have made a data-based evaluation of the implicit dependency of sexual mixing and health-care-seeking behaviour.

We developed an individual-based model of HPV infection, cervical disease, and HPV-attributable non-cervical cancer in England, parameterising it with the latest ethnicity-stratified data on sexual behaviour and vaccination and screening uptake. Here we describe the development, parameterisation, and fitting of the model to data on HPV prevalence and incidence of cervical disease and non-cervical cancers; make predictions of the consequences of HPV vaccination on health equality over time; and estimate the effect of three potential public health interventions aiming to reduce inequality in HPV-related disease incidence.

## Methods

### Study design

We developed a generic individual-based sexually transmitted infection model^20^ to simulate heterosexual partnership formation on the basis of sexual activity and ethnicity, multi-type HPV transmission and disease progression, cervical screening, and HPV vaccination. The outcome of interest was the incidence of age-adjusted HPV-related cancer incidence (both cervical and non-cervical, using the European Standard Population) in view of differences and changes in vaccination and screening uptake by ethnicity. The model was coded in C++ and implemented on the Amazon Web Services EC2 service as a virtual cluster on a StarCluster platform (version 0.95.6). Statistical analysis was done with Stata (version 12) and R (version 3.1.1).

### Parameterisation

We parameterised the model with demographic data from the Office for National Statistics (ONS)^21^, ^22^, ^23^ and behavioural data from the latest National Survey of Sexual Attitudes and Lifestyles (Natsal-3).^24^, ^25^, ^26^, ^27^ We fitted the model to age-specific HPV prevalence data obtained from: Natsal-3; Public Health England (PHE) surveillance of HPV in young women attending chlamydia screening;^28^ a PHE study of women attending cervical screening;^29^ and a randomised trial of cytological testing in women attending routine cervical screening (ARTISTIC).^30^ To account for biases in the underlying subpopulations, we used a Bayesian evidence synthesis approach to estimate the true type-specific prevalence of HPV in the population (appendix p 20). We obtained data for cervical disease prevalence by age and type from the ARTISTIC study. Data for age-dependent incidence of carcinoma in situ of cervix uteri and of HPV-related cancers was obtained from the National Cancer Registry (ONS). Attribution of carcinoma in situ of cervix uteri and cancer by HPV type was made according to the proportion of histological samples testing positive for each type in the case of cervical cancer (appendix p 14); data from PHE) and according to the scientific literature^31^, ^32^, ^33^ for non-cervical cancer (appendix p 15).

### Model structure

The model population (n=100 000) was split by sex (50:50) and ethnicity (white [88·8%], black [3·4%], and Asian [7·8%]).^34^ Each individual belonged to one of nine age groups and one of two sexual activity groups (because HPV is highly prevalent, we assumed its transmission was not driven by smaller core groups). For each age group, the high sexual activity group was parameterised with the characteristics of the 15% of NATSAL-3 respondents reporting the highest number of partners in the preceding year. We ran 100 simulations of the model for each scenario, giving an effective population size of 10 000 000 yet allowing for computational parallelisation.

For partnership formation, we modelled three types of non-concurrent heterosexual partnership: casual, steady, and married or cohabiting. Partnership formation followed a female demand model.^20^ The age and sexual activity group of the female partner determined both the delay before a new partnership was formed and the partnership type. Partnership type determined partner choice (by age and ethnicity) and partnership duration.

We modelled 13 high-risk HPV types (−16, −11, −31, −33, −35, −39, −45, −51, −52, −56, −58, −59, and −68) and two low-risk type (−06, −11). High-risk types could potentially progress to either squamous cell carcinoma or adenocarcinoma via three stages of intraepithelial neoplasia (CIN1-3 and CGIN1-3, respectively) and a stage of carcinoma in situ. We established the branching fraction of each type towards squamous cell carcinoma versus adenocarcinoma by the proportion of cancer type attributed to each HPV type^29^ (appendix p 14). We assumed that type-specific naturally-acquired immunity was lifelong. Additionally, we modelled, with competing risks, the progression of high-risk HPV infection to non-cervical (anal, oropharyngeal, laryngeal, penile, and vulval or vaginal) cancers. We estimated type-specific transmission probabilities and progression and clearance rates as part of the model-fitting process. The appendix provides flowcharts of the natural history of infection and disease progression (appendix pp 12, 13) and posterior values of estimated parameters (appendix pp 34–38).

**Figure 1:**
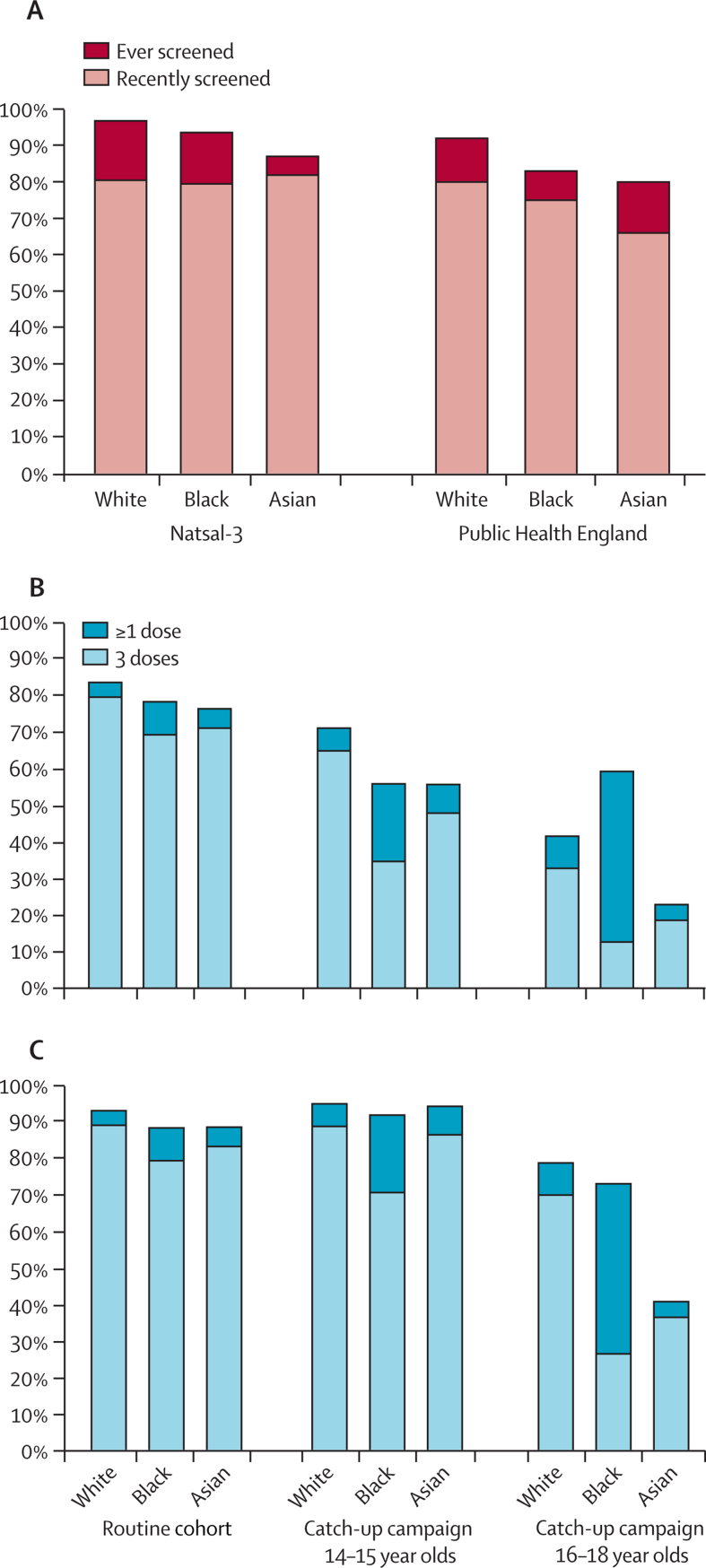
Uptake of cervical screening and HPV vaccination by ethnicity Cervical screening attendance by ethnicity as estimated by Natsal-3 and Public Health England (A). We termed “recent” screening to be within the past 5 or past 10 years for those younger or older than 50 years, respectively, thereby including women who were overdue a routine appointment. Uptake of HPV vaccination for the routine cohort and two catch-up cohorts, by ethnic group based on data obtained from the clinical practice research datalink (B) and primary care trusts (C). Bar height shows proportion of women receiving at least one dose; light blue shows the subset fully vaccinated.

We implemented the National Health Service (NHS) protocol for England (appendix p 17)^35^ in which women are invited for cervical screening every 3 years between the ages of 25 and 49 years and every 5 years between the ages of 50 and 64 years. We specified the proportion of women who were full, partial, or non-attendees of cervical screening by ethnicity according to Natsal-3 data. PHE data suggested lower uptake across all ethnic groups than Natsal-3 and a larger discrepancy between white and black and Asian women (figure 1A). Stage-dependent sensitivity and specificity estimates for cervical screening were obtained from a UK-specific literature review (appendix p 16).^36^, ^37^ We assumed the odds ratio for cervical screening attendance in vaccinees was 1·6, in line with a school-based study of screening intention^5^ and early findings from Wales^9^ and Scotland,^10^ in which women were invited for screening from the age of 20 years.

We implemented the UK vaccination programme, vaccinating a routine cohort (aged 12–13 years) once a year from 2008 and two catch-up cohorts (aged 14–15 years and 16–18 years) between 2009 and 2011 with three doses of bivalent Cervarix (administered at 0, 1, and 6 months). From 2012, the vaccine changed to the quadrivalent Gardasil (administered at 0, 2, and 6 months), reducing to two doses in 2014. Data for HPV vaccination uptake by ethnicity were obtained from eight child health databases stored at the clinical practice research datalink and the primary care trusts (figure 1B, C). In the routine cohort, more than 80% of girls had initiated HPV vaccination and more than 70% of girls had completed HPV vaccination. However, in the 16–18-year-old catch-up cohort, coverage was much lower with fewer than 60% of girls initiating HPV vaccination. In all cohorts, white girls were more likely to have initiated or completed vaccination than girls of black or Asian ethnicity. We used the primary care trust data to parameterise the model because the clinical practice research datalink data can be less representative because of delays in database updating.

We considered the efficacy of two or three doses of either bivalent or quadrivalent vaccine against persistent HPV-16 and HPV-18 infection to be 95% with reduced protection offered by one dose (90% efficacy).^38^, ^39^ We assumed protection to last precisely 20 years (sensitivity analysis of lifelong protection is reported in the appendix [p 21]). We also modelled cross-protection of both vaccines against non-vaccine types to range between 18% and 79%, according to the levels estimated by Wheeler and colleagues^40^ and Malagon and colleagues^41^ (appendix p 18), assuming the duration of cross-protection to be shorter (10 years).

For model fitting we used a sequential Monte Carlo approach to estimate transmission probability and rates of disease progression and clearance for each HPV type, assuming that transmission probability and rate of clearance of initial infection to be the same for men as for women. Rates of progression to non-cervical cancers were allowed to differ between men and women. For each scenario, we ran the model for the 100 best-fitting parameter sets.

For intervention analyses we modelled alternative scenarios to assess interventions that might reduce inequality. We considered intervening 10 years after vaccine introduction (2018), increasing black and Asian uptake to match white uptake for: vaccination; cervical screening for women who turn 25 in 2018 or later; and cervical screening for all ages.

### Role of the funding source

The funder of the study had no role in study design, data collection, data analysis, data interpretation, or writing of the report. The corresponding author had full access to all the data in the study and had final responsibility for the decision to submit for publication.

## Results

**Figure 2:**
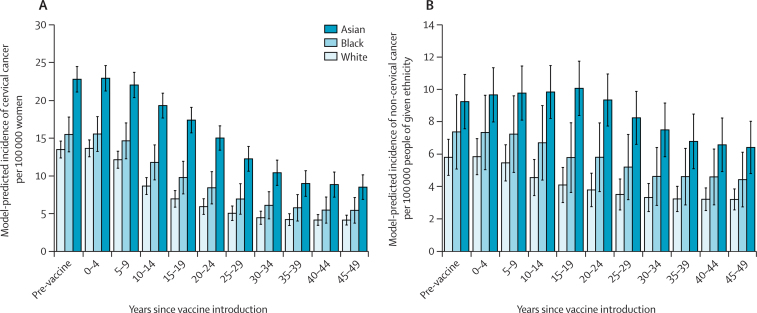
Model-predicted incidence of cancer by ethnicity, over time (A) Annual incidence of cervical cancer per 100 000 white, black, and Asian women. (B) Annual incidence of HPV-attributable non-cervical cancer per 100 000 members of the white, black, and Asian populations. Bar height shows the mean model output for 100 simulations, the error bars the 95% credibility interval.

Our model predicts that the age-standardised incidence of cervical cancer will initially remain stable for roughly 10 years before falling sharply, with a time lag of 20 years to achieve an overall 50% reduction in the age-standardised incidence of cervical cancer after 30 years. We estimated a long-term equilibrium incidence of roughly 4·4 cases of cervical cancer per 100 000 women per year (figure 2A) These residual cases were caused by non-vaccine HPV types. Incidence of HPV-attributable non-cervical cancer (including anal, oropharyngeal, laryngeal, vulval, vaginal, and penile cancer) decreased more slowly because of slower progression to disease, but we estimate that 50 years after the introduction of the HPV vaccine to girls, the incidence of such cancer will fall to 55% of its original level in both men and women (figure 2B).

**Figure 3:**
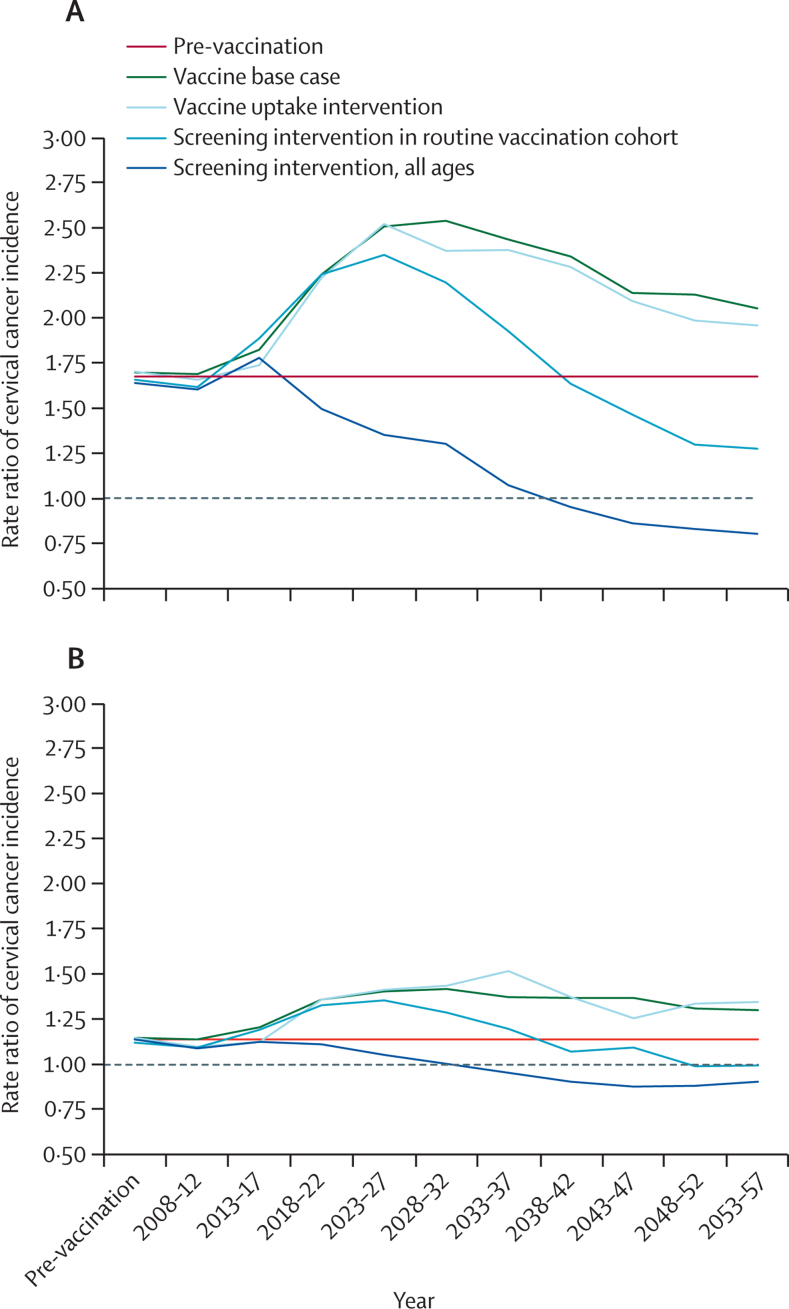
Rate ratios of model-predicted cervical cancer incidence over time comparing (A) Asian with white women and (B) black with white women Figure shows the effect of HPV vaccination introduction as our base case scenario, together with three potential interventions for addressing the resultant increase in inequality. We estimated the effect of increasing ethnic minority uptake of HPV vaccination, cervical screening in the routine vaccination cohort, and cervical screening of all ages to match the levels observed in the white sub-population. Interventions were made in 2018 (10 years after the introduction of the HPV vaccine).

**Figure 4:**
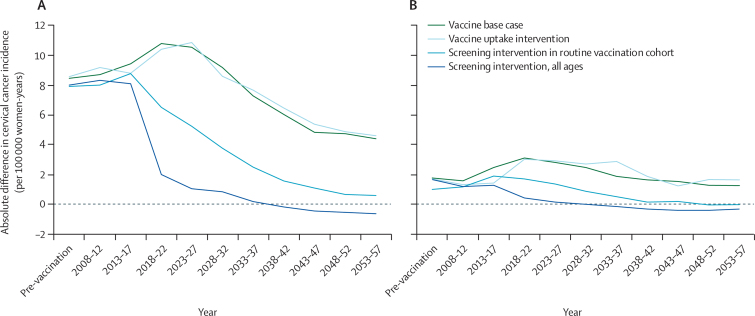
Absolute difference in model-predicted cervical cancer incidence per 100 000 women over time comparing (A) Asian with white women and (B) black with white women

In the pre-vaccination era, our model suggests that cervical cancer incidence was 1·7 times (95% credibility interval [CI] 1·1–2·7) higher in the Asian than the white population. In absolute terms, this corresponds to an additional 8·5 (7·9–9·1) cases of cervical cancer per 100 000 women-years. After HPV vaccine introduction in 2008, we predict the inequality in cancer incidence between these two groups will become greater (Figure 3, Figure 4). By 2027, 20 years after vaccine introduction, the incidence rate of cervical cancer will be 2·5 times higher among Asian than white women (95% CI 1·3–4·8), corresponding to an additional 10·8 (95% CI 10·1–11·5) cases annually. However, more than 20 years after vaccine introduction, we predict the rate ratio of cervical cancer incidence will reduce. 50 years after vaccine introduction, we estimate incidence in Asian women will be only 1·9 times as high as in white women (95% CI 0·97–3·6). Our analysis suggests that cervical cancer incidence is higher in the black population than amongst their white counterparts, with a similar pattern of increasing disparity in the first 20–30 years, followed by a decrease (figure 3B). However, these results were not statistically significant in our modelled population at the 95% credibility level.

Findings of our intervention analysis showed that interventions to reduce inequality in cervical disease should focus on aiming for parity in either HPV vaccination or cervical screening uptake. We predict a 2018 campaign raising vaccination uptake in black and Asian girls to levels of their white peers would have little effect on distribution of incident cervical cancer over the following 40 years (Figure 3, Figure 4). However, an intervention achieving equivalent rates of cervical screening attendance between black and Asian women from the routine vaccination cohort and their white counterparts will lead to a more rapid reduction in the inequality, reaching pre-vaccination levels 18 and 22 years after the intervention for black and Asian women, respectively. Indeed, 30 years after vaccine introduction, the distribution of incident cervical cancer would be more equitable than in the pre-vaccination era. A 2018 intervention resulting in cervical screening uptake among black and Asian women of all ages matching that of white women would cause a rapid correction in inequality and after 20 years the ethnic inequality in cervical disease would be overcome, overcoming the ethnic inequality completely after 12 and 20 years, respectively, for black and Asian women.

## Discussion

Data from our modelling analysis suggest that due to differences in sexual behaviour (including a tendency to choose partners from a similar background as oneself) and uptake of cervical screening, ethnic minorities in the UK are disproportionately affected by HPV-related disease, including cancer. Because uptake of HPV vaccination has been lower in black and Asian girls than in white girls, we predict that there will be increased inequality in HPV-attributable cancer incidence between ethnic minorities over the medium term (in both absolute and relative terms).

Initially, the consequences of the HPV vaccination programme are dominated by the direct protection of vaccinated girls themselves. Because vaccination uptake in both routine and catch-up cohorts was higher among white girls, more cancer cases were averted in this sub-population per person than in black and Asian groups. However, over time, indirect effects will also affect cancer incidence. Our modelling analysis suggests that between 20 and 50 years after vaccine introduction, the incidence rate ratio will fall once more until cervical cancer incidence in Asian girls is around 1·9 times that in white girls. Here we see the effect of the herd immunity effect: as the prevalence of HPV circulating in the overall population is reduced, the number of infections in unvaccinated girls of all ethnicities is also reduced and consequently so is HPV-related disease incidence. In the long term, the protective effect of herd immunity will overcome the discrepancy in vaccination uptake.

However, our analysis also showed that even if HPV vaccination uptake were increased among black and Asian girls to levels of their white peers, cohort effects and slow progression to cancer would cause the induced inequality to persist for at least 40 years following the intervention. To reduce the resultant inequality more rapidly, it would be necessary to address the difference in cervical screening uptake, thus protecting the unvaccinated. Although it might be easier to encourage vaccinees to attend cervical screening, this would lead to an even greater concentration of unprotected women in the subgroup which is neither vaccinated not screened. It might be preferable to actively seek out unvaccinated women to attend cervical screening.

This work builds on a number of other studies that have raised the question of how differences in uptake of vaccination and cervical screening will affect equality in HPV-related disease outcomes. Initial explorations considered hypothetical cases of increasing assortative mixing (assortativity) in partner choice,^14^, ^15^, ^16^, ^17^ others used ethnicity-specific data on vaccination and screening uptake but did not consider sexual mixing.^18^, ^19^ The strength of this study lies in the data-grounded modelling approach: by using an individual-based transmission dynamic model, we have been able to incorporate heterogeneity in both sexual and health-protective behaviour by ethnicity, including detailed data on partner choice by ethnicity and age, and to track an individual's life history of infection, vaccination, and disease. Uptake of both HPV vaccination and cervical screening has been associated with social deprivation in the UK. However, we chose to focus on ethnic heterogeneity due to the availability of sexual behaviour (in particular, mixing) data by ethnicity.

Our study also has limitations. In line with other HPV modelling studies, we make the simplifying assumption that neither sexual behaviour nor health-protective behaviour varies demographically over time. In fact, the three rounds of Natsal have documented significant time trends, for example showing increasingly younger sexual début.^25^ It would be pertinent to this study to better understand relative rates of sexual behaviour change between ethnic groups and also the association between changes in sexual behaviour and screening and vaccination uptake. If behaviour becomes more similar between ethnic groups, or if assortativity in partner choice decreases, inequality of disease incidence might naturally decrease.

Second, we consider only three ethnic groups, belying more complex behavioural heterogeneity. For example, 88% of people were classified as white, including a substantial (and growing) proportion of non-UK born individuals about whose vaccination and screening behaviour we know relatively little. Furthermore, we did not model migration. The effect of migration on health equality will depend on the relative rates of screening and vaccination uptake compared with the native population and on sexual mixing choices. If migrant populations choose partners from within their own communities or have a relatively low uptake of public health interventions, inequality might be increased further. Furthermore, we only modelled two sexual activity groups because we assumed that the high prevalence of HPV implies its dynamics are not driven by a highly active core group.

We made some limiting assumptions about the natural history of HPV. First, we assumed that the infection with one HPV type offers lifetime protection against reinfection with the same type. In forthcoming work, we will reassess this assumption in line with a recent review.^42^ Second, we assumed that higher-grade lesions (CIN2+) do not spontaneously regress and can be diagnosed with certainty. This assumption belies the clinical challenges of cytological categorisation but, because we fit the model to data on cervical cancer and carcinoma in situ, should not detract from our findings about changes in inequality of cancer incidence.

Discussion about the cost-effectiveness of including boys in the UK HPV vaccination programme is ongoing. Although achieving parity in HPV vaccination uptake between girls of different ethnicities might not address the increased inequality in cervical cancer incidence, increasing overall vaccine coverage by vaccinating boys could heighten the herd effect sufficiently to hasten its reduction. The appendix provides our preliminary yet inconclusive consideration of this issue (appendix p 22).

Future work should consider the marginal effect on ethnic inequality associated with the introduction of HPV testing as a primary test of HPV infection in view of indications of early studies that psychological burden of infection is associated with social and cultural norms.^43^

In conclusion, the case study of HPV, cancer, and ethnicity highlights important factors in assessing the effect on health equality of new and existing public health programmes. Public health interventions should aim to provide not only a population-level health gain but also to maintain, preferably improve, the level of equality, whether on the basis of socioeconomic status, ethnicity, or age. Our modelling analysis shows that the interplay of infection dynamics with disparities in health-protective behaviour can be unintuitive. Direct vaccination benefits will mirror differences in vaccination uptake between groups. Additional herd immunity benefits will be dependent on the degree to which subgroups mix and the extent to which their behaviour differs. We recommend dynamic effects should be considered when estimating the effect of vaccination and screening programmes on health inequality and in planning its minimisation
