# Supplementary material for: Effect of HPV vaccination and cervical cancer screening in England by ethnicity: a modelling study
Source: Lancet Public Health. 2017 Dec 19;3(1):e44–51. doi: 10.1016/S2468-2667(17)30238-4 (PMC5765530; doi:10.1016/S2468-2667(17)30238-4)
Supplement: Supplementary appendix [file mmc1.pdf]

THE LANCET
Public Health

Supplementary appendix

This appendix formed part of the original submission and has been peer reviewed. We post it as supplied by the authors.

Supplement to: Johnson HC, Lafferty EI, Eggo RM, et al. Effect of HPV vaccination and cervical cancer screening in England by ethnicity: a modelling study. *Lancet Public Health* 2017; published online Dec 18. http://dx.doi.org/10.1016/S2468-2667(17)30238-4.

S1 Parameterisation

We developed an individual-based model of heterosexual partnership formation, multi-type HPV transmission and disease progression, and cervical screening and vaccination using England-specific data. The model was event-based, meaning that each partnership formation event, disease progression event, screening event was held in a virtual calendar queue. The model runs through these events progressively, following the virtual calendar. Events may be added or cancelled as the model runs. The strains of HPV modelled were 13 high-risk HPV cervical-cancer causing types (HPV-16, -18, -31, -33, -35, -39, -45, -51, -52, -56, -58, -59, -68)[1, 2] and two genital warts-causing low-risk HPV types (-6, -11)[3, 4]. We modelled cervical disease progression towards both squamous cell carcinoma (SCC) and adenocarcinoma (ADC) through the intermediate stages of cervical (glandular) intraepithelial neoplasia (C(G)IN1, C(G)IN2, C(G)IN3), and carcinoma *in situ* (CIS). We also modelled progression of HPV infection to non-cervical cancer. The model was coded in C++ and was run on the Amazon Web Services (AWS) Elastic Compute Cloud (EC2) service as a virtual cluster implemented with a StarCluster platform (version 0.95.6; www.star.mit.edu). Statistical analysis was conducted in STATA v.12 (College Station, TX) and in R (version 3.1.1. 64-bit).

S1.1 Data Sources

National Surveys of Sexual Attitudes and Lifestyles-3

The NATional surveys of Sexual Attitudes and Lifestyles-3 (NATSAL-3) was the third round of a probability survey examining, amongst other things, sexual activity and health and treatment-seeking behaviour[5-8]. Between September 2010 and August 2012, researchers interviewed 15,162 (6,293 men and 8,869 women) British residents between the ages of 16-74. Female respondents who were eligible for cervical screening (aged 26-64) and indicated some sexual experience were asked questions on screening attendance over the past 5 years (5,012 women)[8]. Women eligible for the HPV vaccination programme (n=1,050) were asked if they had been offered the vaccine and how many doses they had received.[7, 8] A subset of surveyed individuals aged 16-44 who reported a minimum of one lifetime sexual partner were invited to submit a urine sample to test for sexually transmitted infections (STI), including 19 strains of high-risk HPV and 2 strains of low-risk HPV [7]. Of 8,047 eligible individuals, 4,550 agreed and had an acceptable sample (56.5%). Survey data was weighted to adjust for sample selection bias, making weighted data comparable to 2011 Census figures in terms of age, gender, marital status, and ethnic origin.

National Chlamydia Screening Programme

Further data on HPV prevalence was obtained from the National Chlamydia Screening Programme (NCSP). Both before (2008) and after (2010-2012) the introduction of the HPV vaccination, residual samples from sexually active 16-24 year old women across England were obtained from the NCSP and tested for 20 strains of HPV[9, 10]. 2,369 and 4,178 samples were tested for each cohort respectively and additional data on participant demographics and sexual behaviour were also collected.

Prevalence survey

A cross-sectional study was conducted by researchers at PHE to determine the prevalence of different types of HPV in different stages of cervical disease in women attending cervical screening (aged 25-64) in England prior to the introduction of the HPV vaccination programme (1986-2008)[11]. Biopsy samples from women with stage 3 cervical intra-epithelial neoplasia CIN3 (n=906), Squamous Cell Carcinoma (SCC) (n=450), Cervical Glandular Intra-epithelial Neoplasia (CGIN) (n=54), or Adenocarcinoma (ADC) (n=105) were obtained from six National Health Service (NHS) pathology laboratories and tested for 37 types of hr and low-risk HPV. Confirmation of cervical disease state was conducted by an experienced pathologist[11].

A Randomised Trial in Screening to Improve Cytology

A Randomised Trial In Screening To Improve Cytology (ARTISTIC) was carried out across four health authorities in greater Manchester from 2001 to 2003 on women aged 20-64 attending routine cervical screening in the English NHS Cervical Screening Programme (NHSCSP)[12-14]. The study was conducted in two rounds that occurred three years apart. In the first round 24,510 women were split randomly into revealed: concealed arms (18,386:6,124 women)[13]. A second round of screening was conducted three years later (2004-2007) to look for incident and undetected disease in 14,230 (58.1%) of the round one women (10,716 revealed and 3,514 concealed)[12].

Incidence of Carcinoma in Situ and Cervical Cancer

Incidence estimates for CIS and cervical cancer per 100,000 women in England were calculated using data on the number of cases of carcinoma in situ of cervix uteri (CiS) and malignant neoplasm of cervix uteri (cervical cancer) by year of age that were reported to the National Cancer Registry at the Office for National Statistics (ONS) in 2011 (using ICD-10 codes - CiS: D06 and cervical cancer: C53). Population estimates for each age were derived from the 2011 Census data for England. To obtain this data we submitted a data request directly to the ONS. Attribution of CiS and cancer cases by HPV type was made according to the proportion of histological cancer samples testing positive for each type (histology data obtained from Public Health England) (Figures S12). We made the limiting assumption of a 'type attribution hierarchy'. That is, cancer was assumed to be caused by the first type present in the sequence HPV-16, -18, -31, -33, -35, -39, -45, -51, -52, -56, -58, -59, -68).

Incidence of Non-Cervical Cancers

Incidence rates for HPV-related non-cervical cancers (anal, oropharyngeal, laryngeal, penile and vulval/vaginal) were also obtained from the ONS National Cancer Registry. We used directly age-standardised and age-specific rates of newly diagnosed cases per 100,000 of the English population, averaged over the years 2010-2014. These data are publicly available online (https://www.ons.gov.uk/peoplepopulationandcommunity/healthandsocialcare/conditionsanddiseases/datasets/cancerregistrationst

atisticscancerregistrationstatisticsengland. The proportions of each of these cancers that was attributable to HPV was obtained from the literature [15], as was the proportion specifically attributable to HPV-16, -18, -31 and -33 [16-18]. The proportions of non-cervical cancers attributable to other hr-HPV types was derived by scaling the remaining HPV-attributable cases by the proportions of these higher-order types found in the cervical histology data obtained from Public Health England (see above).

S1.2 Ethnic classification

In order to increase the number of NATSAL-3 respondents included, a regression analysis was used to classify those reporting themselves as Mixed White/Black, Mixed White/Asian or Chinese into the three modelled ethnic groups (White, Black, Asian) based on common patterns of sexual behaviour, HPV vaccine uptake and cervical screening uptake. Ethnic re-classification is shown in Table S1. Individuals who identified as 'Mixed Other' and 'Other' were not incorporated into the model due to small sample size and non-specific ethnic groupings. The ethnic division of the population in the model was 88.8% White, 3.4% Black and 7.8% Asian as determined by a likewise grouping of 2011 ONS census data for England and Wales. (http://www.ons.gov.uk/ons/rel/census/2011-census/key-statistics-for-local-authorities-in-england-and-wales/rpt-ethnicity.html).

Self-defined ethnic group	Most similar ethnic group		Ethnic group in model
	Sexual behaviour	Vaccine & Screening Uptake	
Mixed White/Black	Black	Black	Black
Mixed White/Asian	White	White	White
Chinese	Asian	Asian	Asian

Table S1 Grouping of ethnicities on the basis of sexual behaviour and vaccine and cervical screening uptake

S1.3 Birth and death rates

Birth rates

Birth rates (Table S2) were derived from ONS data on the 2012 age-specific fertility rates in England and Wales. Birth rates were not assumed to differ by ethnicity and were stratified based on mother's age at time of birth in to the age groups: 15-19, 20-24, 25-29, 30-34, 35-39, and 40-44. For age groups 45+ the birth rate was zero. As new individuals are born in to the model population they are allocated to an ethnicity in a way that conserves the initial ethnic proportions. This is a simplifying assumption which means that a baby's ethnicity does not necessarily reflect the ethnicity of either parent.

Death rates

The total number of deaths were extracted from ONS data for England and Wales on 'Death rates per 1,000 population, by age and sex, 2012, Table 2 and 3' (http://www.ons.gov.uk/ons/publications/re-reference-tables.html?edition=tcm%3A77-325289) and 'Mid-2012 population estimates: Single year of age and sex for local authorities in England and Wales' (http://www.ons.gov.uk/ons/publications/re-reference-tables.html?edition=tcm%3A77-310118). Death rates (Table S2) are gender- but not ethnicity-specific and have been stratified in to the same age groups as birth rates. No specific death rate was given for the 85+ age group as individuals who reached this age were removed from the model.

Age	Annual Birth Rate per 1000 women	Annual Death Rate per 10,000 of population	
		Male	Female
10-14	0	1	1
15-19	19.9	3	1
20-24	69.9	5	2
25-29	105.1	6	3
30-34	113.9	8	4
35-39	63.7	11	6
40-44	14.6	17	10
45-69	0	67	45
70-84	0	418	305
85+	0	N/A	N/A

Table S2 Annual birth and death rates by age group for the population of England and Wales.

S1.4 Sexual début

Age at sexual début (Figure S1) is associated with gender and ethnicity and was derived by fitting a Normal distribution to the responses of NATSAL-3 interviewees aged between 16 and 24 at the time of interview using a least squares approach. We restricted our analysis to these younger respondents to align with those women attending the National Chlamydia Screening Programme, our main source for HPV prevalence data among the young. Due to changing social norms, the age of début for this younger cohort is notably lower than that of the overall NATSAL-3 respondent base, with a more pronounced change in the case of Asians.

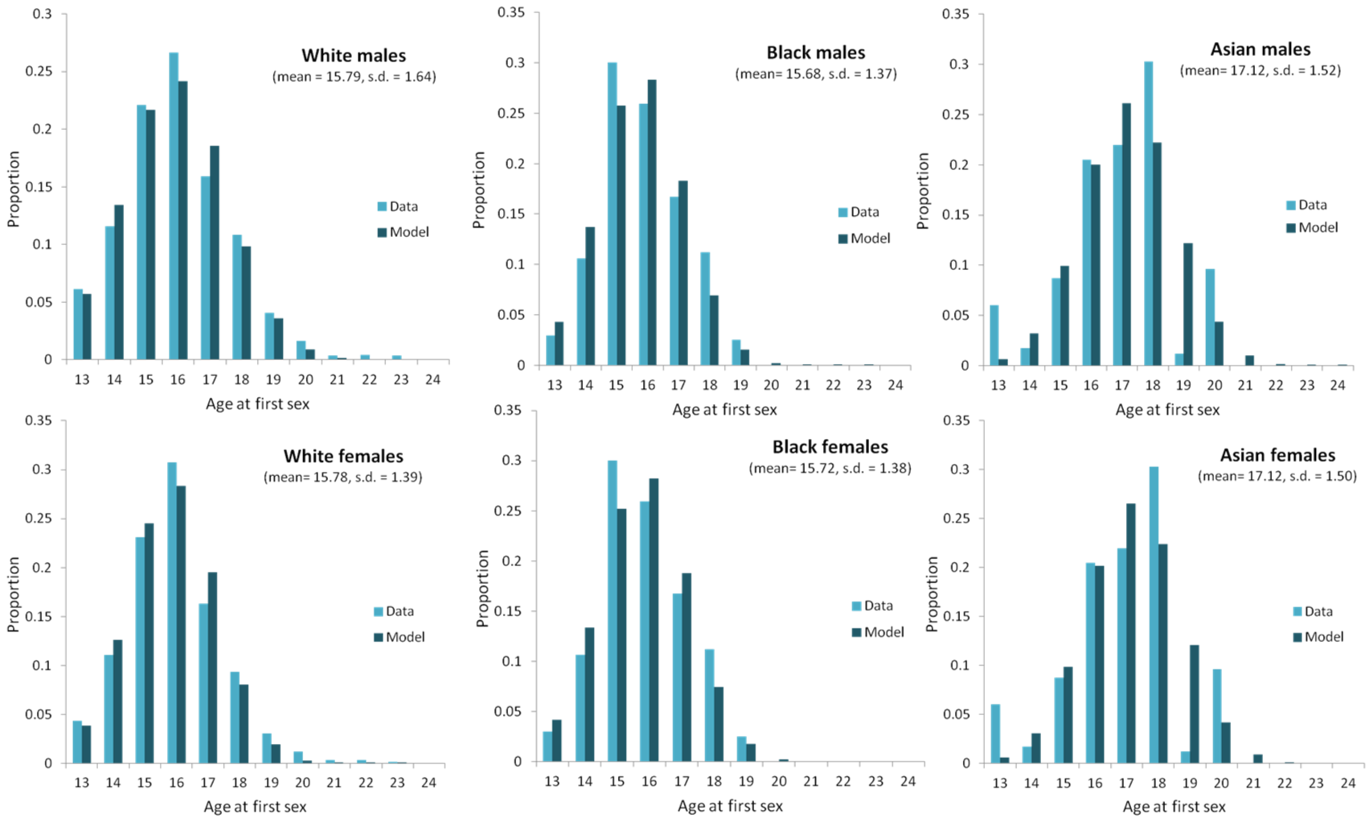

Figure S1 Age of sexual début by gender and ethnicity. A Normal distribution was fitted to the NATSAL-3 responses of 16-24 year olds for each gender and ethnicity group using a least squares method.

S1.5 Rates of partnership formation

		MALES		FEMALES	
		Low sexual activity	High sexual activity	Low sexual activity	High sexual activity
16-18	White	0.93	3.56	0.98	2.57
	Black	1.40	5.37	0.89	2.35
	Asian	0.86	3.30	0.78	2.06
19-21	White	1.01	4.15	1.01	3.23
	Black	1.53	6.27	0.92	2.96
	Asian	0.94	3.85	0.81	2.60
22-24	White	0.81	3.39	0.66	2.86
	Black	1.23	5.17	0.61	2.65
	Asian	0.75	3.15	0.53	2.31
25-29	White	0.71	3.10	0.53	1.84
	Black	1.08	4.68	0.49	1.69
	Asian	0.66	2.87	0.43	1.48
30-34	White	0.42	2.07	0.35	1.97
	Black	0.64	3.16	0.32	1.83
	Asian	0.39	1.93	0.28	1.59
35-39	White	0.31	2.01	0.31	2.01
	Black	0.47	3.06	0.29	1.86
	Asian	0.29	1.87	0.25	1.62
40-49	White	0.20	1.50	0.19	1.81
	Black	0.30	2.30	0.18	1.68
	Asian	0.18	1.40	0.16	1.46
50+	White	0.08	1.15	0.04	1.35
	Black	0.12	1.79	0.04	1.28
	Asian	0.07	1.08	0.03	1.09

Table S3 **Derived rates of partnership formation by gender, age and sexual activity.**

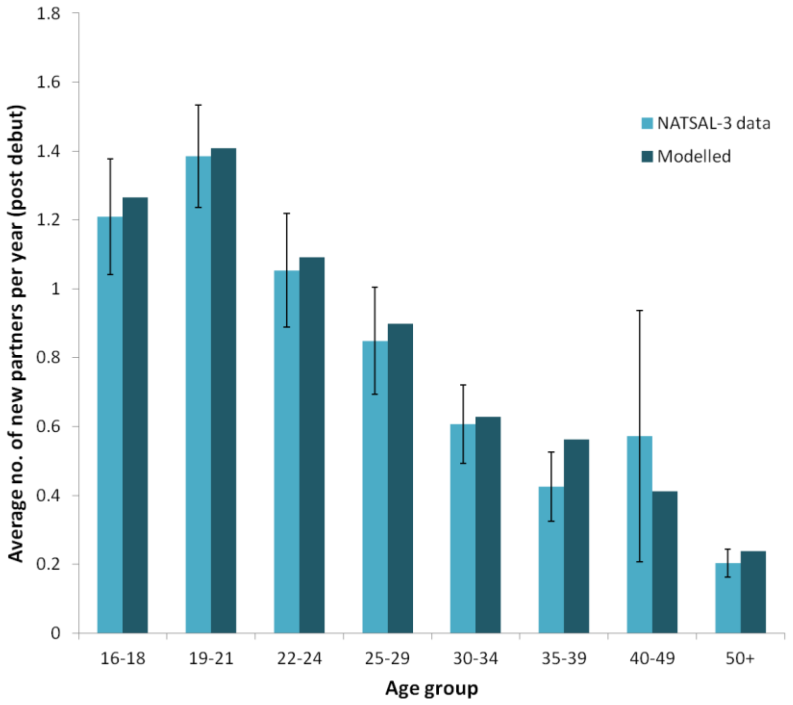

Figure S2 **Average number of new partners within the last year for each age group (excluding respondents who were pre-sexual début), comparing NATSAL-3 data with the modelled rates of partnership formation.**

S1.6 Type of partnership formed by age and sexual activity group

We model three types of non-concurrent heterosexual partnerships: casual, steady and co-habiting (including married). Within NATSAL-3, interviewees were asked to classify their three most recent sexual partnerships in the last five years as casual or steady, whether they anticipated having sex with each of these partners again and whether they were co-habiting. These data were used to specify the proportion of partnerships of each type formed by each age, ethnic and sexual activity group. There are two limitations to this approach. Firstly, in reality a partnership may begin casually before progressing to be classified as steady and then co-habiting. We introduce a bias towards younger age groups forming more steady partnerships because we take the interviewee's classification today of a partnership that may have been formed when they were somewhat younger. Secondly, a partnership classified by a 16-year old as 'steady' may have rather different characteristics to one thus classified by a 23-year old. We mitigated this effect by specifying the partnership duration according to the age of the female partner at its commencement. The choice of age group and ethnicity of male partner is also considered to be dependent on the age of the female partner at its outset. Where no data was available due to low sample numbers for some age groups of Black or Asian ethnicities, the proportion for White people at that age was substituted. Figure S3 shows the proportion of casual, steady and co-habiting partnerships formed by each group.

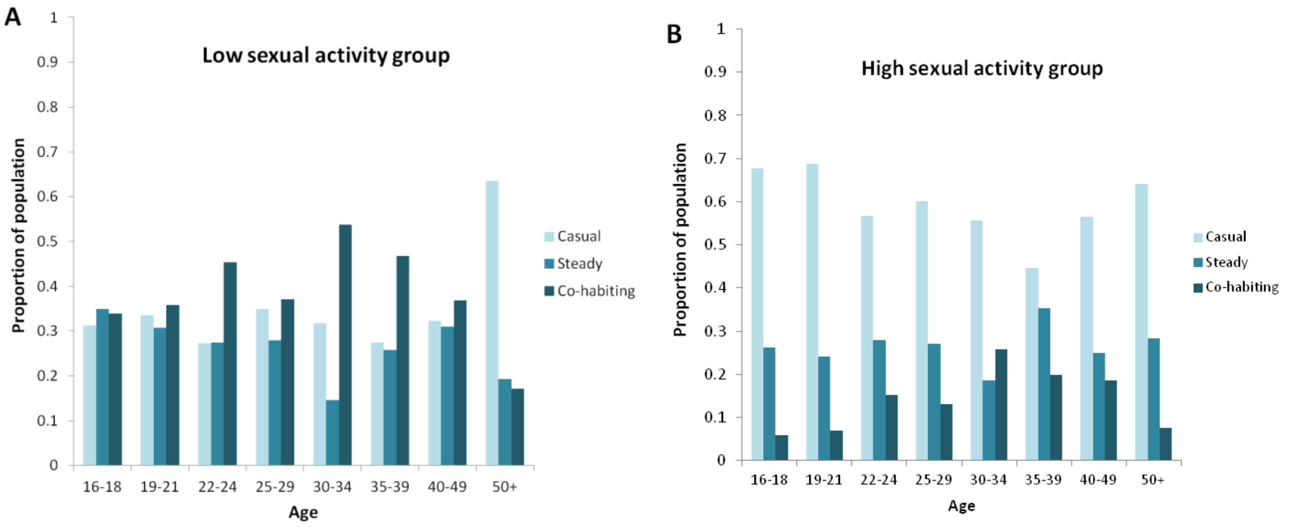

Figure S3 The proportion of partnerships identified as casual, steady, or co-habiting for each age group, separated into (A) low and (B) high sexual activity groups.

S1.7 Partnership duration

A distribution of partnership duration, dictated by the age of the female at the time of partnership formation, was determined for each partnership type. We made the limiting assumptions that the baseline risk of a partnership ending is constant over time and that the duration of partnerships formed at a certain age does not vary over time. Thus the survival fraction for the partnership is given by the Exponential function:

$$S(t) = e^{-\lambda t}$$

where λ is the hazard function. We estimated λ for partnerships of each type formed by individuals in each age group using data from NATSAL-3 and a maximum likelihood approach.

The maximum likelihood estimator is defined as:

$$\hat{\lambda} = \frac{total\ number\ of\ partnerships\ ended}{exposure\ time}$$

The issues in estimating the distribution of partnership durations from empirical data have been described elsewhere [19 20]. Briefly, we addressed three sources of bias: right-truncation bias due to ongoing partnerships, and length-biased sampling (or left-truncation bias (Simon, 1980)) due to both fixed window sub-sampling and fixed number of partners sampling. Right-truncation bias is accounted for expressly by using a Cox proportional hazards model.

Fixed window sub-sampling, where interviewees are asked to recall their partnerships within e.g. the past five years, incorporates a lengthening bias since longer partnerships are more likely to be included within the sampling window. Since limiting our analysis to partnerships that began within the sampling window would have led to the exclusion of many data (particularly for older age groups), we followed the approach of Burington et al. (2010) in modifying the maximum likelihood estimator $\hat{\lambda}$ to account for left-truncation.

Where interviewees are asked to recall only the most recent *n* partnerships, as in NATSAL-3, it is important to account for another source of length-biased sampling. Since we consider only the three most recent partnerships, we are excluding proportionally more data from those respondents who have a higher number of partners over not only the last five years, but over the lifetime. If partnership duration is associated with number of partners, as it is reasonable to assume, this will also lead to an overestimate of length of partnerships. To account for this, we used an inverse probability weighting approach following the primary weighting scheme of Copas et al. (2009). Since we have data from NATSAL-3 on the lifetime number of partners, we extend our estimation of partnership duration to account for the earlier 'missing' partnerships for which we do not have explicit data on duration. We derived weights scaled to the total number of partners within respondents, grouped by 1, 2, 3 and 4 or more. Partnerships reported by respondents with 3 partnerships or fewer were weighted as 1 and ongoing partnerships were also weighted as 1. Missing partnerships which had ended were assumed to be similar to reported partnerships that had ended and thus the latter were weighted as:

$$w = \frac{total\ no.of\ partnerships\ ended}{no.of\ reported\ partnerships\ ended}$$

We conducted the survival analyses using the 'counting process' style of input in STATA.

The estimated values of λ for females in each age group and partnership type are given in Table S4 and the corresponding survival fractions calculated are plotted in Figure S4, which illustrates the distribution of partnership duration by age group,.

When a new partnership is formed, its duration is stochastically determined. When a partnership endpoint is reached, both partners re-join their respective partnership formation trees. Partnership duration was not split by sexual activity level as this is automatically incorporated due to high sexual activity individuals being more likely to form casual partnerships and therefore forming new partnerships more frequently, naturally decreasing this group's partnership duration.

Female age when partnership commences	Hazard Function (λ)		
	Casual partnership	Steady partnership	Co-habiting partnership
16-18	1.012	0.282	0.021
19-21	2.931	0.314	0.021
22-24	1.445	0.288	0.027
25-29	1.220	0.289	0.075
30-34	3.161	0.319	0.028
35-39	0.947	0.232	0.033
40-49	1.247	0.436	0.174
50+	0.919	0.343	0.032

Table S4 The maximum likelihood estimate of the hazard function (λ) for partnerships ending, according to the age group of the female partner when the partnership commenced.

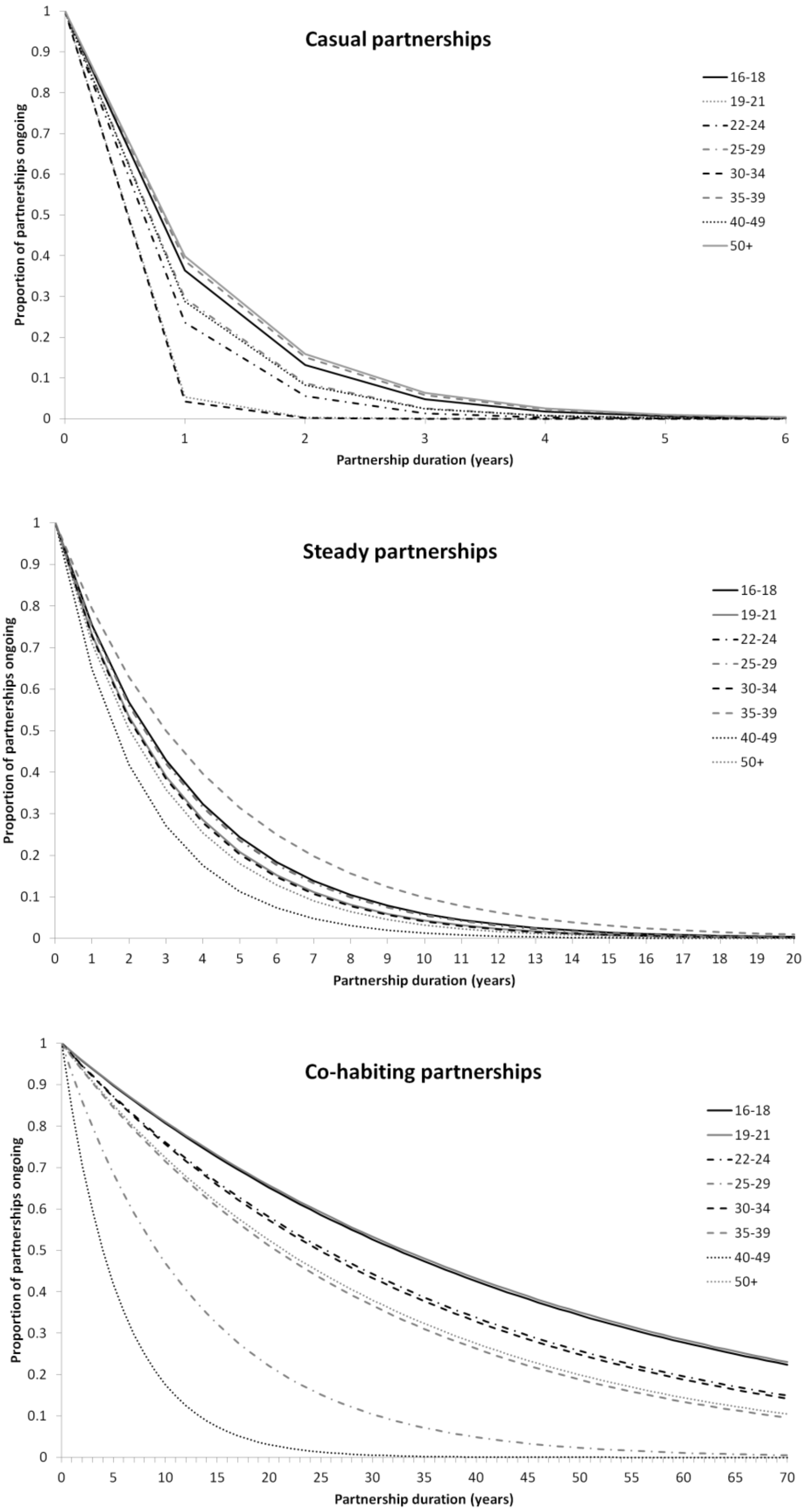

Figure S4 **The proportion of partnerships ongoing after a certain number of years according to the age group of the female partner at time of partnership formation. Data was derived from the NATSAL-3 study for partnerships defined as (A) casual, (B) steady and (C) living together/married.**

S1.8 Mixing matrices (casual, steady, modelled) for each age and ethnicity group

Using NATSAL-3 data on the ethnicity and age of the three most recent sexual partners in the preceding five years of the respondent we derived separate mixing matrices for casual and steady partnerships based on 'female choice'. That is, we defined the proportion of new partnerships that females of a certain ethnicity and age group form with males of each ethnicity and age group. For mixing matrices, we combined the steady and living together/married classifications used for partnership duration into a single steady group in order to reduce stochastic effects arising from small numbers in less common age/ethnicity pairings. We extracted data in larger age bands (13-21, 22-34, 35+) for a similar reason. In the same way as for partnership duration, we defined the individual's age as their age at the time of partnership formation. By considering the three most recent partners within five years, we perhaps introduced bias towards the mixing preferences of those who had a higher number of partners within that timeframe but this was deemed acceptable in order to increase the number of partnerships analysed and thus further reduce stochastic effects that arise due to small numbers in less common age/ethnicity pairings. Figure S5 depicts the mixing matrices used in the model. Each sub-figure shows the ethnicity and age distribution of male partners on the x-axis chosen by a female of a given age and ethnicity for either a steady or casual partnership. Figure S5a shows the distribution of partnerships by age and ethnicity compared with the distribution derived from the data. The proportion of partnerships defined as casual vs. steady is determined by the age and sexual activity level of an individual.

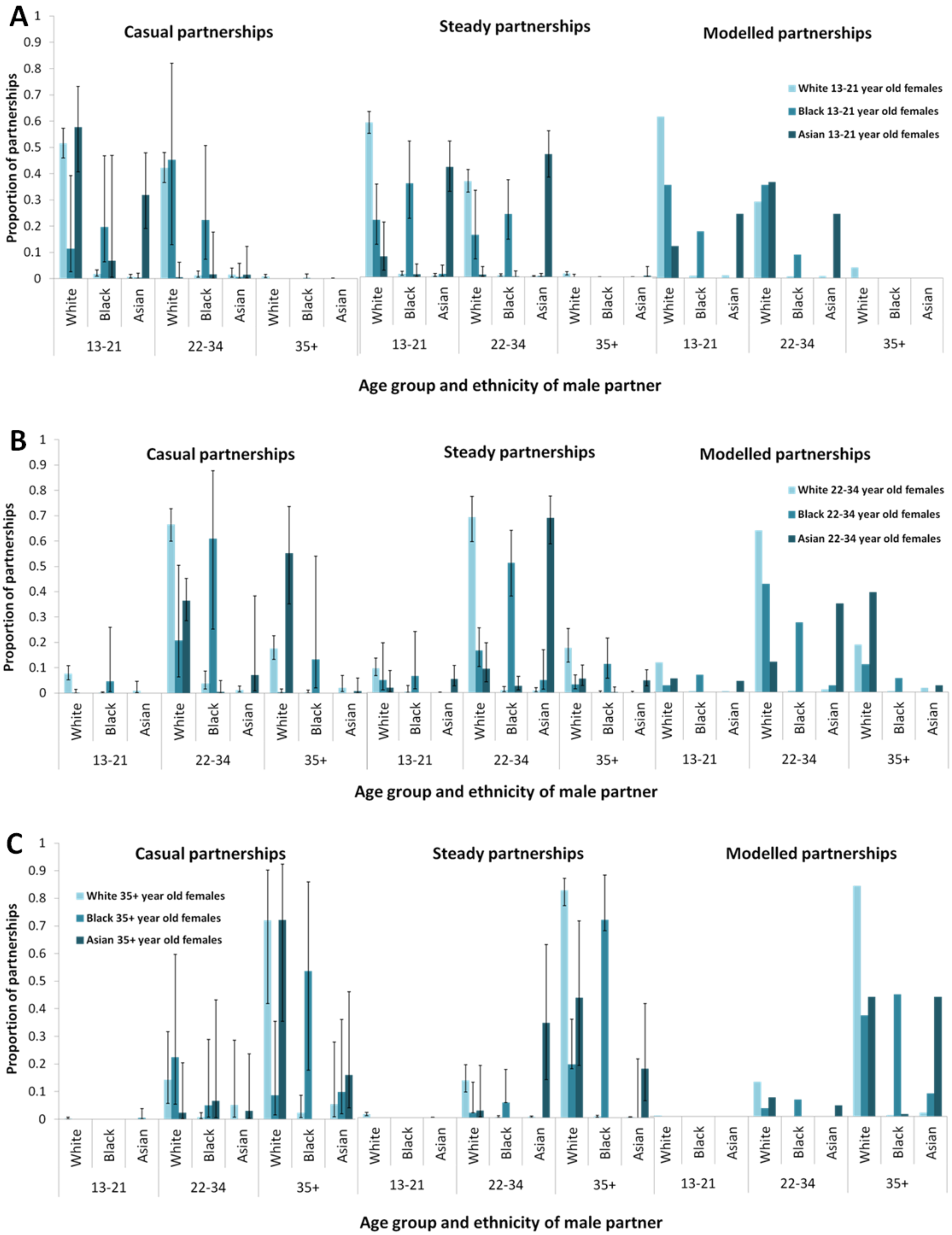

Figure S5 Mixing matrices by partnership type: A) casual; B) steady; C) the actual distribution of modelled partnerships

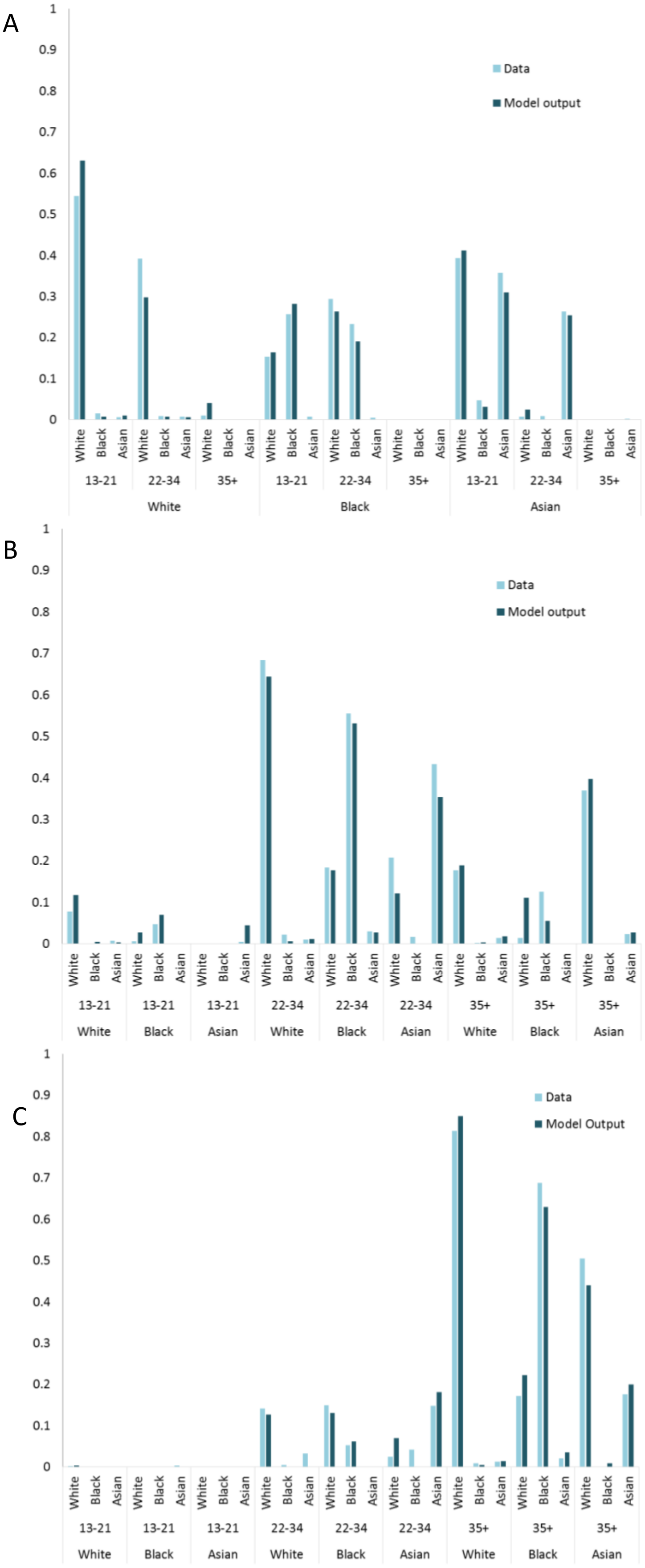

Figure S5a Modelled partnerships by age and ethnicity compared with observed data (NATSAL-3). Plots show the proportion of partnerships formed by females aged A) 13-21 years, B) 22-34 years and C) 35 years and older with male partners by age of partner and ethnicity.

S1.9 Frequency of sex

By definition, individuals have sex at the point of beginning a partnership. The frequency with which individuals who are in a partnership have sex was specified by fitting a truncated Normal distribution to the NATSAL-3 data (mean = 1.98, s.d. = 3.5 sex acts per month) using a least squares approach. We made the limiting assumption that it was the same for all individuals in the population, regardless of age, ethnicity, sex and partnership type. Figure S6 shows the fit of the model to data.

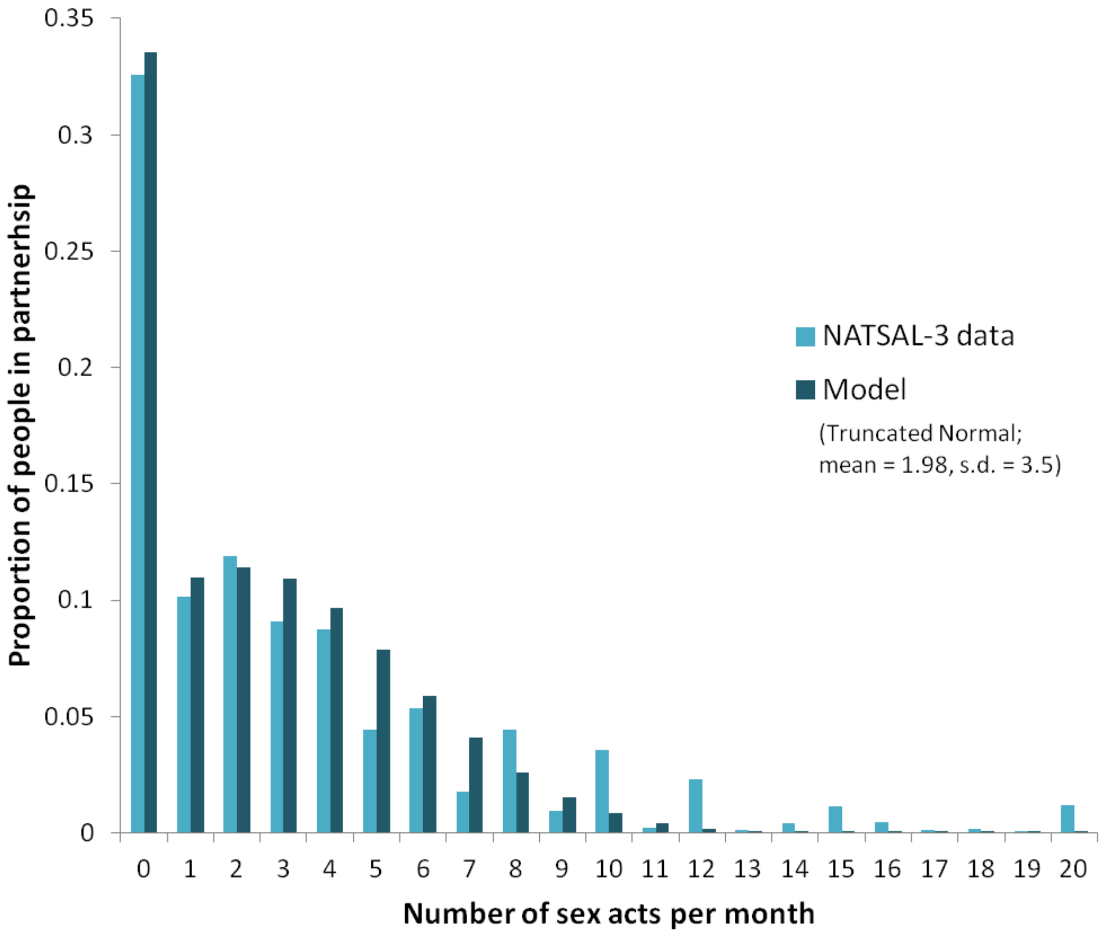

Figure S6 **Distribution of number of sex acts per month for those in an ongoing sexual partnership.**

The distribution of frequency of sex within a partnership is used to determine the time lag before another sex act. Once a sex event has been logged, a number is drawn from the frequency distribution and inverted to give the lag before the next sex act. If a 0 is drawn then a lag of 30 days is assumed, another frequency is drawn and its inverse is added to the original 30 days. Therefore the frequency of sex varies stochastically throughout a partnership.

S2 Natural history

We modelled the transmission and disease progression of 13 high-risk HPV types (-16, -18, -31, -33, -35, -39, -45, -51, -52, -56, -58, -59 and -66) as well as HPV-6 and -11. Figure S7 provides a schematic representation of the natural history structure for a high-risk HPV type. Each individual modelled occupies a health state, with respect to each HPV type, corresponding to a compartment in Figure S7. The use of an individual-based model allows each individual to be concurrently infected with any number of HPV types, each at a different stage of disease progression. In each time step, an individual has the chance to move to a new health state by a stochastic process with probability determined by the relevant force of infection, progression or clearance rate. Infection, progression and clearance rates were deemed to be independent between HPV types and we assumed that all rates are independent of age, gender, ethnicity, sexual activity and personal history of HPV infection.

We modelled the spontaneous clearance of initial infection and CIN1 lesions but assumed that CIN 2+ lesions cleared only as the result of treatment. This is a limiting assumption.

Each type of high-risk HPV may progress to one of two types of cancer: squamous cell carcinoma (SCC) or Adenocarcinoma (ADC). The intermediate stages of disease are modelled as Cervical Intraepithelial Neoplasia (CIN1-3) and Glandular Cervical Intraepithelial Neoplasia (CGIN1-3) respectively. Of those women who develop cervical disease, the fraction progressing towards SCC vs. ADC is determined by the proportion of cancer type attributed to each HPV type (Howell-Jones et al., see Figure S11). We assumed that the rates of progression through the intermediate stages to squamous cell carcinoma and Adenocarcinoma were the same.

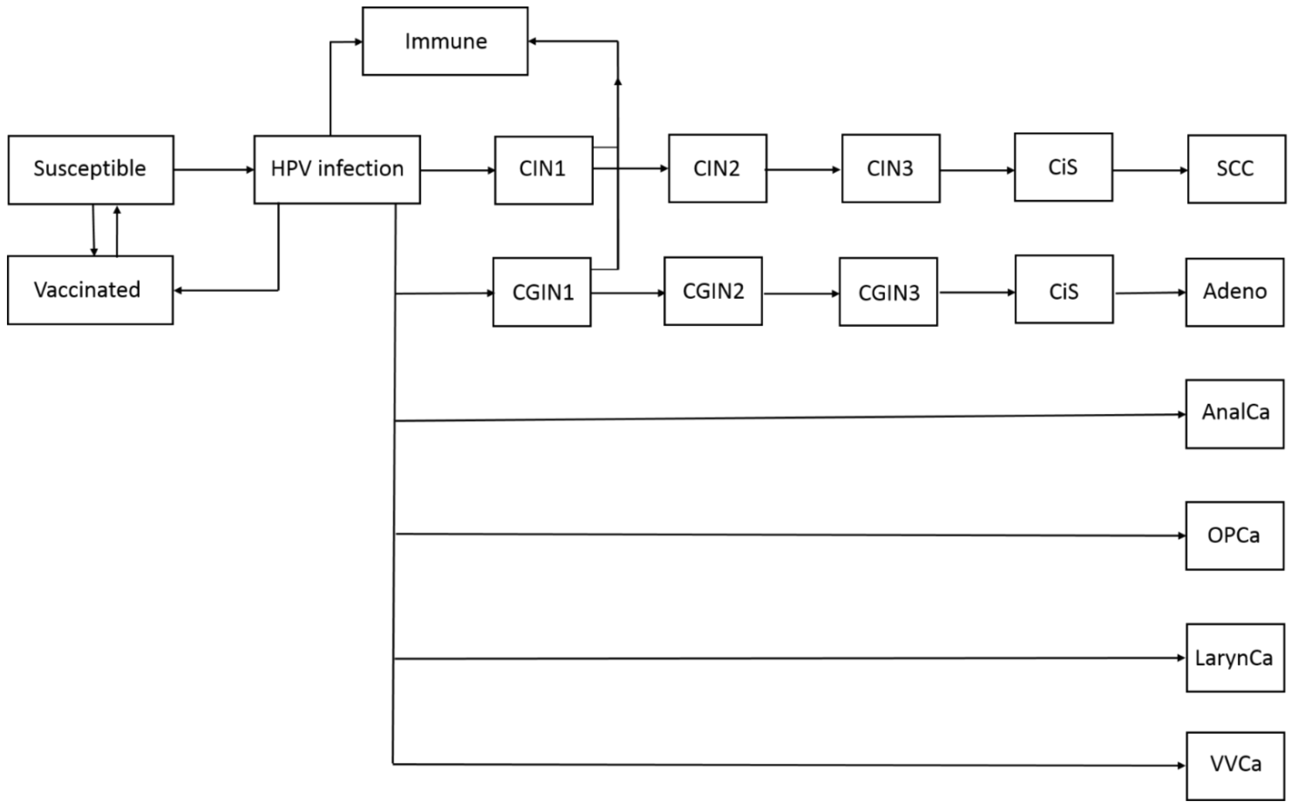

Figure S7 Natural history of high-risk HPV infection for females. Initial infection may either clear spontaneously, rendering the individual immune, or progress to cervical disease or to another form of cancer (AnalCa: anal cancer; OPCa: oropharyngeal cancer; LarynCa: laryngeal cancer; VVCa: vulval or vaginal cancer). Cervical disease can lead to either squamous cell carcinoma (SCC) or adenocarcinoma (Adeno) of the *cervix uteri*, progressing through a series of neoplastic stages (CIN1..3 or CGIN1..3, respectively) and carcinoma *in situ* (CiS).

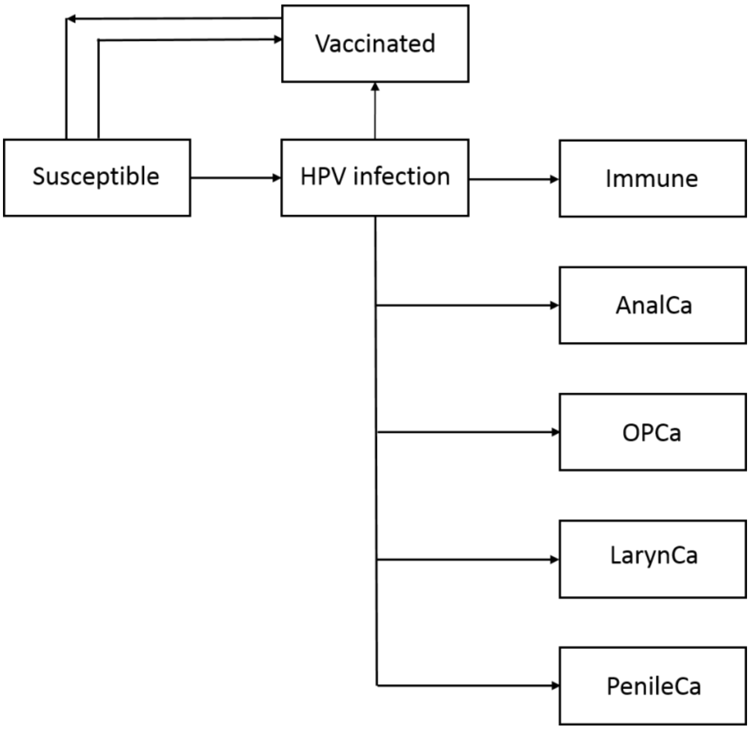

Figure S8 Natural history of high-risk HPV infection for males. Initial infection may either clear spontaneously, rendering the individual immune, or progress to cancer (AnalCa: anal cancer; OPCa: oropharyngeal cancer; LarynCa: laryngeal cancer; PenileCa: penile cancer).

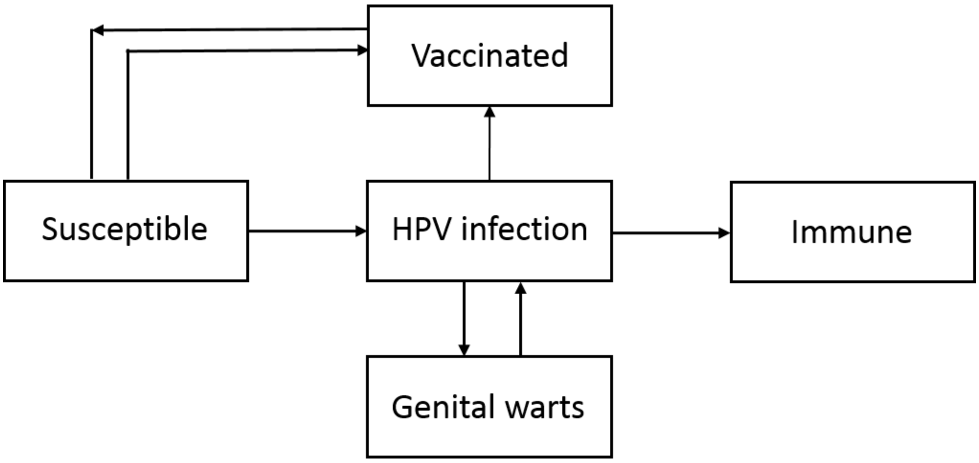

Figure S9 Natural history of low-risk HPV infection. Infection may cause recurrent episodes of symptomatic genital warts.

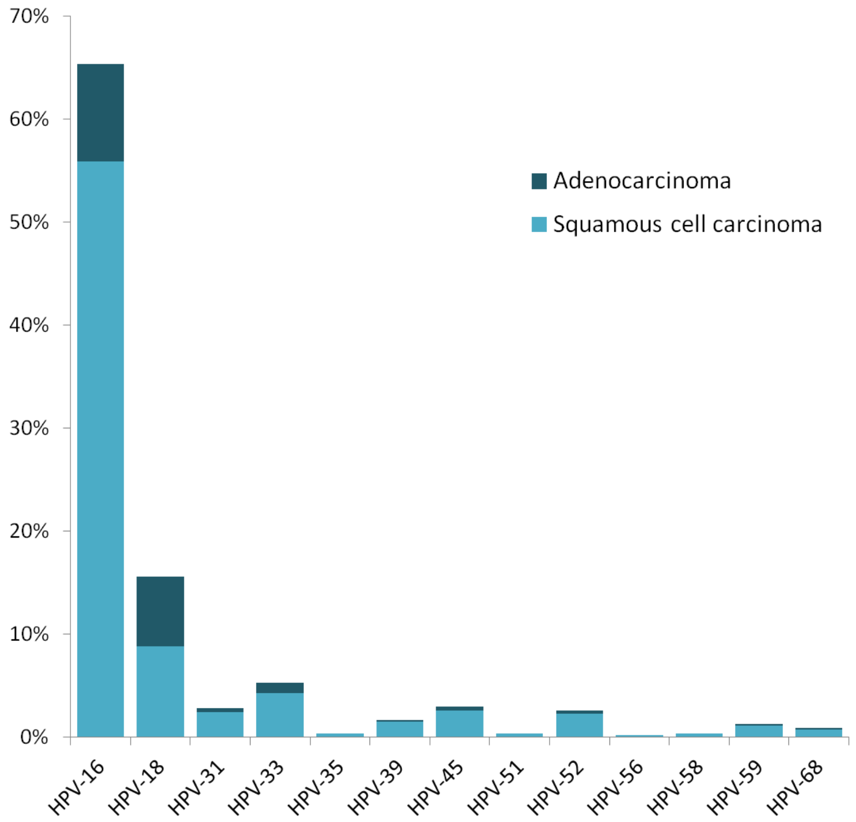

Figure S10 **The attribution of cancer cases to each of the HPV types according to their presence in histological samples held by Public Health England. We assumed a causal hierarchy; where more than one type was present, the cancer was assumed to have been caused by the lowest numbered type.**

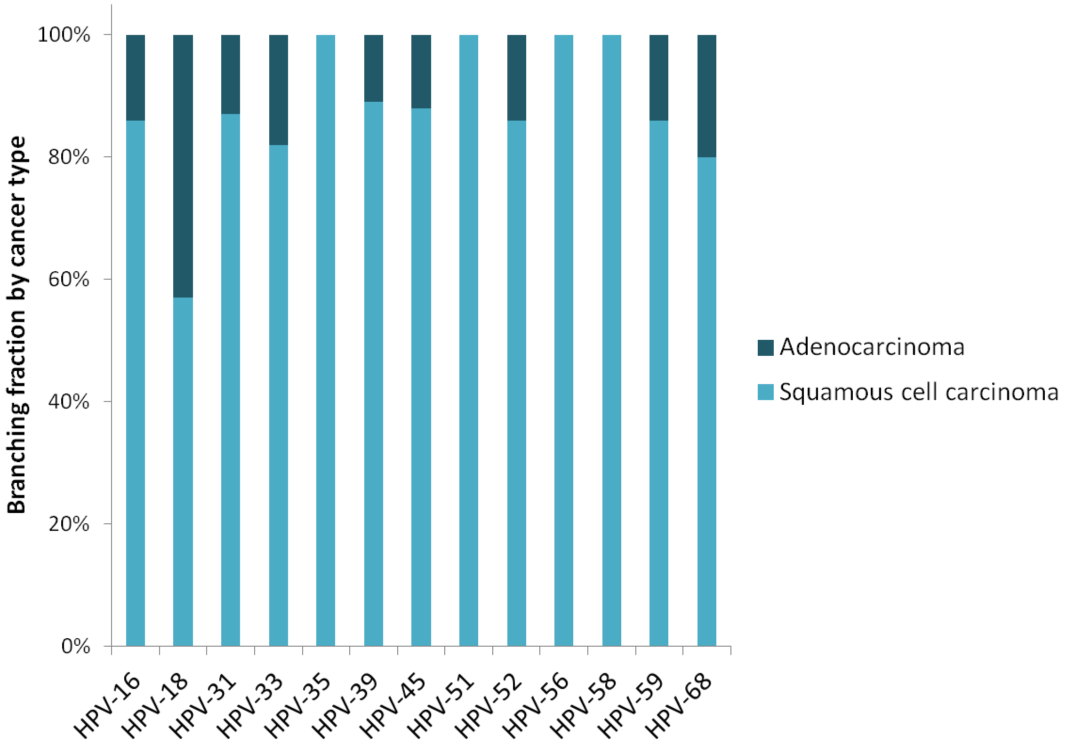

Figure S11 **The branching fractions of each high-risk HPV type towards Squamous Cell Carcinoma vs.Adenocarcinoma.**

Incidence of Non-Cervical Cancers

Incidence rates for HPV-related non-cervical cancers (anal, oropharyngeal, laryngeal, penile and vulval/vaginal) were also obtained from the ONS National Cancer Registry. We used directly age-standardised and age-specific rates of newly diagnosed cases per 100,000 of the English population, averaged over the years 2010-2014. The proportions of each of these cancers that was attributable to HPV was obtained from the literature, [15] as was the proportion specifically attributable to HPV-16, -18, -31 and -33. [16-18, 21, 22] The proportions of non-cervical cancers attributable to other high-risk HPV types was derived by scaling the remaining HPV-attributable cases by the proportions of these higher-order types found in the cervical histology data obtained from Public Health England. The attributability of non-cervical cancers is given in Table S5.

	**Age-adjusted cancer incidence per 100,000 population**	**Overall % attributable to HPV**	**% of HPV-attributable cancers caused by type**												
**FEMALE**			16	18	31	33	35	39	45	51	52	56	58	59	68
Anal	62.0	89%	83.1%	7.8%	1.4%	2.5%	0.2%	0.81%	1.4%	0.2%	1.3%	0.1%	0.2%	0.6%	0.5%
Oropharyngeal	213.7	28%	86.8%	2.8%	1.5%	2.9%	0.2%	0.93%	1.6%	0.2%	1.4%	0.1%	0.2%	0.7%	0.5%
Laryngeal	33.5	21%	82.0%	16.0%	0.3%	0.6%	0.0%	0.18%	0.3%	0.0%	0.3%	0.0%	0.0%	0.1%	0.1%
Vulval/vaginal	139.3	40%	70.4%	25.9%	0.6%	1.0%	0.1%	0.33%	0.6%	0.1%	0.5%	0.0%	0.1%	0.3%	0.2%
**MALE**			16	18	31	33	35	39	45	51	52	56	58	59	68
Anal	41.3	86%	83.0%	8.0%	1.3%	2.5%	0.2%	0.81%	1.4%	0.2%	1.3%	0.1%	0.2%	0.6%	0.4%
Oropharyngeal	469.2	28%	86.8%	2.8%	1.5%	2.9%	0.2%	0.93%	1.6%	0.2%	1.4%	0.1%	0.2%	0.7%	0.5%
Laryngeal	173.5	21%	82.0%	16.0%	0.3%	0.6%	0.0%	0.18%	0.3%	0.0%	0.3%	0.0%	0.0%	0.1%	0.1%
Penile	63.9	40%	60.0%	3.3%	1.7%	9.5%	5.0%	3.06%	6.7%	0.7%	3.3%	0.3%	0.7%	2.4%	3.3%

Table S5 Attributability of non-cervical cancer to HPV by type

S3 Cervical screening

S3.1 Cervical screening schedule and uptake

The model invites all women for routine cervical screening according to NHS guidelines (http://www.nhs.uk/Conditions/Cervical-screening-test/Pages/Introduction.aspx) with first invitation at age 25 and subsequent invitation every three years until age 49. Women aged 50-64 are invited for a cervical screening test every five years.

In the model, women are classified as full, partial or non-attendees of cervical screening according to NATSAL-3 data on whether they were up-to-date with their screening, had ever been screened, or had never been. Figure 1 in the main paper text compares uptake of cervical screening by ethnicity between NATSAL-3 and Public Health England. The latter shows a lower uptake across all ethnic groups and a larger discrepancy between the White group and Black and Asian.

In the model, a female is called for her first cytological screening appointment at age 25 and the model sets a date for her to attend this screening appointment. The chance that this female is a full, partial, or non-attendee is determined by her ethnicity and is independent of her age. A full attendee goes to the scheduled appointment (allowing for a random delay of up to a year), a non-attendee skips it and partial attendees have a 50% probability of attending any given appointment. If a screening appointment is skipped, the next screening call is set based on the timing of routine screening that is appropriate for the individual's age.

Following a screening appointment, the model logs the woman's cervical disease stage (from susceptible to cancer). If the woman is negative, a random number is drawn and compared against the diagnostic test specificity to allow for the diagnosis of false positives. If the woman is disease stage positive, a similar process stochastically determines if she will be identified as false negative, thereby missing treatment.

If a woman does not show signs of disease at her routine screening appointment she is called for another appointment three or five years later, depending on her age. In the case of a disease diagnosis (C(G)IN1 or higher) she moves from the routine screening schedule to a testing algorithm (Figure S12) adapted from the 'NHS Cervical Screening Programme Screening Protocol Algorithm for HPV Triage and Test of Cure' (http://www.cancerscreening.nhs.uk/cervical/hpv-triage-test-flowchart-201407.pdf). Following treatment and/or disease clearance, individuals resume routine screening and move to the immune stage of the HPV strain that caused the presentation of disease.

S3.2 Sensitivity and specificity of cervical screening

A literature search was conducted to determine realistic estimates of the sensitivity and specificity of cervical testing in the UK. Search criteria were confined to UK-based studies as diagnostic techniques can be subjective based on technician training and skill. This ensured that estimates were applicable to the modelled population.

Disease stage	Sensitivity	Specificity
CIN1 (including borderline dyskaryosis)	76.6%[23]	95.8%[23]
CIN2+	82.1%[24]	-
CIN3+	82.9%[24]	-

Table S6 The modelled values for the sensitivity and specificity of routine cervical screening to detect cervical disease of a given stage.

We assumed specificity of identifying CIN2+and CIN3+ to be 100%.

Sensitivity and specificity estimates for colposcopy were assumed to be 100%. Colposcopy is considered the gold standard of cervical disease diagnosis therefore if disease is not detected using this test, it is presumed that no disease exists.[25]

S3.3 Cervical screening in vaccinated populations

Early predictions for the impact of the HPV vaccination on cervical screening uptake suggested that increased rates of cervical screening attendance among vaccinated girls could be an artefact of the catch-up cohorts (who may have sought out the vaccine and therefore be characterised by high engagement with healthcare provision). [26, 27] However, a school-based survey of girls from the first two routine cohorts assessed screening intention by vaccination status, [28] finding a comparable odds ratio for intending to screen having been vaccinated (1.6; 95% CI: 0.5-2.9) as had been observed in screening attendance in Wales (1.7; 95% CI: 1.6-1.8)[26] and Scotland (1.5; 95% 1.5-1.6),[27] where women were invited for screening from age 20.

S3.4 Cervical screening algorithm

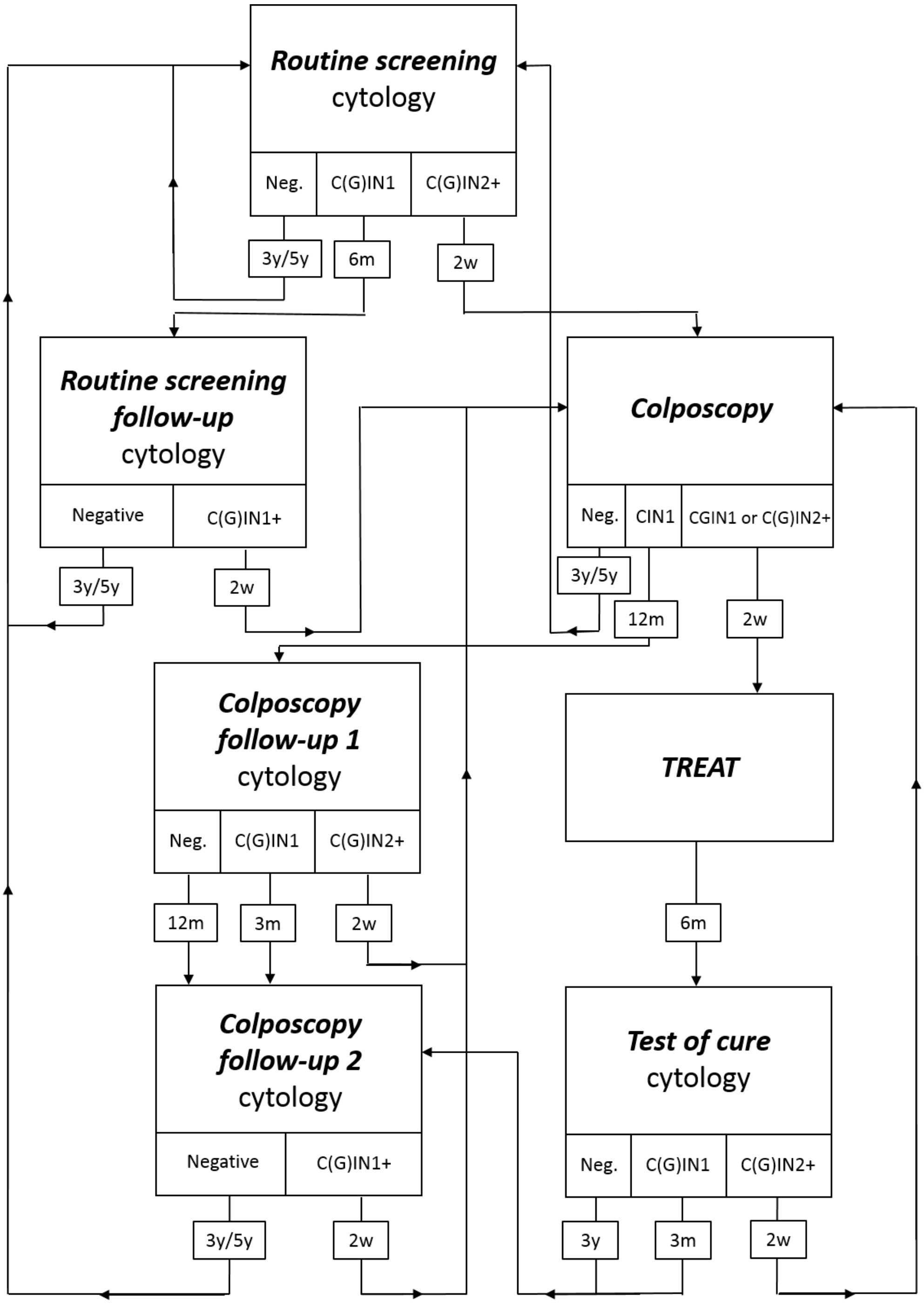

Figure S12 **Screening algorithm following NHS cervical screening programme**

S4 Vaccination

S4.1 Uptake

A study examined vaccine uptake in both the routine and catch-up cohorts in England between 2008-2011 (Louie, unpublished 2015). Data from 123,415 females were used to assess whether ethnicity, deprivation, smoking, cervical screening attendance and sexual behaviour were associated with vaccine uptake. Data were obtained from eight child health databases stored at Primary Care Trusts (PCTs; abolished in England in 2013, data now stored by local authorities) and the Clinical Practice Research Datalink (CPRD). Girls were split into three groups by age: a routine (aged 12-13) group and two catch-up groups (aged 14-15 and 16-18) and their vaccination status was coded as fully vaccinated, initiated vaccination or unvaccinated (Louie, unpublished 2015). Combining data sets revealed that in the routine cohort more than 80% and 70% of girls had initiated or completed HPV vaccination, respectively. In the 16-18 year-old catch-up cohort however, coverage was much lower with fewer than 60% of girls initiating HPV vaccination (Louie, unpublished 2015). In all cohorts White girls were more likely to have initiated or completed vaccination than girls from Black, Asian or 'Other' ethnicities (Louie, unpublished 2015). Ethnicity-specific vaccination uptake, as obtained from the Primary Care Trusts and used in the model, is presented in Figure 1B. The CPRD data (Figure 1C) showed a lower uptake of HPV vaccine.

S4.2 Cross-protection

We modelled cross-protection of the bivalent and quadrivalent vaccines against six non-vaccine types. Vaccine efficacy was derived from the literature and is shown in Table S7. [29, 30] We assumed that the duration of cross-protection was 10 years, shorter than that against vaccine types.

	HPV-31	HPV-33	HPV-45	HPV-51	HPV-52	HPV-58
Bivalent	0.771	0.431	0.79	0.255	0.189	0
Quadrivalent	0.462	0.287	0.078	0	0.184	0.055

Table S7 Cross-protective efficacy of the bivalent and quadrivalent HPV vaccines against HPV-31, -33, -45, -51, -52 and -58.

S5 Combining HPV prevalence sources

To account for the differences between the subpopulations from which the HPV prevalence data were derived, and their associated biases, we employed a Bayesian evidence synthesis approach to estimate the true underlying prevalence in the population. The main sources of prevalence data, NCSP and ARTISTIC, were sampled from a subpopulation that was both sexually active (by which we mean post-début) and attending Chlamydia testing or cervical screening respectively. The inherent bias is therefore particularly strong in the data for 16-24 year olds (NCSP), where the proportion of females who are sexually active is increasing from 34% to 97%; of these only ~50% are attending the Chlamydia testing programme. By using data from NATSAL-3 on the age of sexual début, the proportion of sexually active females who had been tested for Chlamydia within the last year (for 16-24 year olds) or who had recently attended a routine cervical screening appointment (25-44 year olds) and the ratio of HPV prevalence between the tested/screened population and the untested/unscreened population, we were able to reconstruct a population tree that included the non-active, non-tested sub-populations (Figure S13). We estimate the prevalence among the active but untested/unscreened population to be

$$\varepsilon = \delta * \frac{\text{prev. among active \& unscreened (NATSAL)}}{\text{prev. among active \& screened (NATSAL)}}$$

where δ = HPV prevalence in the active and tested/screened population. This is scaled by the ratio in prevalence between NATSAL respondents who are active and untested/unscreened and those who are active and tested/screened. The process was repeated for each single year of age.

We estimated the prevalence in the underlying population using a Markov Chain Monte Carlo (MCMC) approach with prior ~ Uniform[0,1] and a Binomial log-likelihood.

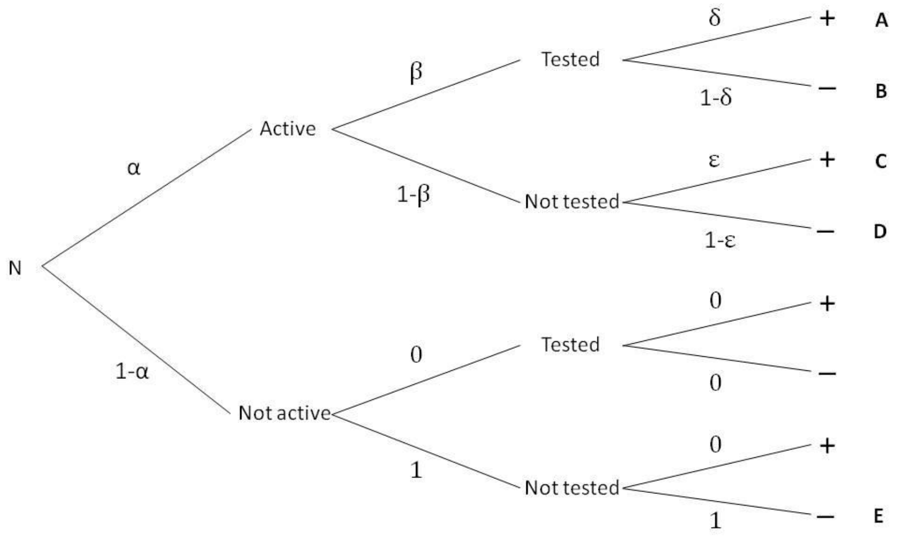

Figure S13 A population tree showing the reconstruction of the underlying population. Prevalence data from the NCSP and ARTISTIC sources is equivalent to A /(A + B). We estimate the true prevalence based on (A + C) / (A + B + C + D + E). The branching fractions are α: proportion of population which is sexually active; β: proportion of the active population that attends CT testing (16-24 year olds) or cervical screening (25-44 year olds); δ: type-specific HPV prevalence among the active and tested/screened population (from NCSP/ARTISTIC); ε: type-specific HPV prevalence among the active but untested/unscreened population. We assumed that the non-active population attended neither Chlamydia testing nor cervical screening and that HPV prevalence among this sub-group was zero.

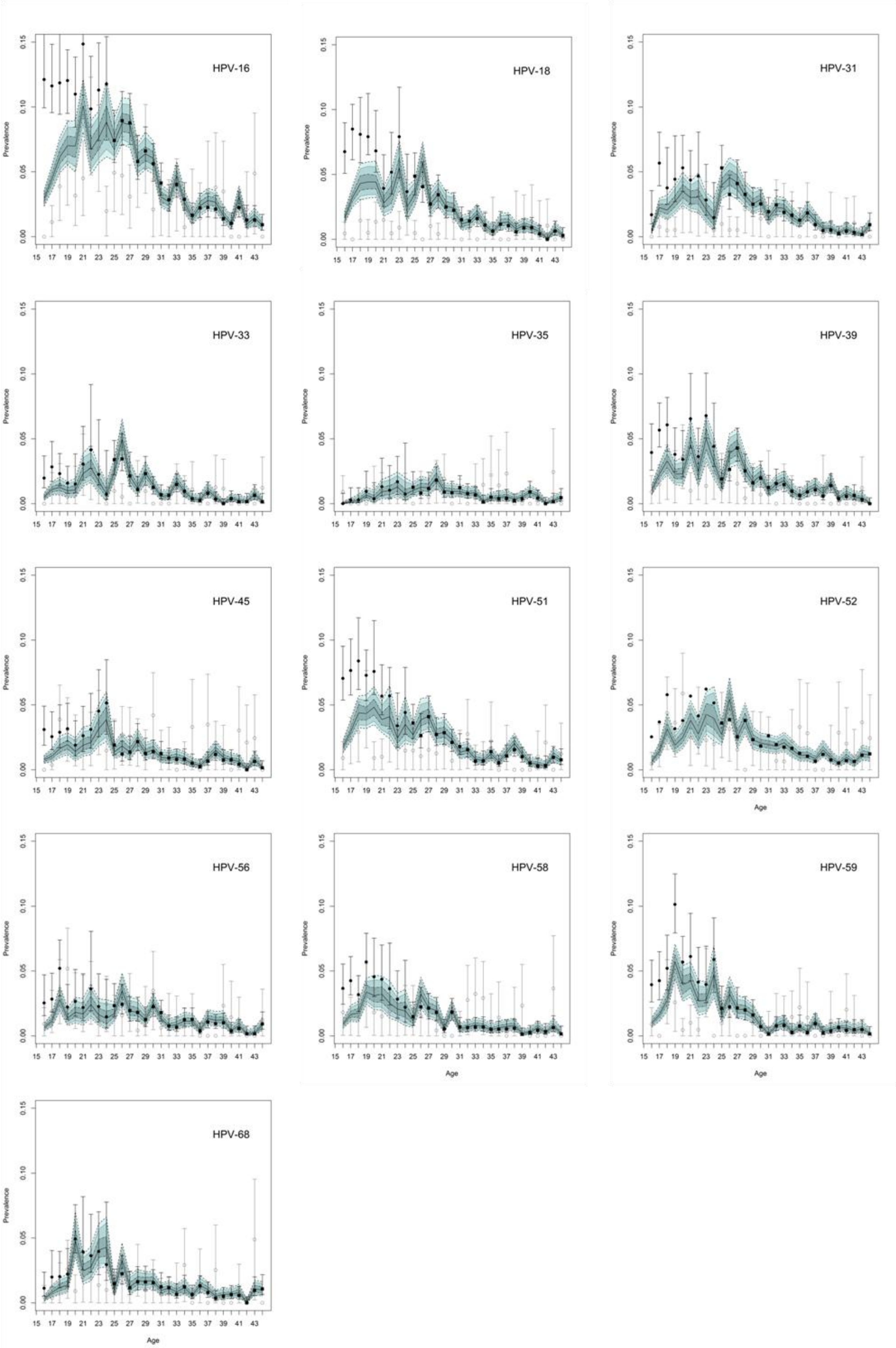

Figure S14 Estimated prevalence of high-risk HPV in females, by type. We adopted a Bayesian evidence synthesis approach to combine prevalence data from the National Chlamydia Screening Programme (black circles), the ARTISTIC trial (black squares) and NATSAL-3 (hollow circles) in a way that allowed us to account for the inherent biases in the data. The median predicted prevalence for each type is represented by the solid curve and the turquoise bands represent the 50%, 70%, 90% and 95% posterior intervals. This median predicted prevalence then became the model-fitting target for the individual-based model.

S6 Sensitivity analysis: lifelong protection of the vaccine

In the main analysis we assumed that both the bivalent and quadrivalent vaccines offered 20 years of protection. Here we present the results of a sensitivity analysis modelling lifelong vaccine protection from both vaccines. Figure S15 shows the rate ratios of cervical cancer incidence comparing A) Asian women with White women and B) Black women with White women. We do not observe a statistically significant difference in inequality over time when comparing our base case scenario with that of lifelong vaccine protection.

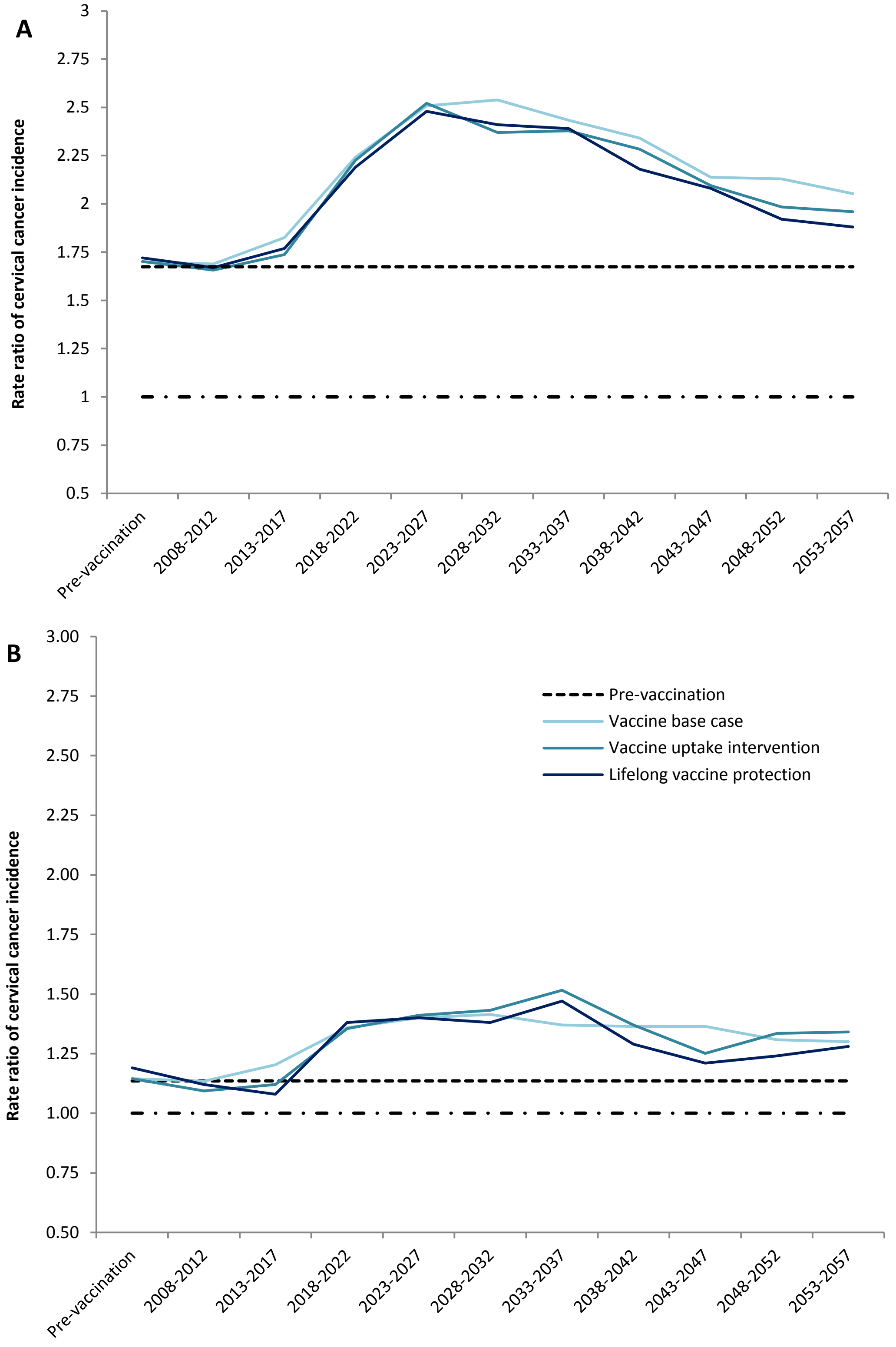

Figure S15 Sensitivity analysis assuming lifelong protection of the HPV vaccine (both Gardasil and Cervarix).

S7 Vaccination of boys

We modelled the introduction of the HPV vaccine to boys in 2008. Vaccine efficacy and duration of protection matched those of girls in the base case scenario girls (95% efficacy with protection lasting 20 years) and uptake was assumed to be equal to girls of the same ethnicity. The findings of this preliminary analysis are not conclusive and, although inequality does appear to become lower than in the base case scenario, further work is necessary to assess the effect on non-cervical cancer incidence and alternative vaccination scenarios.

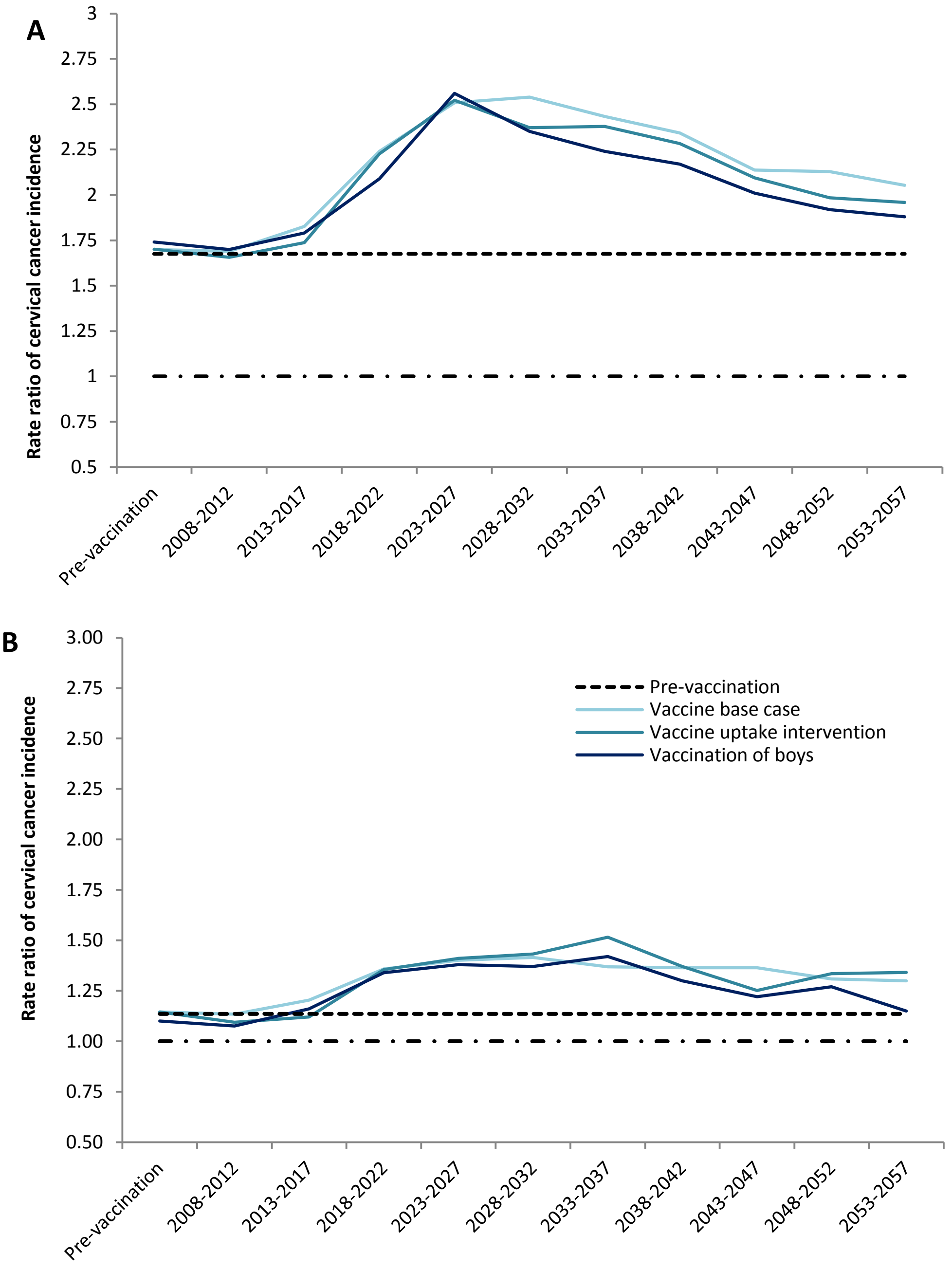

Figure S16 Preliminary exploration of the impact of vaccinating boys on rate ratios of cervical cancer incidence comparing A) Asian women with White women and B) Black women with White women. We modelled the introduction of vaccination of boys in 2018 with vaccine uptake matching that of girls of the same ethnicity. Vaccine efficacy and duration of protection matched those in girls (95% efficacy with protection lasting 20 years).

S8 Model fitting

Demographic and sexual behaviour parameters were set in the parameterisation of the model, allowing the natural history parameters for each HPV type to be fitted independently. This approach is computationally beneficial since fitting all 15 types simultaneously may have caused promising parameter sets for one type to be overshadowed by ill-fitting sets for another type. We used a sequential Monte Carlo (SMC) approach to estimate 8 parameters for each high-risk HPV type: 1) transmission probability (prior: Uniform[0,1]); 2) duration before clearance of initial infection(prior: Uniform[0,2] years); 3) duration before progression of initial infection to C(G)IN1-type lesions(prior: Uniform[0,2] years); 4) duration before spontaneous clearance of C(G)IN1-type lesions(prior: Uniform[0,5] years); 5) duration before progression to C(G)IN2-type lesions(prior: Uniform[0,5] years); 6) duration before progression to C(G)IN3-type lesions(prior: Uniform[0,5] years); 7) duration before progression to Carcinoma in Situ (prior: Uniform[0,5] years) and 8) duration before progression to cancer (either SCC or ADC) (prior: Uniform[0,200] years). We assumed that the transmission probability and duration before clearance of initial infection for these high-risk HPV types was the same for men as for women. For the low risk types, HPV-6 and -11, we estimated 2 parameters: the transmission probability and the duration before clearance of initial infection.

For each HPV type, 200 parameter sets were sampled from the joint prior distribution of the 8 natural history parameters (2 in the case of low-risk HPV). A single type HPV model was run for 50 iterations of each of these 200 sets. These single-type models were equivalent to the 15-type model in every other respect (parameterisation, model structure etc.). For each parameter set, we calculated the log-likelihood given the observed prevalence of HPV, stage 1, 2 and 3 disease and the observed incidence of Carcinoma in Situ and cervical cancer (SCC and ADC). In order to balance the contributions of each of these terms to the log-likelihood calculation, we scaled the virtual population size for each data source to 10,000.

The 200 parameter sets were weighted by their normalised log-likelihood and input parameter sets for the next round of fitting were selected by resampling them with replacement. This is equivalent to a bootstrap filter; multiplying the best-fitting parameter sets and allowing parameter sets yielding the least good fit to fade out. Each resampled parameter set was then subjected to a perturbation to allow for the evaluation of new areas of parameter space. The perturbation was a Gaussian walk, with mean in each dimension equal to the previous parameter value and standard deviation equal to half the standard deviation of all sampled values in that same dimension. Where the perturbation was large, or where particles originated close to the bounds of parameter space, it was possible that a particle may be perturbed outside the bounds of the prior. In this case, we chose to reflect particles off the edges of parameter space. Thus particles which are perturbed far outside the prior bounds are used to explore the prior range in a non-informed way. Particles which are perturbed just outside the prior bounds are used to explore the regions close to the boundaries. As the variance in the perturbed sets decreases, fewer particles are perturbed outside the prior bounds and the posterior takes shape. The single-type HPV model was then run for a further 50 iterations of these new parameter sets.

This sequential approach was reiterated until it was deemed, by eye, that the model output gave an acceptable fit to the observed prevalence of HPV infection and cervical disease and incidence of CiS and cervical cancer. In practice, this was 4 rounds for each HPV type. Figure S17 shows the fit of the model output to data on the age-specific HPV prevalence and incidence cervical disease by stage for each HPV type modelled. Figures S20-S27 show the posterior distributions for the 8 estimated parameters for each of the 13 high-risk types modelled. Figure S18 shows the estimated HPV prevalence in males, although the model was not fitted to data on this outcome.

The rates of progression from HPV infection to non-cervical cancer (NCC) were fitted in a similar way. We modelled anal, oropharyngeal and laryngeal cancer in both men and women and vulval/vaginal and penile cancers. For each NCC, the proportion of HPV infections progressing to NCC was estimated (prior: Uniform[0,0.01]), as were the shape (prior: Uniform[0,50]) and scale (prior: Uniform[0,50]) parameters for a Gamma distribution. The product of the shape and scale gave the time lag before cancer

diagnosis. Figure S19 shows the fit of the model output to data on the age-specific incidence of non-cervical cancer. Table S8 gives the posterior distributions of the estimated parameter values for each non-cervical cancer.

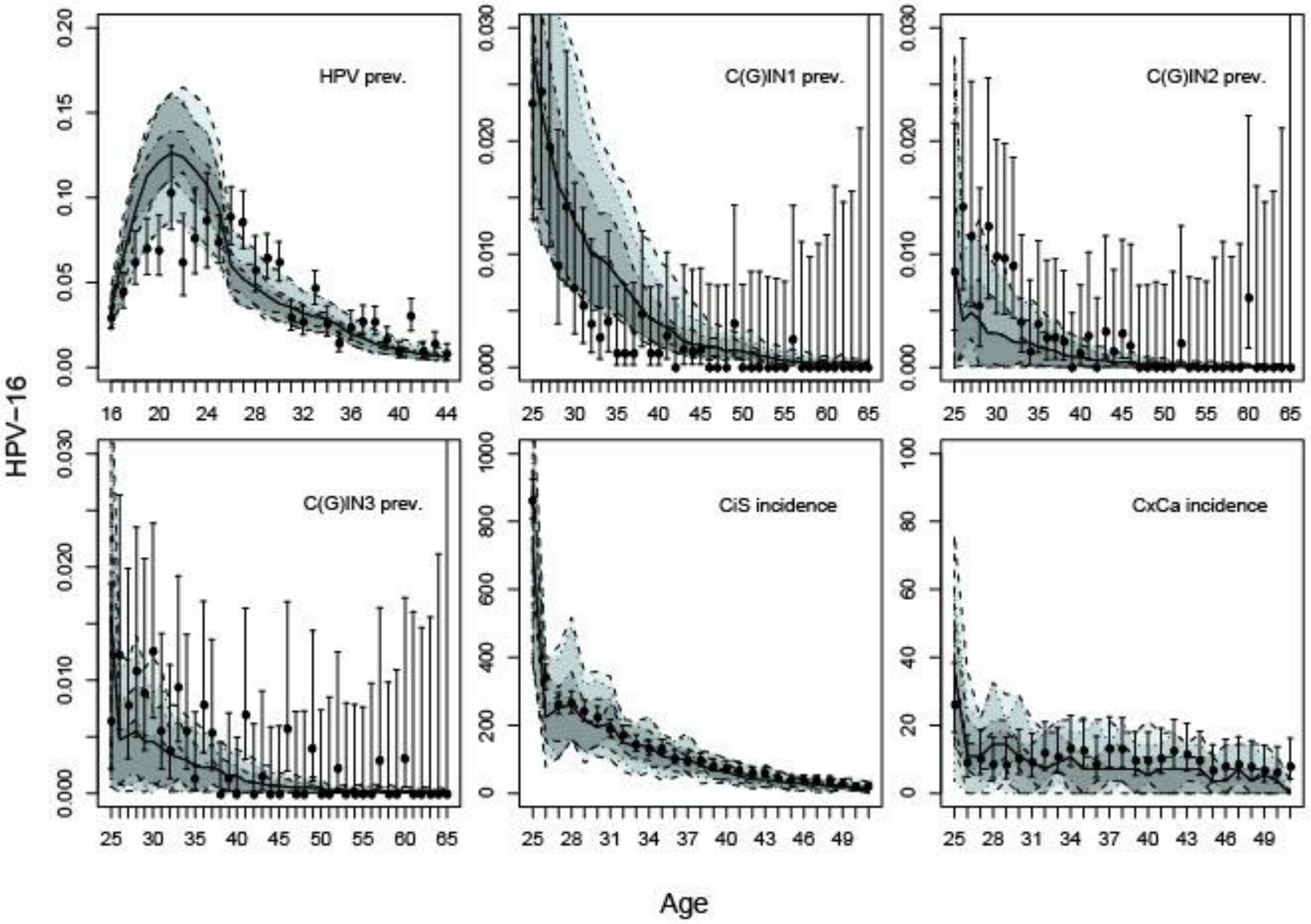

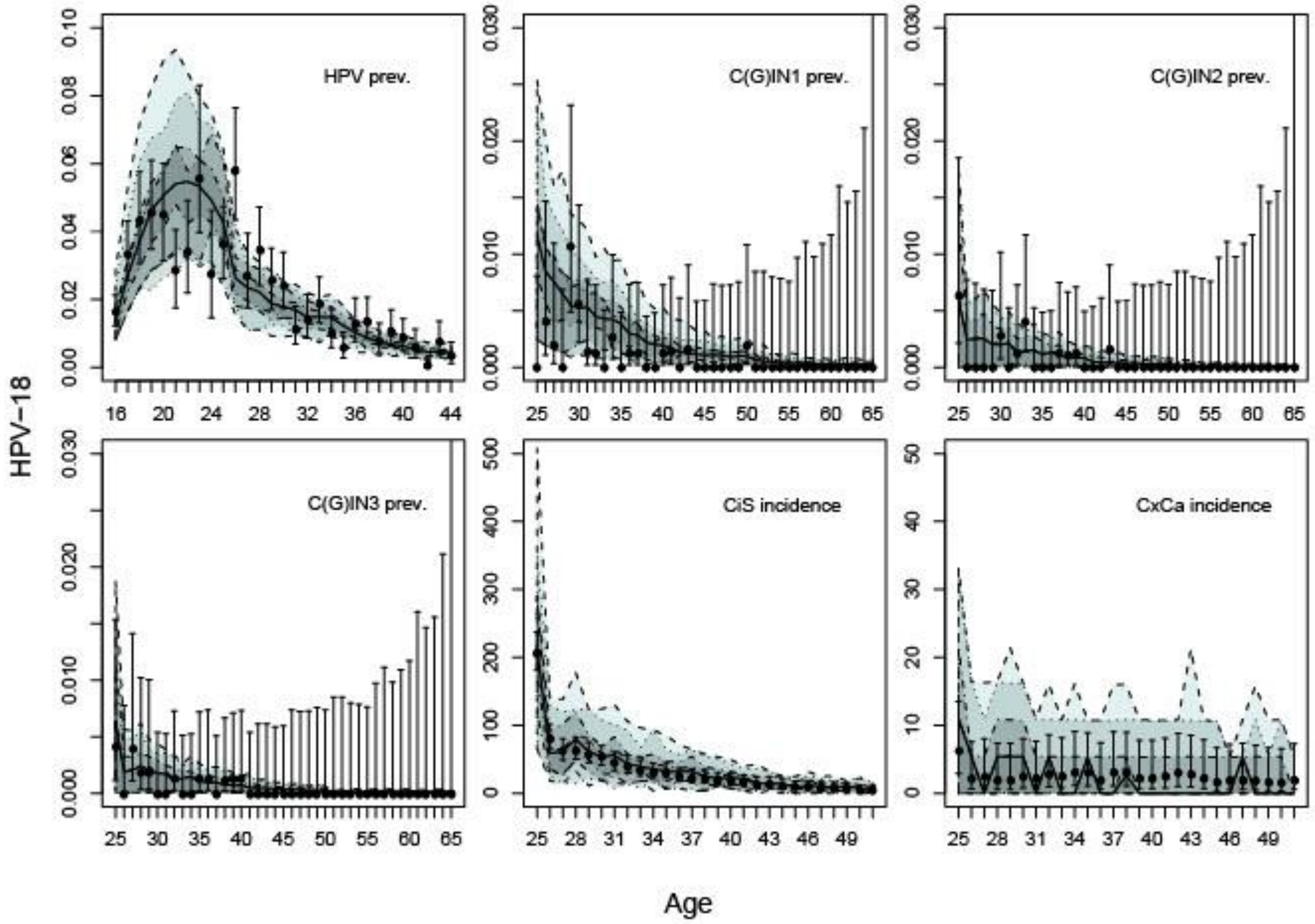
HPV−18
HPV prev.
C(G)IN1 prev.
C(G)IN2 prev.
C(G)IN3 prev.
CiS incidence
CxCa incidence
Age

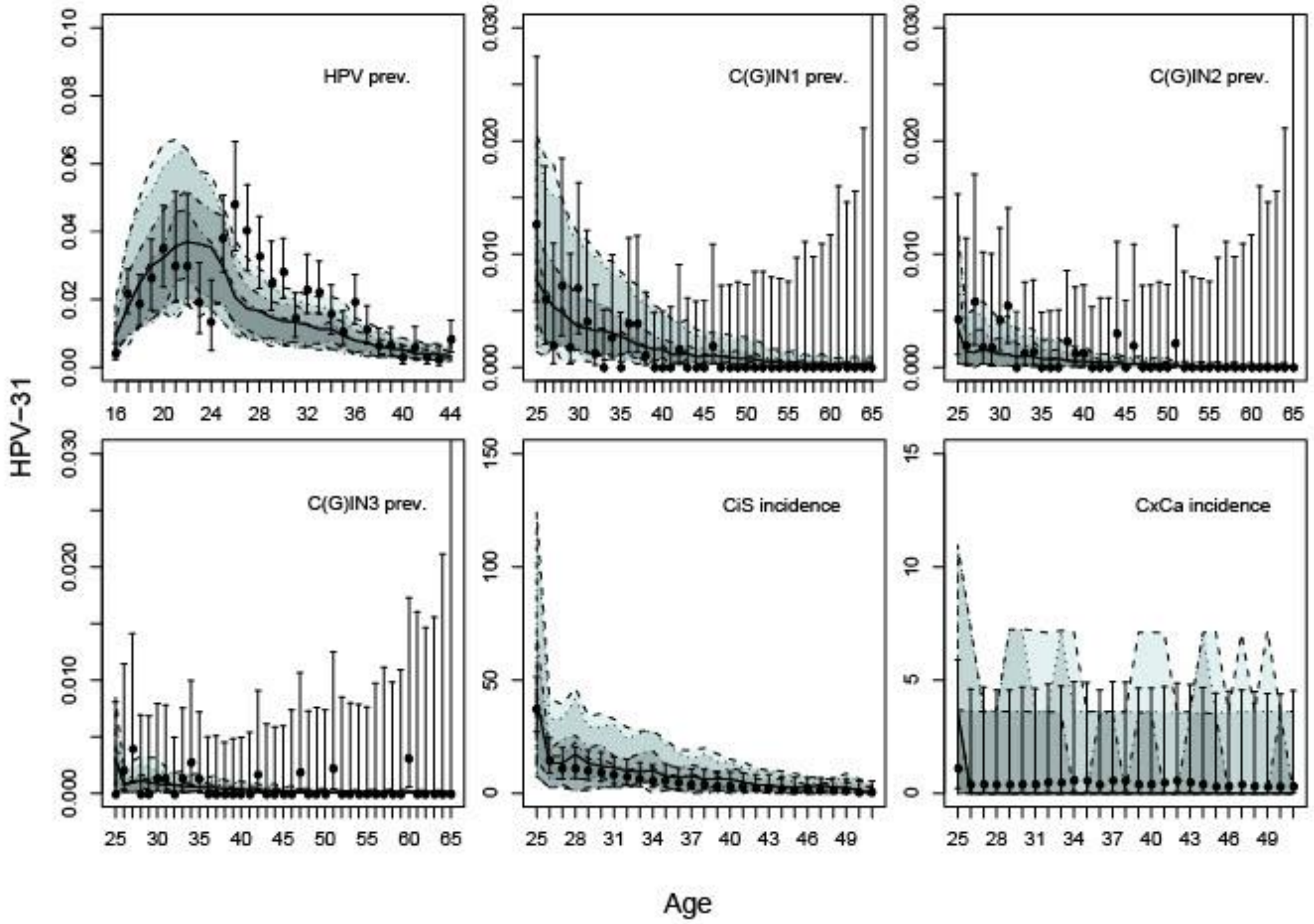
HPV−31
HPV prev.
C(G)IN1 prev.
C(G)IN2 prev.
C(G)IN3 prev.
CiS incidence
CxCa incidence
Age

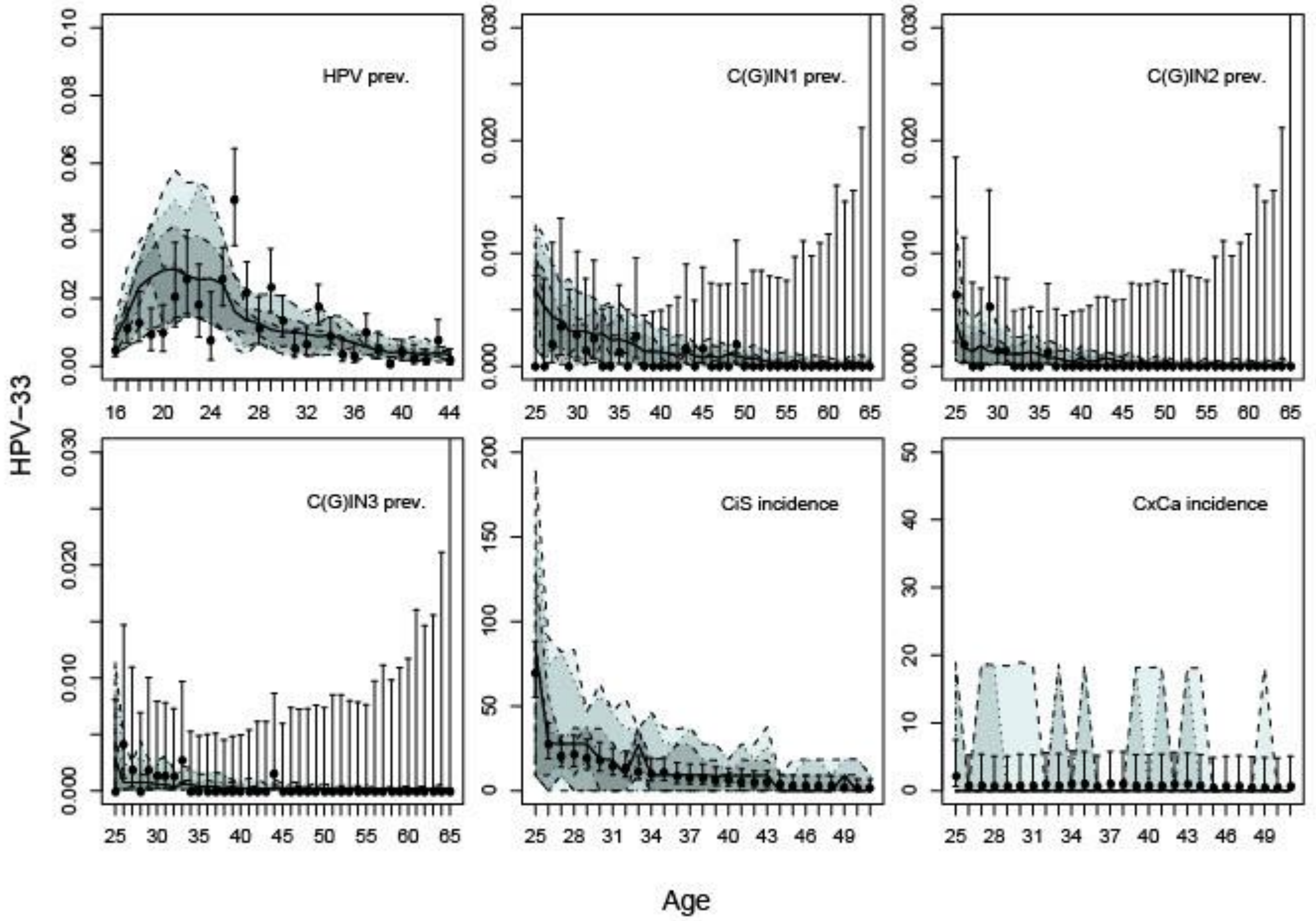
HPV prev.
C(G)IN1 prev.
C(G)IN2 prev.
C(G)IN3 prev.
CiS incidence
CxCa incidence
HPV−33
Age

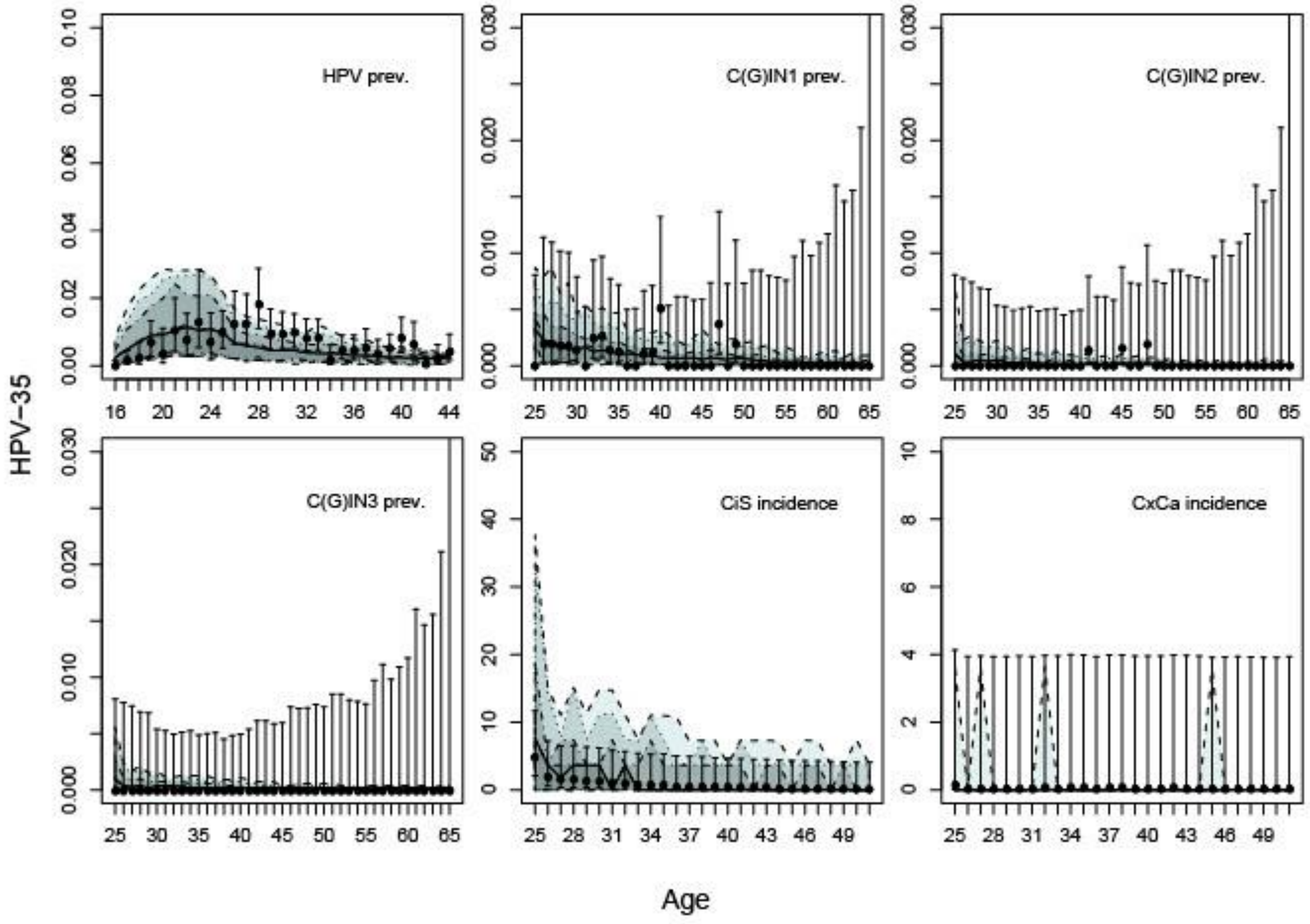
HPV-35
HPV prev.
C(G)IN1 prev.
C(G)IN2 prev.
C(G)IN3 prev.
CiS incidence
CxCa incidence
Age

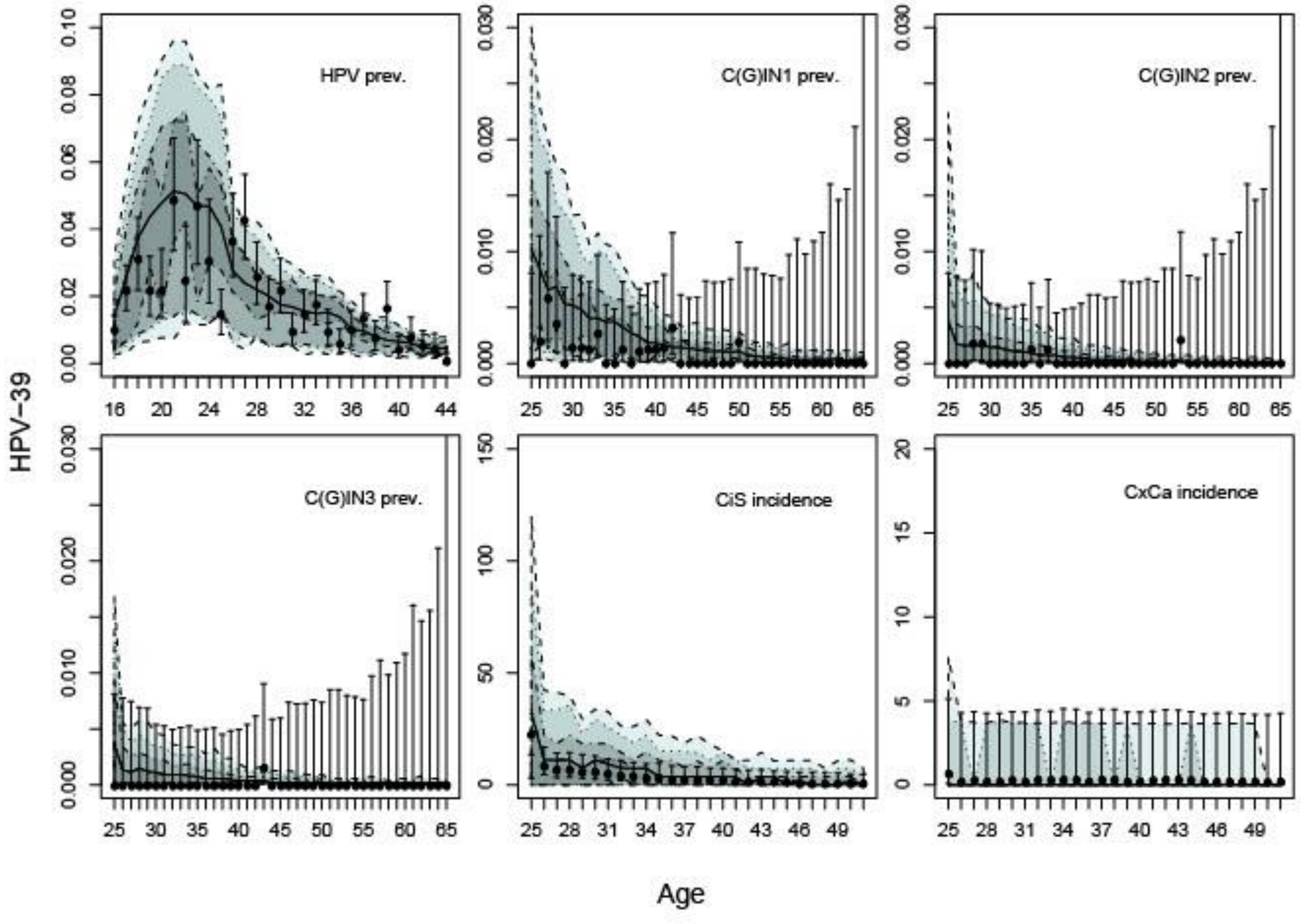
HPV-39
HPV prev.
C(G)IN1 prev.
C(G)IN2 prev.
C(G)IN3 prev.
CiS incidence
CxCa incidence
Age

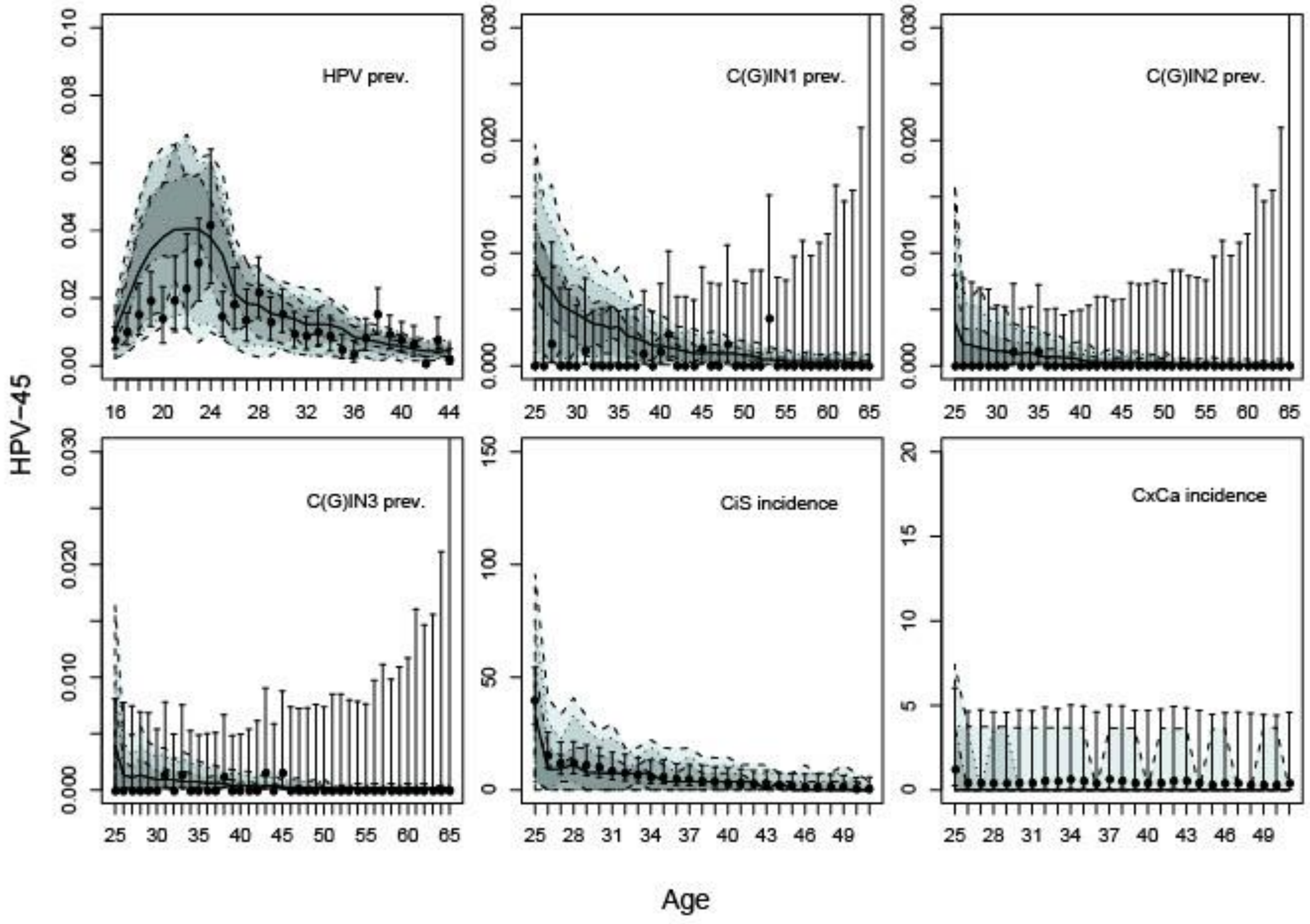
HPV−45
HPV prev.
C(G)IN1 prev.
C(G)IN2 prev.
C(G)IN3 prev.
CiS incidence
CxCa incidence
Age

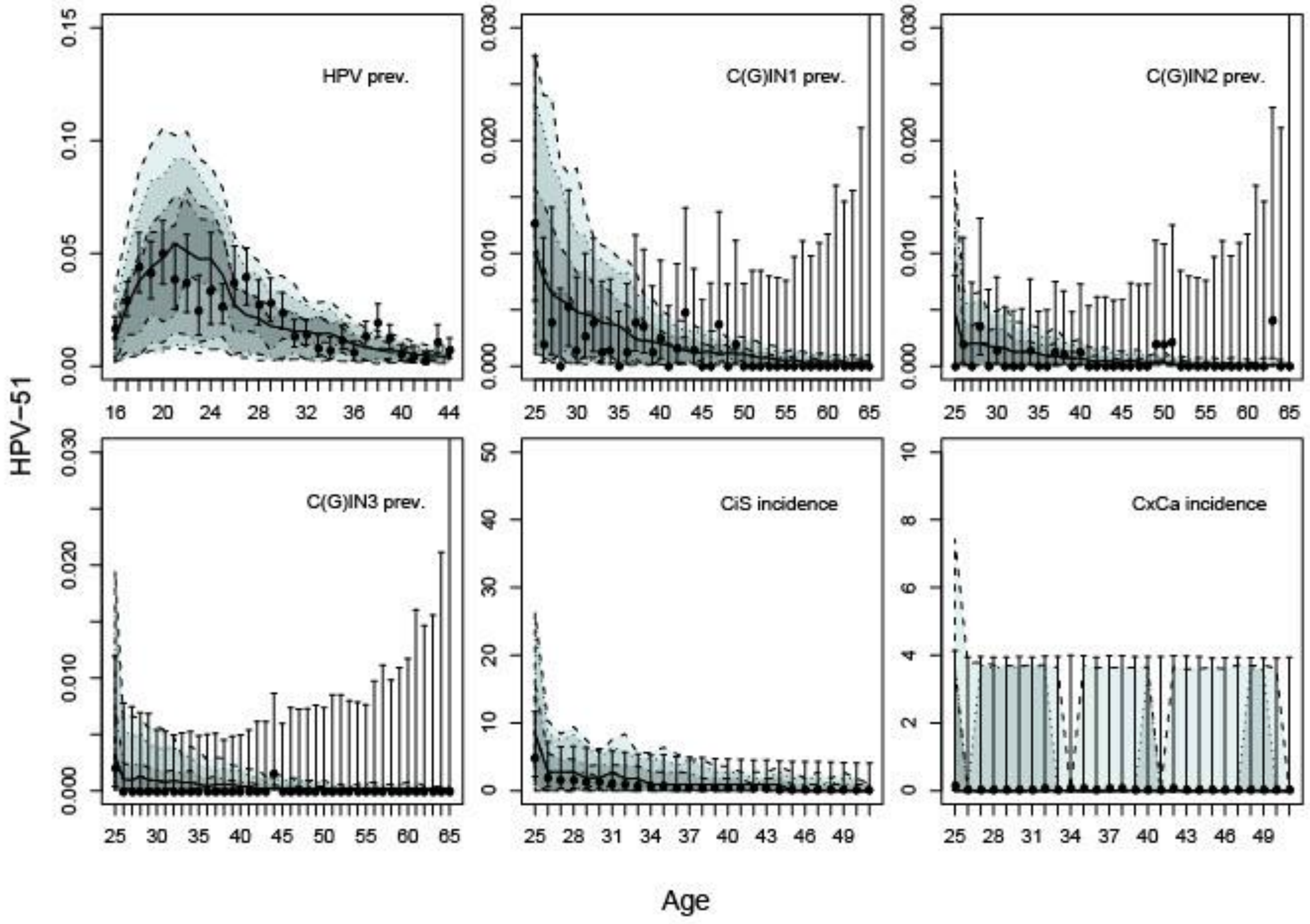
HPV−51
HPV prev.
C(G)IN1 prev.
C(G)IN2 prev.
C(G)IN3 prev.
CiS incidence
CxCa incidence
Age

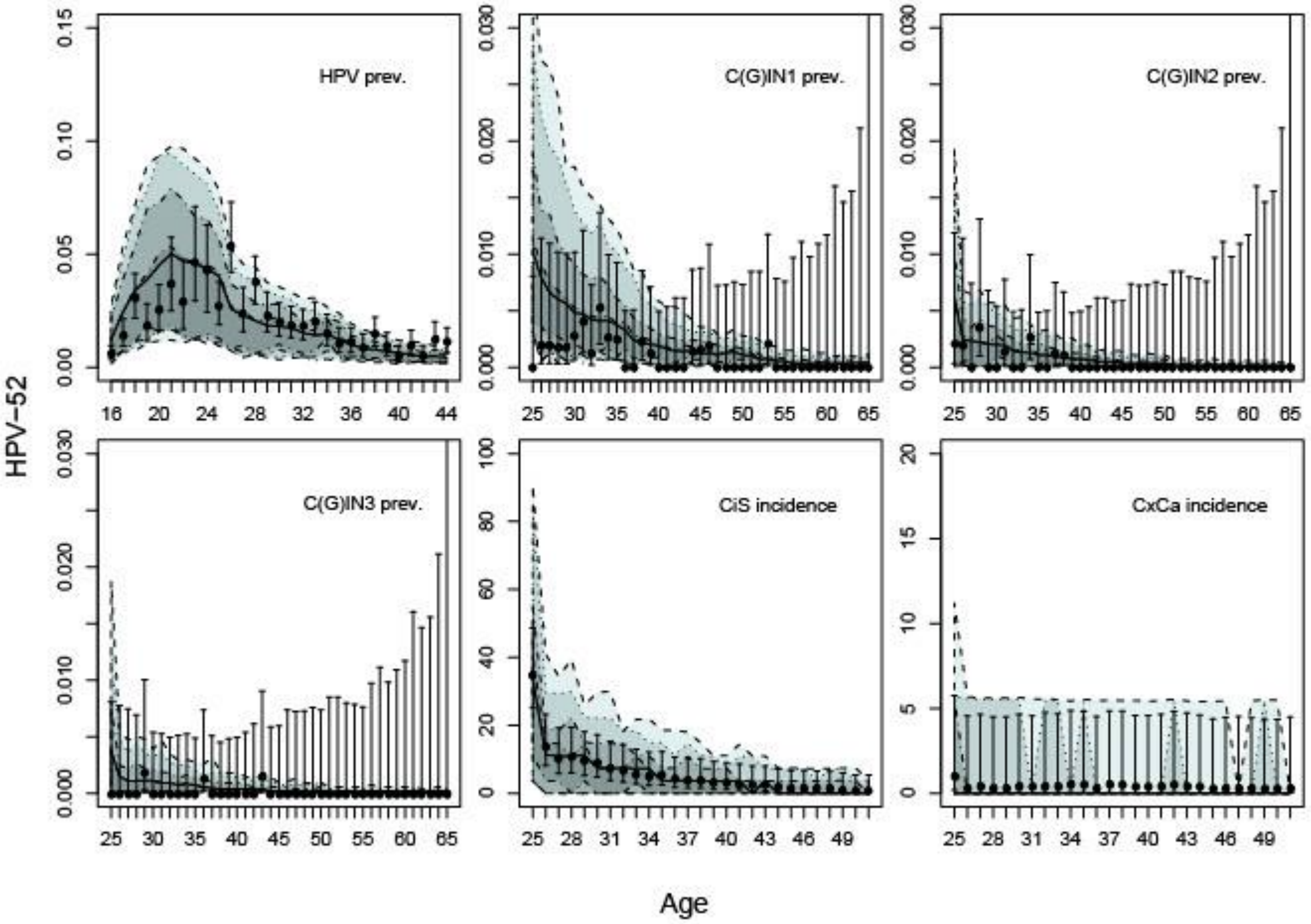
HPV−52
HPV prev.
C(G)IN1 prev.
C(G)IN2 prev.
C(G)IN3 prev.
CiS incidence
CxCa incidence
Age

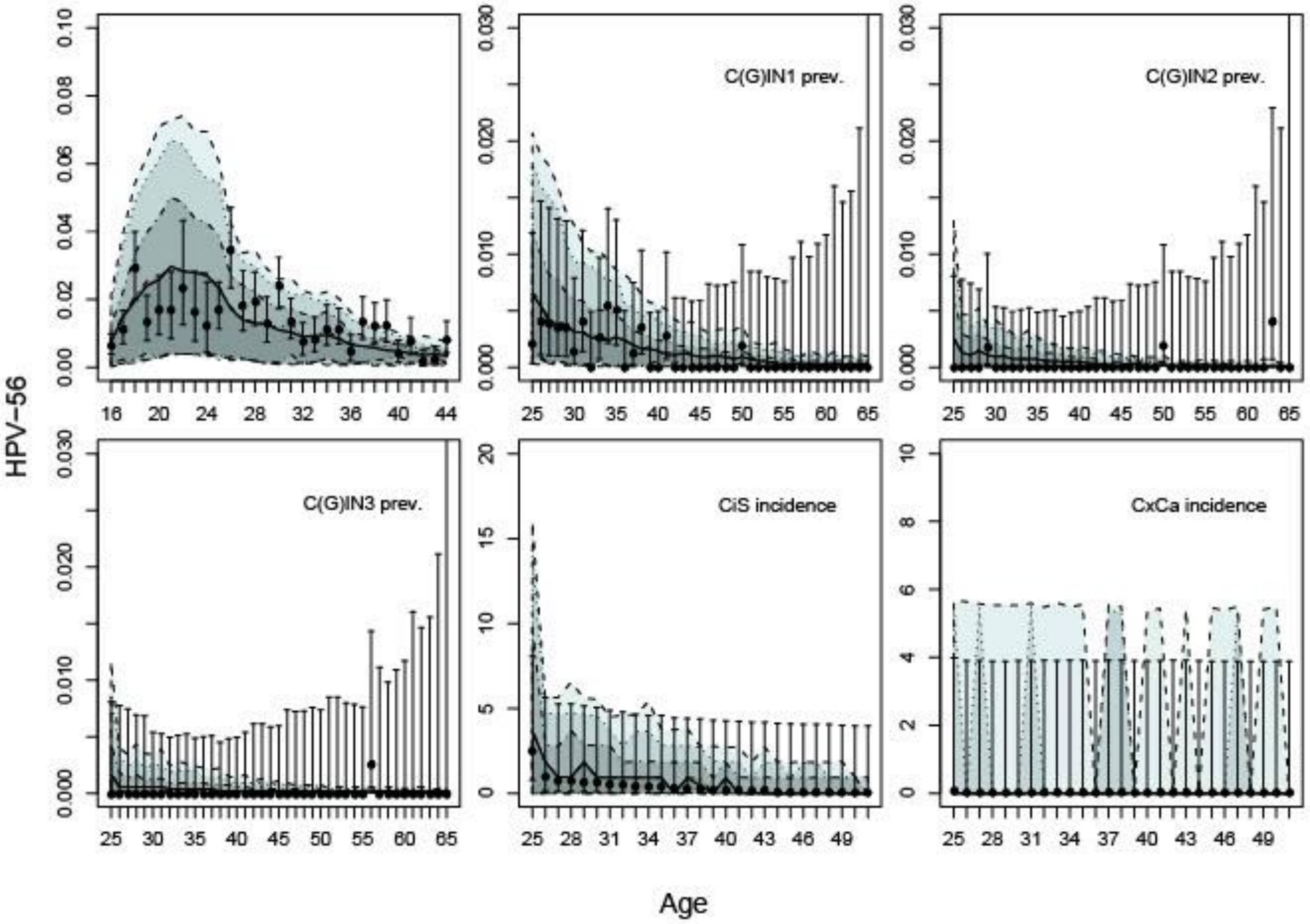
HPV−56
C(G)IN1 prev.
C(G)IN2 prev.
C(G)IN3 prev.
CiS incidence
CxCa incidence
Age

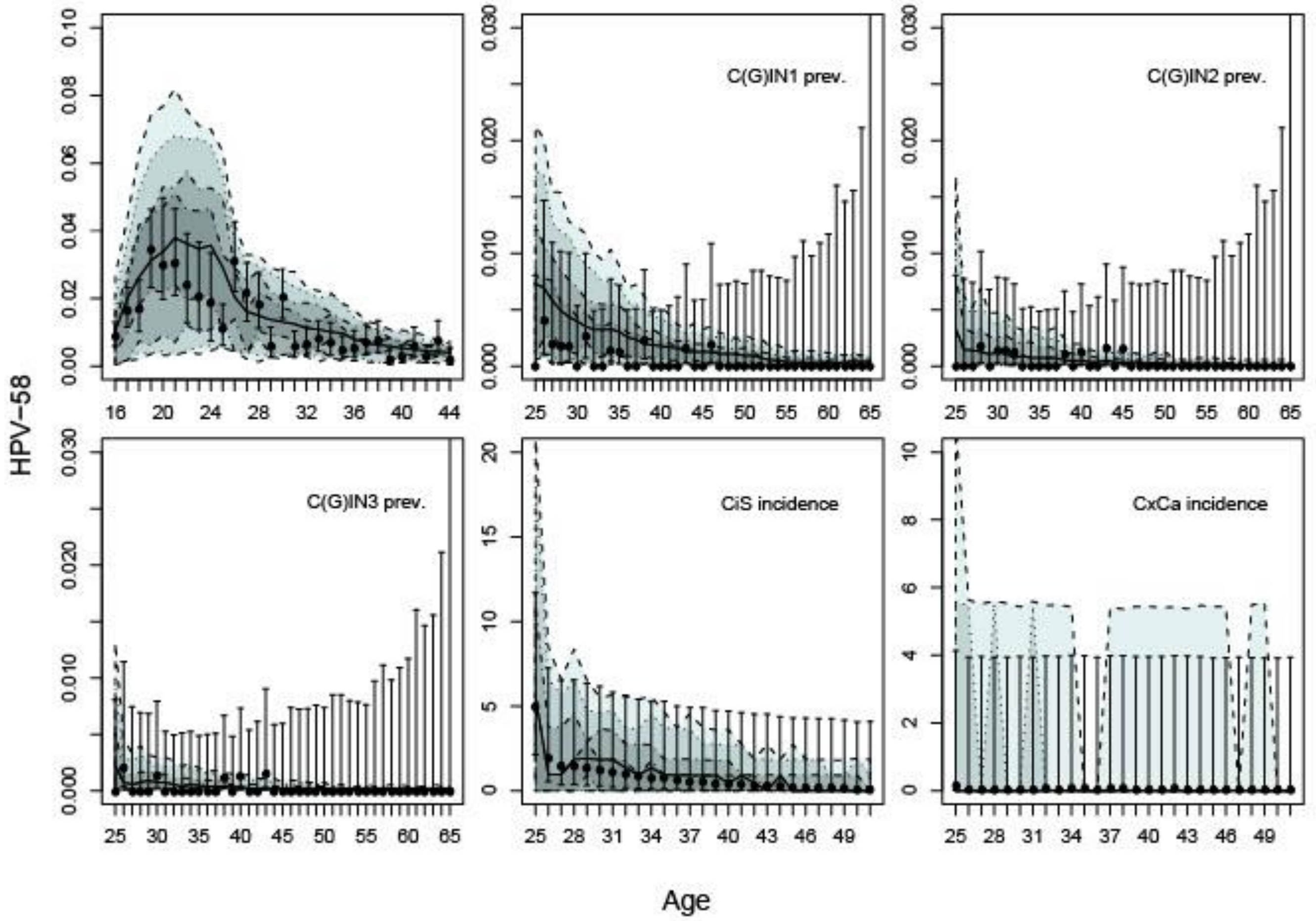
HPV−58
C(G)IN1 prev.
C(G)IN2 prev.
C(G)IN3 prev.
CiS incidence
CxCa incidence
Age

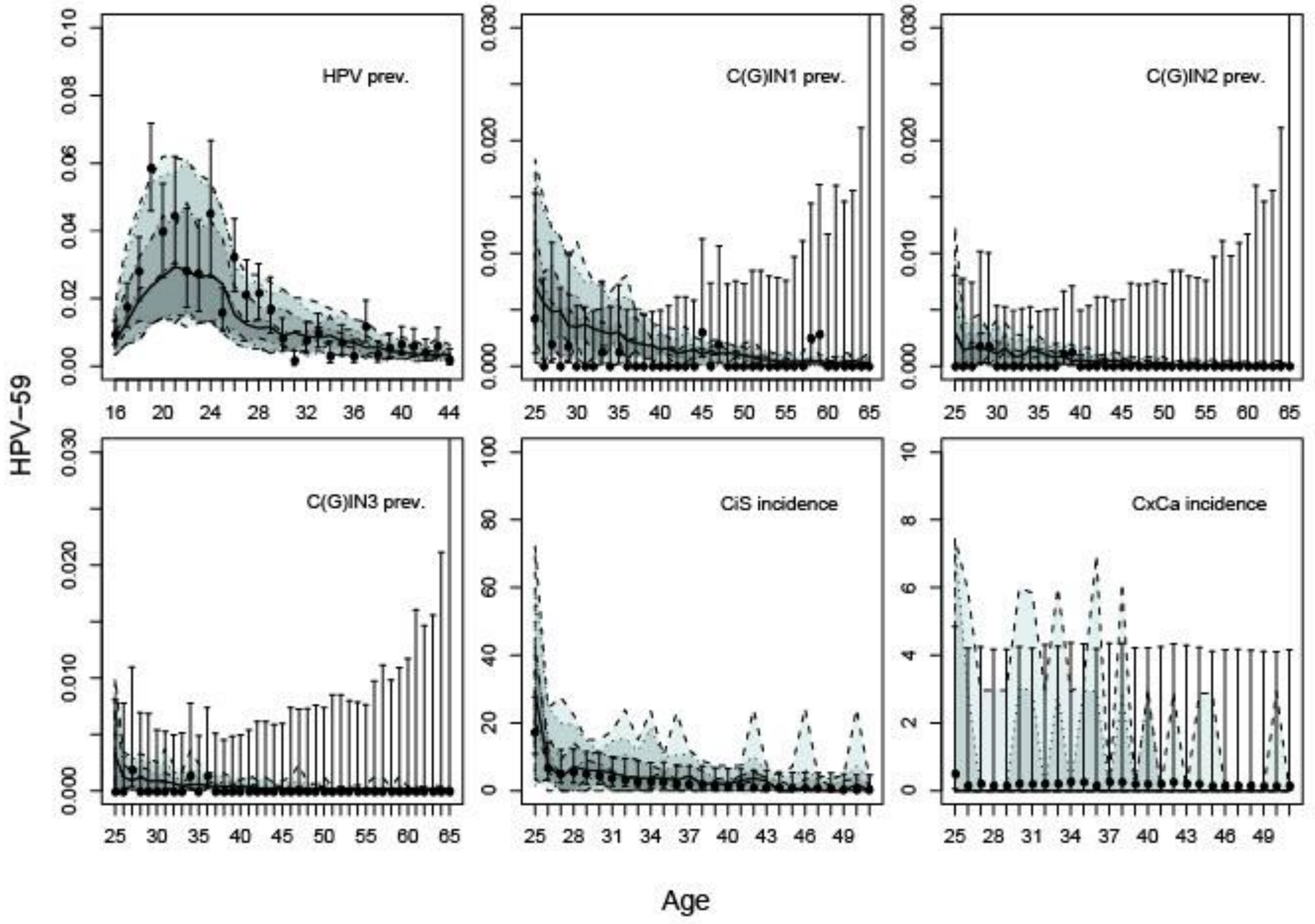
HPV−59
HPV prev.
C(G)IN1 prev.
C(G)IN2 prev.
C(G)IN3 prev.
CiS incidence
CxCa incidence
Age

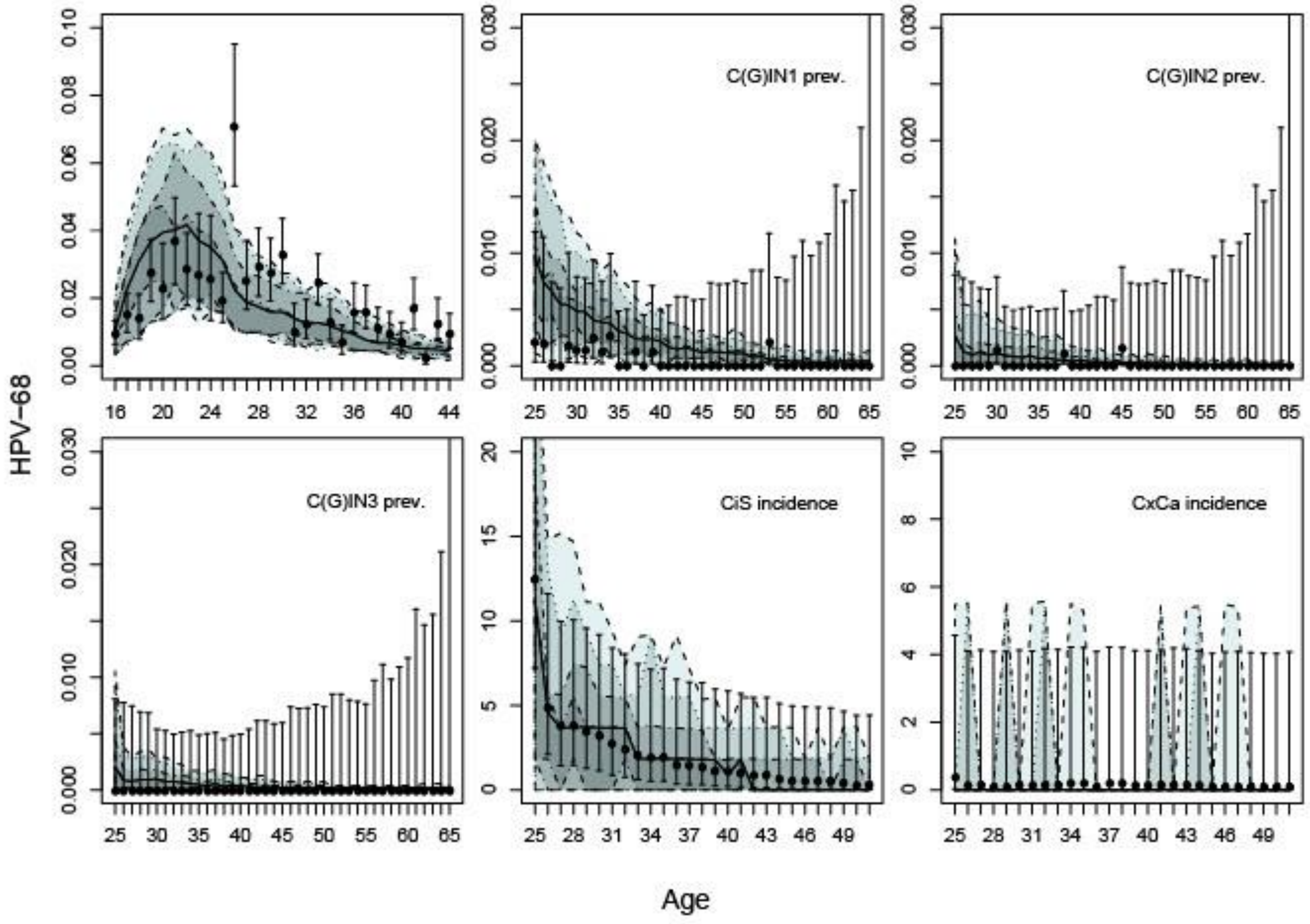

Figure S17 **Model fit to HPV infection prevalence in females and cervical disease by age and stage for each of the 13 high-risk types. The median predicted prevalence for each type is represented by the solid curve and the turquoise bands represent the 50%, 70%, 90% and 95% posterior intervals.**

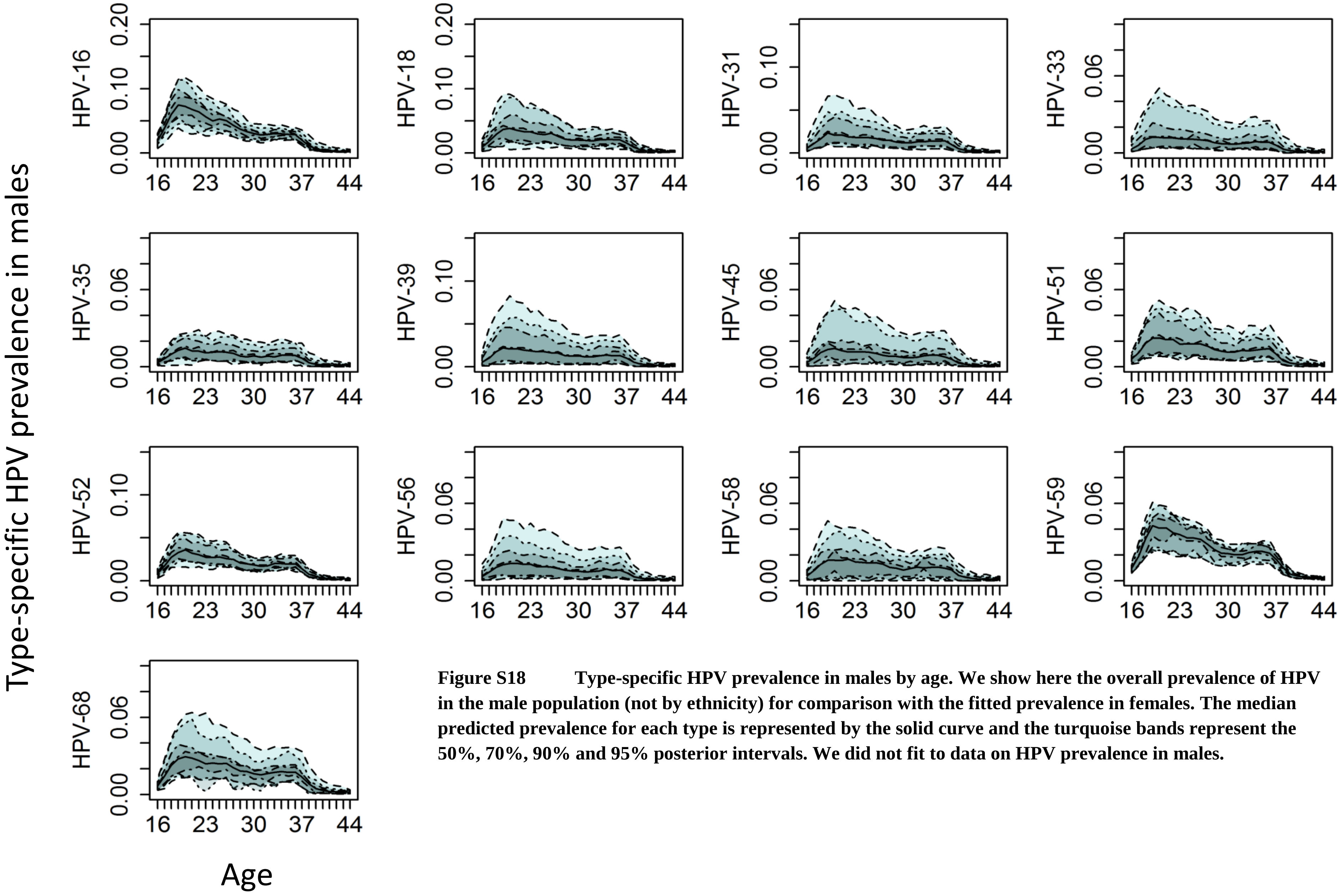

Figure S18 **Type-specific HPV prevalence in males by age. We show here the overall prevalence of HPV in the male population (not by ethnicity) for comparison with the fitted prevalence in females. The median predicted prevalence for each type is represented by the solid curve and the turquoise bands represent the 50%, 70%, 90% and 95% posterior intervals. We did not fit to data on HPV prevalence in males.**

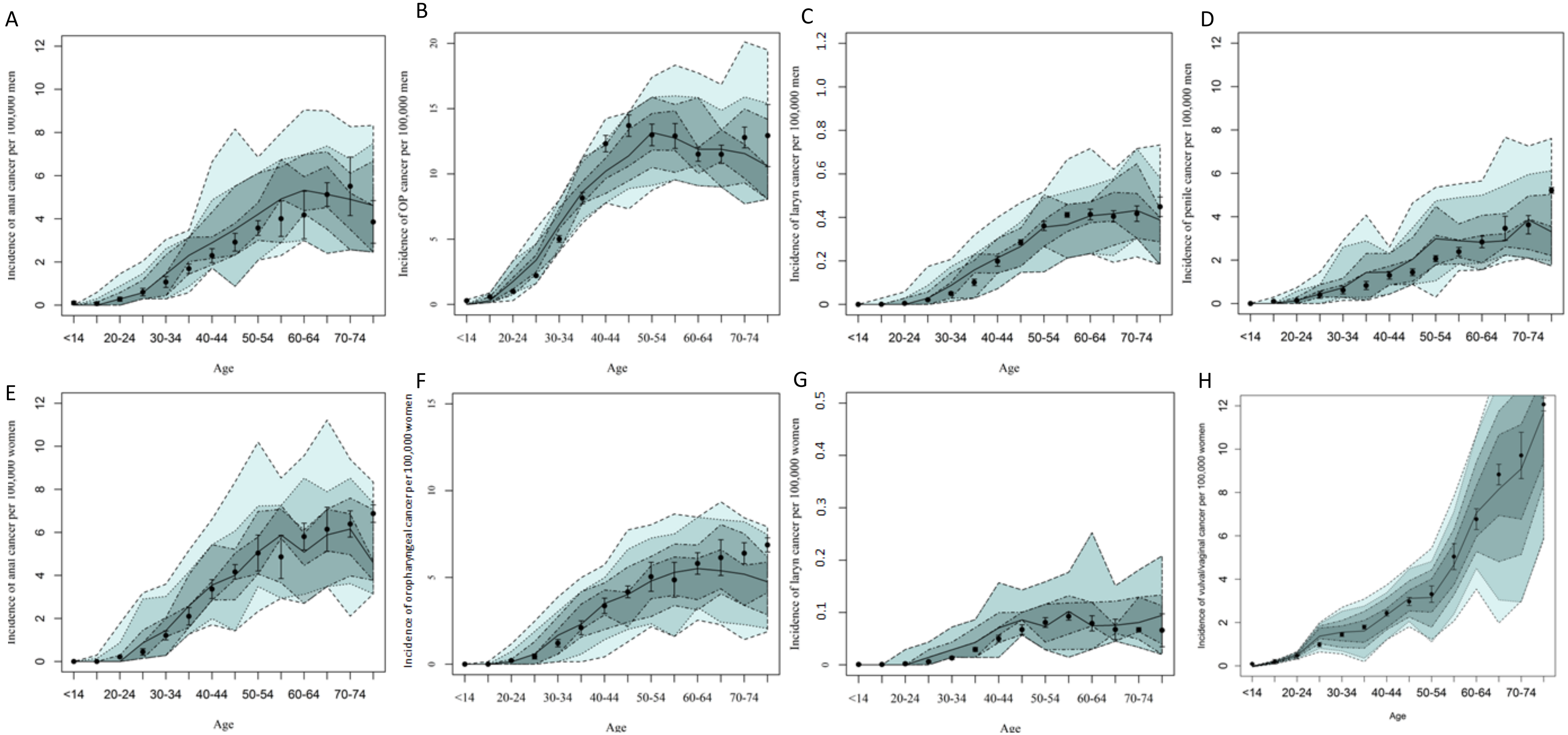

Figure S19 **Model fit of non-cervical cancer incidence to data by age (ONS). A) Incidence of anal cancer per 100,000 men; B) incidence of oropharyngeal cancer per 100,000 men; C) incidence of laryngeal cancer per 100,000 men; D) incidence of penile cancer per 100,000 men; E) incidence of anal cancer per 100,000 women; F) incidence of oropharyngeal cancer per 100,000 women; G) incidence of laryngeal cancer per 100,000 women; H) incidence of vulval and vaginal cancer per 100,000 women. The median predicted prevalence for each type is represented by the solid curve and the turquoise bands represent the 50%, 70%, 90% and 95% posterior intervals.**

	Median (95% posterior interval)			
Cancer type	Proportion of HPV infections progressing to cancer type	Shape of Gamma distribution (years)	Scale of Gamma distribution (years)	Average time lag before developing cancer (product)
Anal (female)	0.00414 (0.00346 – 0.00500)	4.17 (3.27 – 5.76)	14.3 (8.38 – 19.8)	59.6 years
Oropharyngeal (female)	0.00413 (0.00327 – 0.00503)	4.07 (3.04 – 6.02)	13.5 (7.45 – 20.1)	54.8 years
Laryngeal (female)	0.000756 (0.000480 – 0.00197)	5.82 (1.88 – 13.6)	8.97 (4.37-31.2)	52.2 years
Vulval / vaginal	0.0474 (0.0352 – 0.0523)	15.8 (10.2 – 24.6)	83.5 (50.4 – 107)	1320 years
Anal (male)	0.00357 (0.00176 – 0.00523)	2.27 (1.93 – 4.20)	34.2 (10.2 -48.5)	77.8 years
Oropharyngeal (male)	0.00809 (0.00537 – 0.0102)	2.11 (1.86-3.02)	28.5 (14.8 -36.2)	60.0 years
Laryngeal (male)	0.00284 (0.00194 – 0.00349)	3.12 (2.78-5.06)	21.4 (8.61-28.8)	66.9 years
Penile	0.00235 (0.00157 – 0.00385)	2.90 (1.93-3.86)	29.4 (19.5-46.5)	85.3 years

Table S8 Posterior ranges for the proportion of HPV infections progressing to non-cervical cancers and the estimated values for the shape and scale parameters of the Gamma distribution describing the time lag. For ease of comparison, we include also the estimated average time lag for each cancer, calculated from the posterior parameters of the Gamma distribution. For each non-cervical cancer, the prior range for the proportion of HPV infections progressing was Uniform[0,0.01] and the prior distribution for the shape and scale parameters were both Uniform[0, 50years]

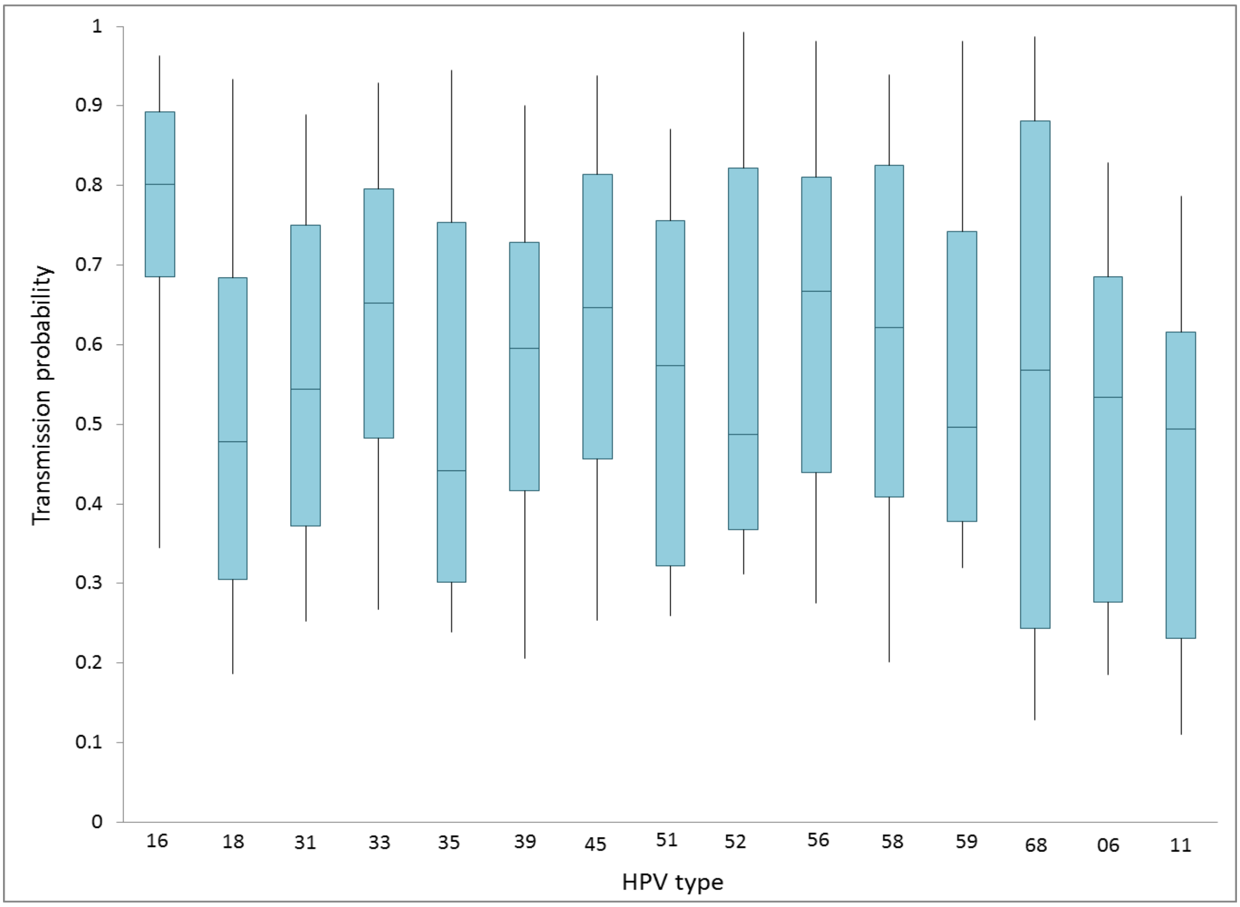

Figure S20 Posterior distributions of transmission probability for each of the 13 high-risk and 2 low-risk HPV types modelled. Box plots show the median value and interquartile range (25th and 75th percentiles), whiskers show the 5th and 95th percentiles. The prior distribution was Uniform[0,1].

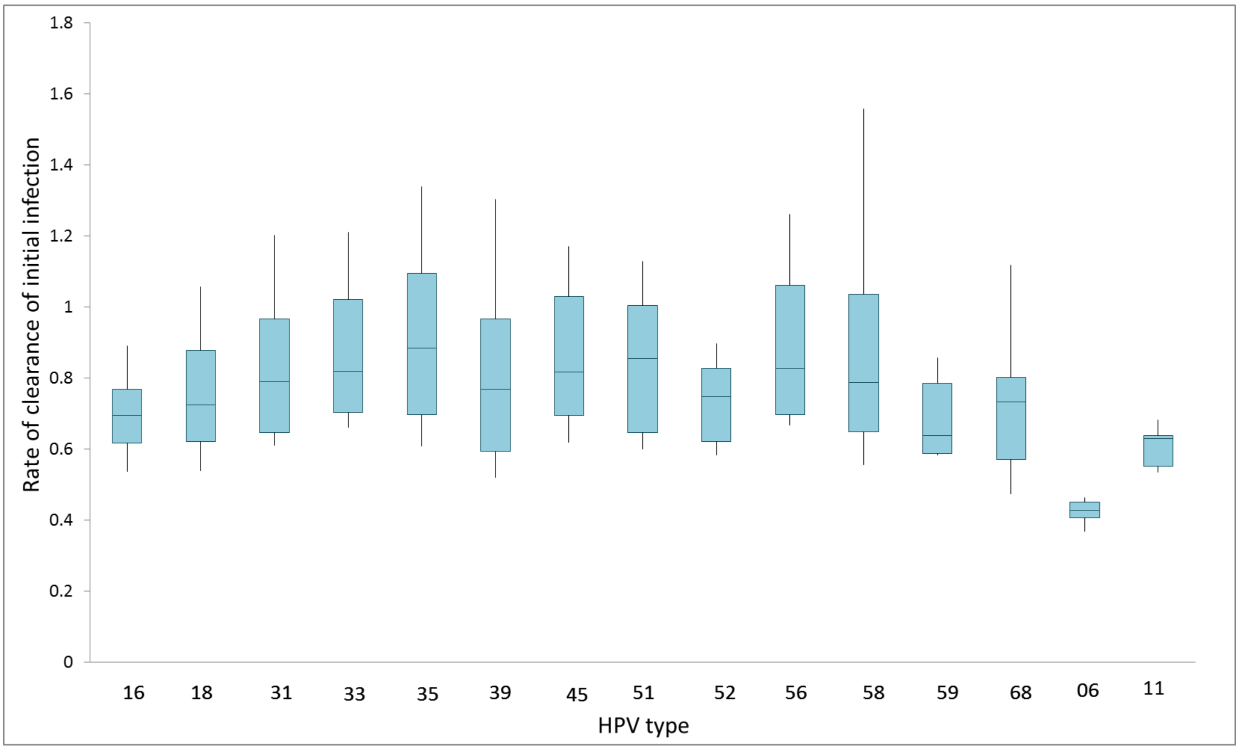

Figure S21 Posterior distributions of the rate of clearance of initial infection for each of the 13 high-risk and 2 low-risk HPV types modelled. Box plots show the median value and interquartile range (25th and 75th percentiles), whiskers show the 5th and 95th percentiles. The prior distribution was for duration before clearance and was Uniform[0,2] years.

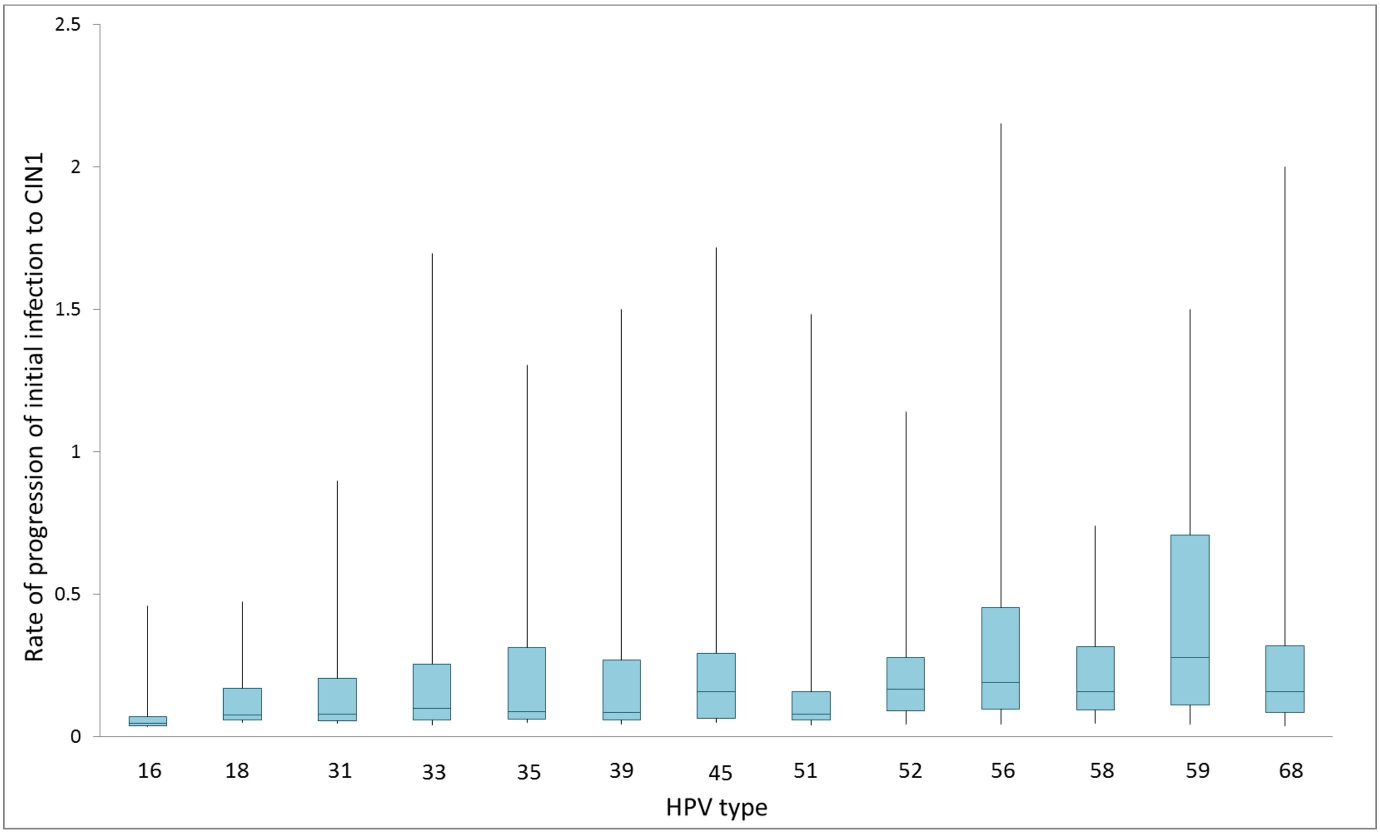

Figure S22 Posterior distributions of the rate of progression of initial infection to CIN1 for each of the 13 high-risk HPV types modelled. Box plots show the median value and interquartile range (25th and 75th percentiles), whiskers show the 5th and 95th percentiles. The prior distribution was for duration before progression and was Uniform[0,2] years.

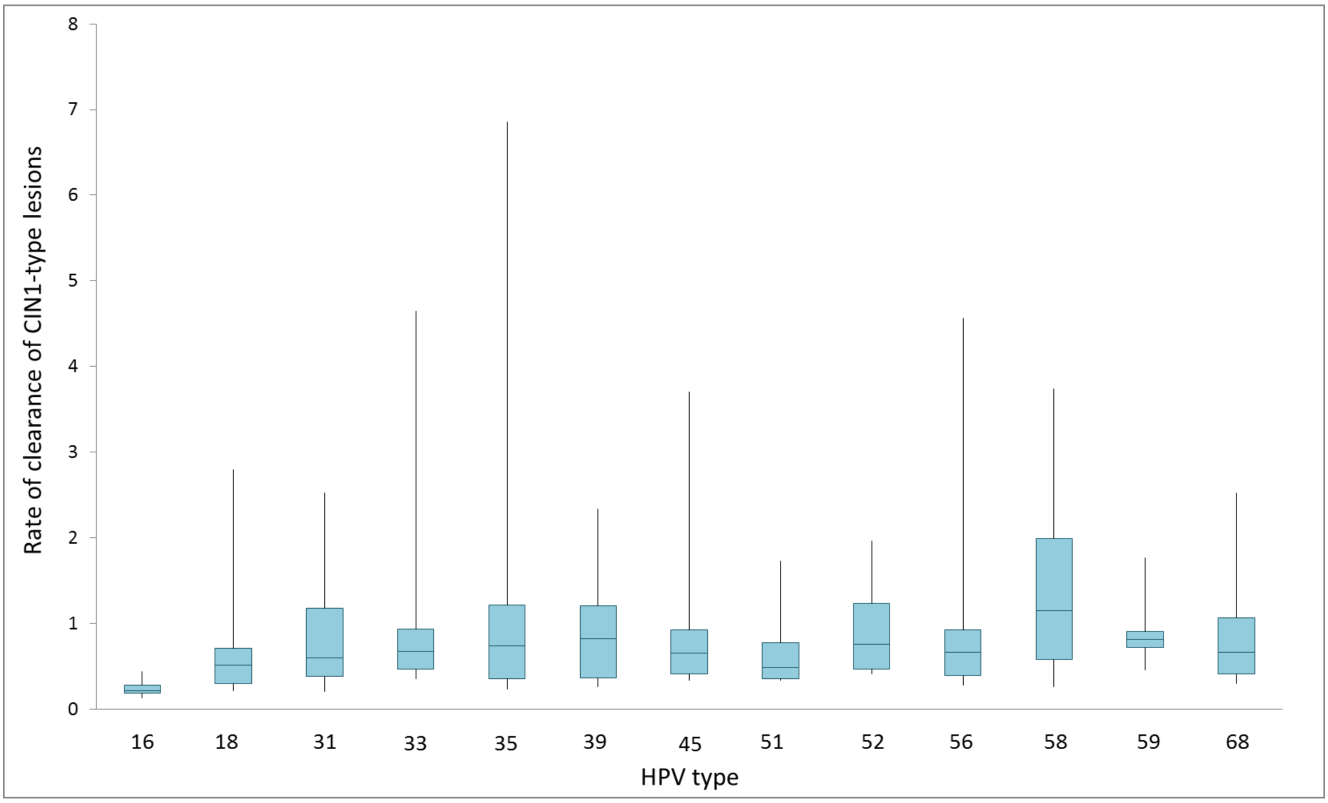

Figure S23 Posterior distributions of the rate of clearance of CIN1-type lesions for each of the 13 high-risk HPV types modelled. Box plots show the median value and interquartile range (25th and 75th percentiles), whiskers show the 5th and 95th percentiles. The prior distribution was for duration before progression and was Uniform[0,5] years.

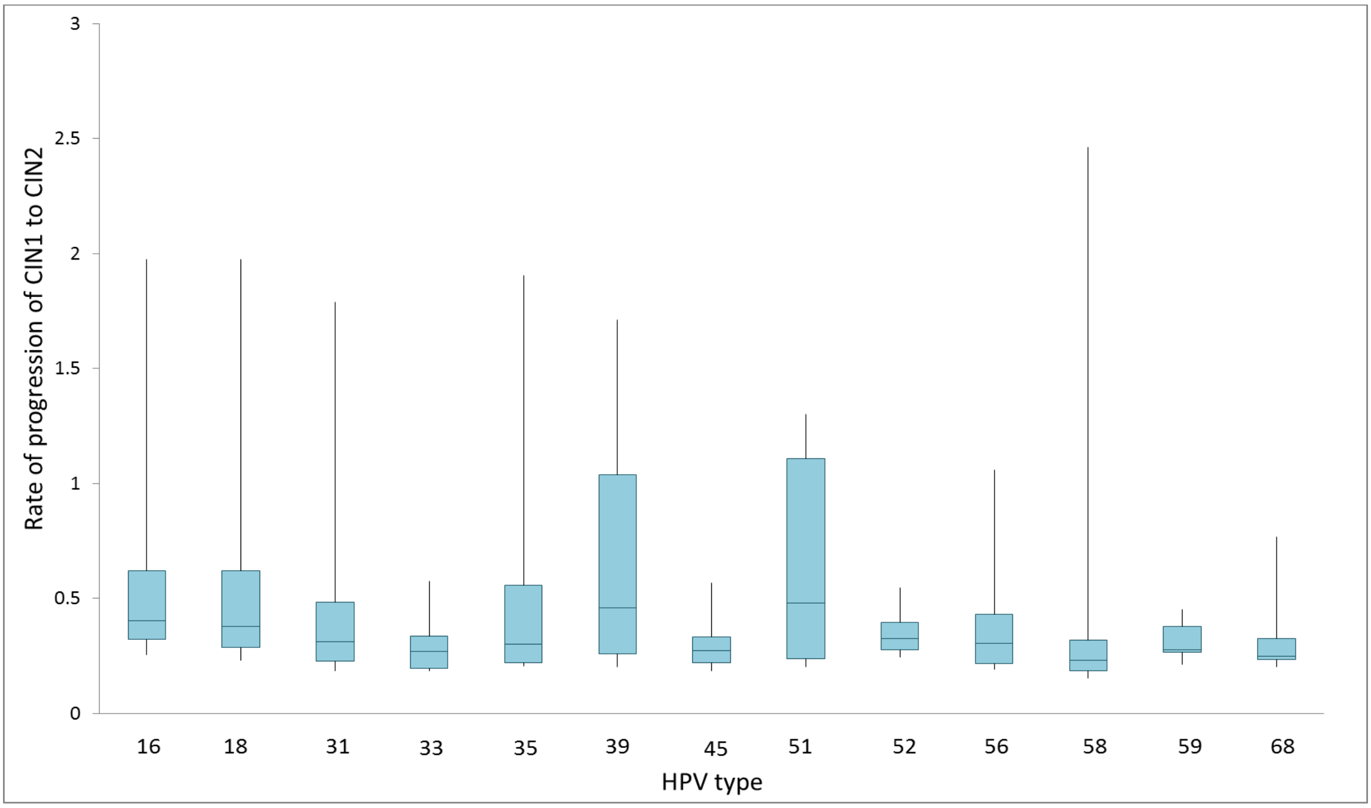

Figure S24 Posterior distributions of the rate of progression of CIN1 to CIN2 for each of the 13 high-risk HPV types modelled. Box plots show the median value and interquartile range (25th and 75th percentiles), whiskers show the 5th and 95th percentiles. The prior distribution was for duration before progression and was Uniform[0,5] years.

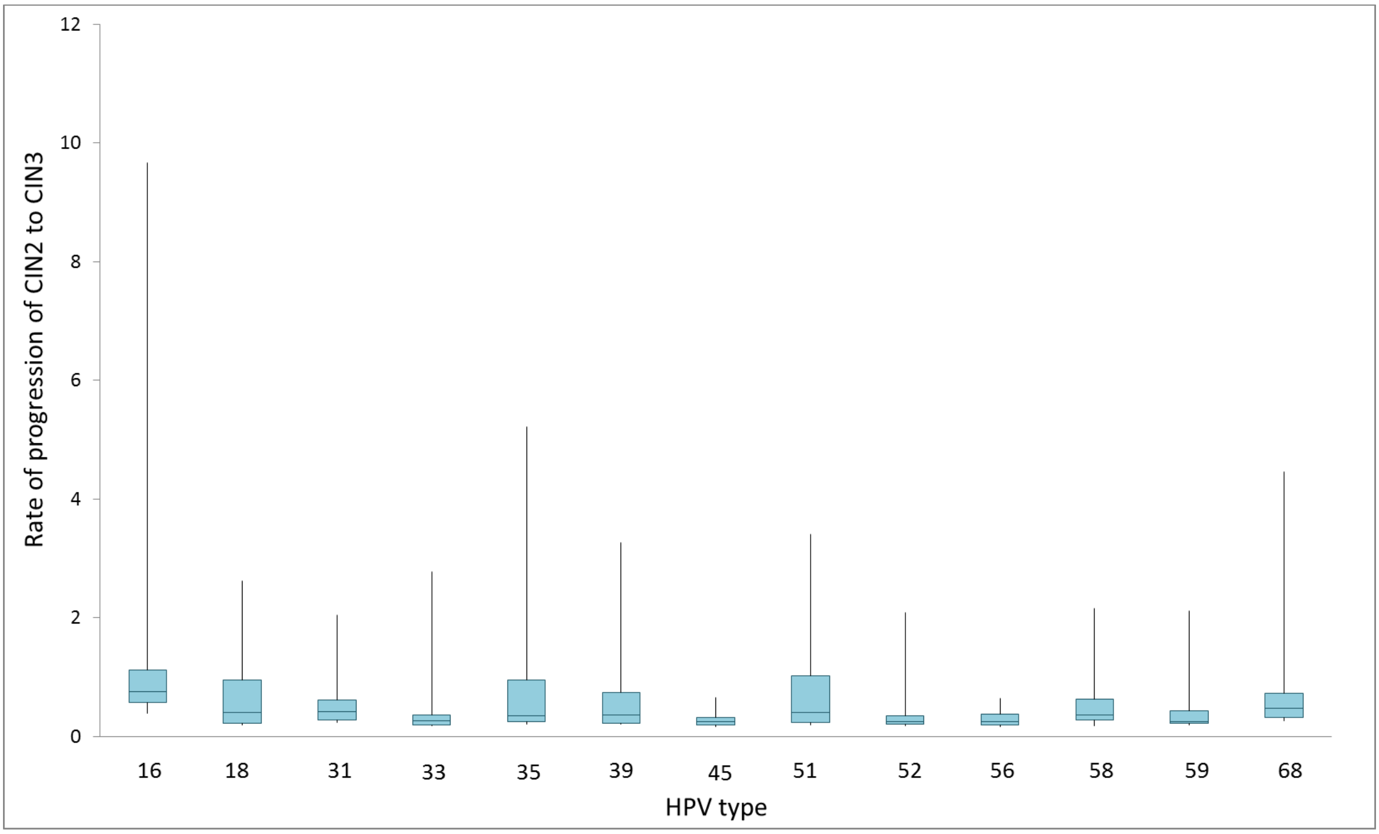

Figure S25 Posterior distributions of the rate of progression of CIN2 to CIN3 for each of the 13 high-risk HPV types modelled. Box plots show the median value and interquartile range (25th and 75th percentiles), whiskers show the 5th and 95th percentiles. The prior distribution was for duration before progression and was Uniform[0,5] years.

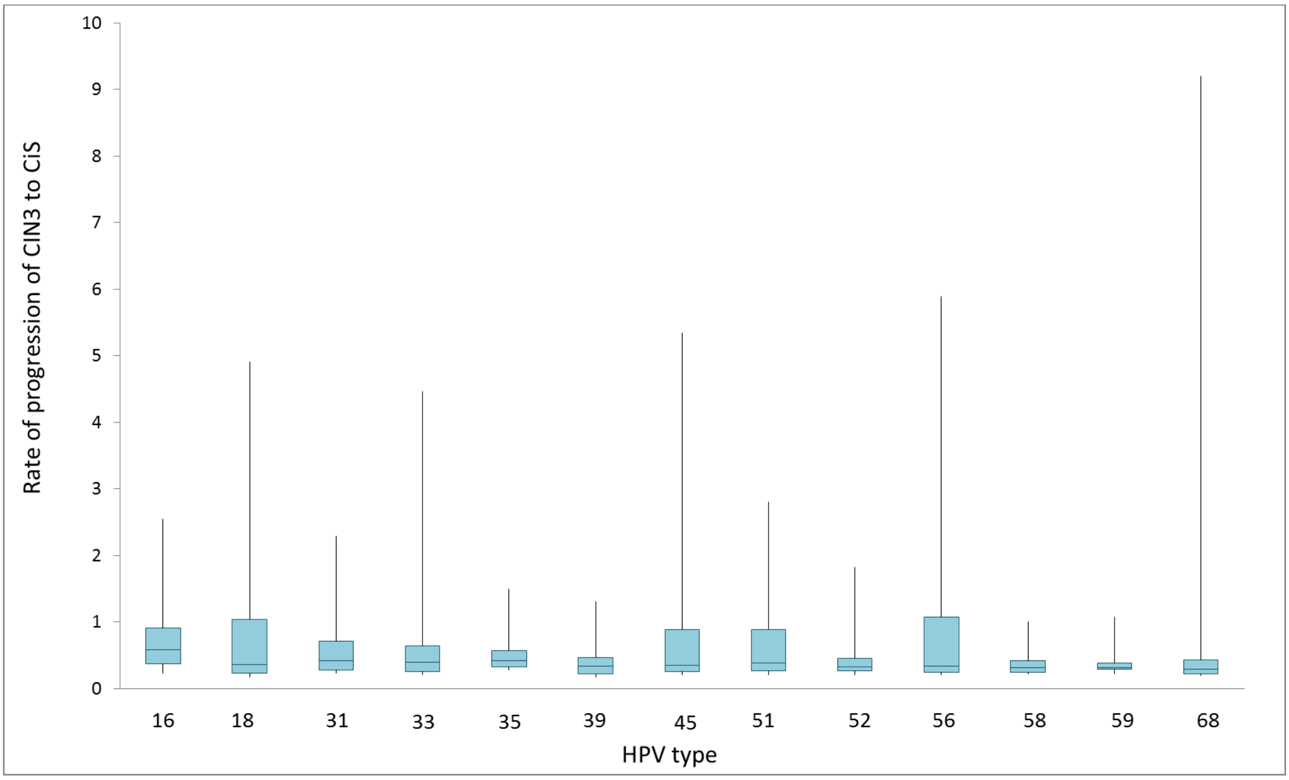

Figure S26 Posterior distributions of the rate of progression of CIN3-type lesions to Carcinoma *in situ* for each of the 13 high-risk HPV types modelled. Box plots show the median value and interquartile range (25th and 75th percentiles), whiskers show the 5th and 95th percentiles. The prior distribution was for duration before progression and was Uniform[0,5] years.

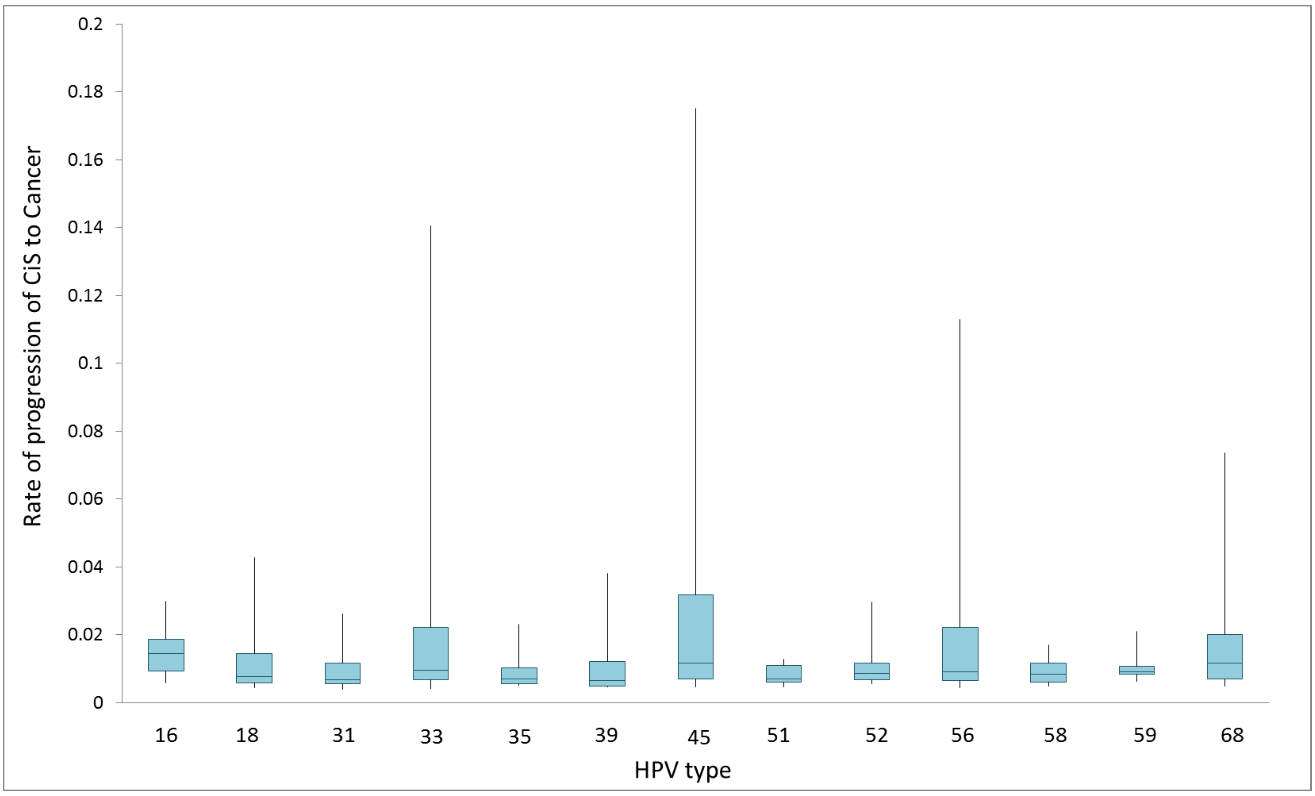

Figure S27 Posterior distributions of the rate of progression of Carcinoma *in situ* to cancer (squamous cell carcinoma or adenocarcinoma) for each of the 13 high-risk HPV types modelled. Box plots show the median value and interquartile range (25th and 75th percentiles), whiskers show the 5th and 95th percentiles. The prior distribution was for duration before progression and was Uniform[0, 200] years.

Table S9 Summary of model parameters that were not varied as part of the model-fitting process

Parameter		Average value	Standard Deviation	Further detail (section)	Source
**Demographics & Behaviour**					
Population size		100,000			
Proportion female		50%			
Proportion of ethnicity	White	88.8%			ONS
	Black	3.4%			ONS
	Asian	7.8%			ONS
Start age		10			
End age		85			
Birth rate	Age of mother			S1.3	
	10-14	0			ONS
	15-19	0.0199			ONS
	20-24	0.0699			ONS
	25-29	0.1051			ONS
	30-34	0.1139			ONS
	35-39	0.0637			ONS
	40-44	0.0146			ONS
	45-69	0			ONS
	70-84	0			ONS
Death rate (males)	10-14	0.000106		S1.3	ONS
	15-19	0.000316			ONS
	20-24	0.000480			ONS
	25-29	0.000571			ONS
	30-34	0.000757			ONS
	35-39	0.00109			ONS
	40-44	0.00168			ONS
	45-69	0.00669			ONS
	70-84	0.0418			ONS
Death rate (females)	10-14	0.0000825		S1.3	ONS
	15-19	0.000148			ONS
	20-24	0.000205			ONS
	25-29	0.000267			ONS

	30-34	0.000404			ONS
	35-39	0.000640			ONS
	40-44	0.000998			ONS
	45-69	0.00450			ONS
	70-84	0.0305			ONS
Sexual debut (males)	White	15.79	1.64	S1.4	NATSAL-3
	Black	15.68	1.37	S1.4	NATSAL-3
	Asian	17.12	1.52	S1.4	NATSAL-3
Sexual debut (females)	White	15.78	1.39	S1.4	NATSAL-3
	Black	15.72	1.38	S1.4	NATSAL-3
	Asian	17.12	1.50	S1.4	NATSAL-3
Rates of partnership formation	Detailed in Table S3				NATSAL-3
Proportion of partnerships identified as **casual** (**low** sexual activity group)				S1.6	NATSAL-3
	16-18	0.312			
	19-21	0.335			
	22-24	0.273			
	25-29	0.349			
	30-34	0.317			
	35-39	0.274			
	40-49	0.322			
	50+	0.635			
Proportion of partnerships identified as **casual** (**high** sexual activity group)				S1.6	NATSAL-3
	16-18	0.678			
	19-21	0.688			
	22-24	0.568			
	25-29	0.600			
	30-34	0.557			
	35-39	0.447			
	40-49	0.564			
	50+	0.641			
Proportion of partnerships identified as **steady** (**low** sexual activity group)				S1.6	NATSAL-3

	16-18	0.349			
	19-21	0.307			
	22-24	0.274			
	25-29	0.280			
	30-34	0.146			
	35-39	0.258			
	40-49	0.310			
	50+	0.193			
Proportion of partnerships identified as **steady** (**high** sexual activity group)				S1.6	NATSAL-3
	16-18	0.263			
	19-21	0.242			
	22-24	0.280			
	25-29	0.270			
	30-34	0.185			
	35-39	0.354			
	40-49	0.250			
	50+	0.284			
Duration of partnership by age and type	Detailed in Table S4				NATSAL-3
Mixing matrices	Detailed in Figs. S5 and S5a				NATSAL-3
Frequency of sex (acts per month)	Truncated normal	1.98	3.5	S1.9	NATSAL-3
**Natural history**					
Percentage of cervical cancer caused by type which is defined as Squamous Cell Carcinoma (so-called 'branching fraction')					Howell-Jones et al.
	16	86%			
	18	57%			
	31	87%			
	33	82%			
	35	100%			
	39	89%			
	45	88%			
	51	100%			

	52	86%			
	56	100%			
	58	100%			
	59	86%			
	68	80%			
Attributability of non-cervical cancer to HPV by type	Detailed in Table S5				ONS/PHE
Progression and clearance rates to cervical lesions	Posterior ranges detailed in Figures S20-S27				
Progression to non-cervical cancers	Posterior ranges detailed in Table S8				
**Cervical screening**					
Sensitivity and specificity by stage	Detailed in Table S6				
Cervical screening uptake (ever screened)	White	96.9%		S3.1	NATSAL-3
	Black	93.7%		S3.1	NATSAL-3
	Asian	87.0%		S3.1	NATSAL-3
Cervical screening uptake (recently screened)	White	80.5%		S3.1	NATSAL-3
	Black	79.5%		S3.1	NATSAL-3
	Asian	81.9%		S3.1	NATSAL-3
**Vaccine**					
Uptake of >=1 vaccine dose (routine cohort)	White	92.9%		S3.1	Primary Care Trusts (PHE)
	Black	88.2%		S3.1	Primary Care Trusts (PHE)
	Asian	88.4%		S3.1	Primary Care Trusts (PHE)
Uptake of >=1 vaccine dose (catch-up cohort: 14-15 year olds)	White	94.8%		S3.1	Primary Care Trusts (PHE)
	Black	91.7%		S3.1	Primary Care Trusts (PHE)
	Asian	94.1%		S3.1	Primary Care Trusts (PHE)
Uptake of >=1 vaccine dose (catch-up cohort:16-18 year olds)	White	78.6%		S3.1	Primary Care Trusts (PHE)
	Black	72.9%		S3.1	Primary Care Trusts (PHE)
	Asian	40.6%		S3.1	Primary Care Trusts (PHE)

Uptake of full-course of vaccination (routine cohort)	White	88.9%		S3.1	Primary Care Trusts (PHE)
	Black	79.2%		S3.1	Primary Care Trusts (PHE)
	Asian	83.1%		S3.1	Primary Care Trusts (PHE)
Uptake of full-course of vaccination (catch-up cohort: 14-15 year olds)	White	88.6%		S3.1	Primary Care Trusts (PHE)
	Black	70.5%		S3.1	Primary Care Trusts (PHE)
	Asian	86.3%		S3.1	Primary Care Trusts (PHE)
Uptake of full-course of vaccination (catch-up cohort: 16-18 year olds)	White	69.8%		S3.1	Primary Care Trusts (PHE)
	Black	26.3%		S3.1	Primary Care Trusts (PHE)
	Asian	36.3%		S3.1	Primary Care Trusts (PHE)

References

1. Bouvard V, Baan R, Straif K, Grosse Y, Secretan B, El Ghissassi F, et al. A review of human carcinogens--Part B: biological agents. The Lancet Oncology. 2009; **10**(4): 321-2.
2. Munoz N, Bosch FX, de Sanjose S, Herrero R, Castellsague X, Shah KV, et al. Epidemiologic classification of human papillomavirus types associated with cervical cancer. The New England journal of medicine. 2003; **348**(6): 518-27.
3. Brown DR, Schroeder JM, Bryan JT, Stoler MH, Fife KH. Detection of multiple human papillomavirus types in Condylomata acuminata lesions from otherwise healthy and immunosuppressed patients. Journal of clinical microbiology. 1999; **37**(10): 3316-22.
4. Greer CE, Wheeler CM, Ladner MB, Beutner K, Coyne MY, Liang H, et al. Human papillomavirus (HPV) type distribution and serological response to HPV type 6 virus-like particles in patients with genital warts. Journal of clinical microbiology. 1995; **33**(8): 2058-63.
5. Field N, Mercer CH, Sonnenberg P, Tanton C, Clifton S, Mitchell KR, et al. Associations between health and sexual lifestyles in Britain: findings from the third National Survey of Sexual Attitudes and Lifestyles (Natsal-3). Lancet. 2013; **382**(9907): 1830-44.
6. Mercer CH, Tanton C, Prah P, Erens B, Sonnenberg P, Clifton S, et al. Changes in sexual attitudes and lifestyles in Britain through the life course and over time: findings from the National Surveys of Sexual Attitudes and Lifestyles (Natsal). Lancet. 2013; **382**(9907): 1781-94.
7. Sonnenberg P, Clifton S, Beddows S, Field N, Soldan K, Tanton C, et al. Prevalence, risk factors, and uptake of interventions for sexually transmitted infections in Britain: findings from the National Surveys of Sexual Attitudes and Lifestyles (Natsal). Lancet. 2013; **382**(9907): 1795-806.
8. Tanton C, Soldan K, Beddows S, Mercer CH, Waller J, Field N, et al. High-Risk Human Papillomavirus (HPV) Infection and Cervical Cancer Prevention in Britain: Evidence of Differential Uptake of Interventions from a Probability Survey. Cancer epidemiology, biomarkers & prevention : a publication of the American Association for Cancer Research, cosponsored by the American Society of Preventive Oncology. 2015; **24**(5): 842-53.
9. Howell-Jones R, de Silva N, Akpan M, Oakeshott P, Carder C, Coupland L, et al. Prevalence of human papillomavirus (HPV) infections in sexually active adolescents and young women in England, prior to widespread HPV immunisation. Vaccine. 2012; **30**(26): 3867-75.
10. Mesher D, Soldan K, Howell-Jones R, Panwar K, Manyenga P, Jit M, et al. Reduction in HPV 16/18 prevalence in sexually active young women following the introduction of HPV immunisation in England. Vaccine. 2013; **32**(1): 26-32.
11. Howell-Jones R, Bailey A, Beddows S, Sargent A, de Silva N, Wilson G, et al. Multi-site study of HPV type-specific prevalence in women with cervical cancer, intraepithelial neoplasia and normal cytology, in England. British journal of cancer. 2010; **103**(2): 209-16.
12. Kitchener HC, Almonte M, Gilham C, Dowie R, Stoykova B, Sargent A, et al. ARTISTIC: a randomised trial of human papillomavirus (HPV) testing in primary cervical screening. Health Technol Assess. 2009; **13**(51): 1-150, iii-iv.
13. Kitchener HC, Almonte M, Wheeler P, Desai M, Gilham C, Bailey A, et al. HPV testing in routine cervical screening: cross sectional data from the ARTISTIC trial. British journal of cancer. 2006; **95**(1): 56-61.
14. Sargent A, Bailey A, Almonte M, Turner A, Thomson C, Peto J, et al. Prevalence of type-specific HPV infection by age and grade of cervical cytology: data from the ARTISTIC trial. British journal of cancer. 2008; **98**(10): 1704-9.
15. Parkin DM, Bray F. The burden of HPV-related cancers. Vaccine. 2006; **24**: S11-S25.

16. Daling JR, Madeleine MM, Schwartz SM, Shera KA, Carter JJ, McKnight B, et al. A population-based study of squamous cell vaginal cancer: HPV and cofactors. Gynecologic oncology. 2002; **84**(2): 263-70.
17. Kreimer AR, Clifford GM, Boyle P, Franceschi S. Human papillomavirus types in head and neck squamous cell carcinomas worldwide: a systematic review. Cancer Epidemiology and Prevention Biomarkers. 2005; **14**(2): 467-75.
18. Torrente MC, Rodrigo JP, Haigentz M, Dikkers FG, Rinaldo A, Takes RP, et al. Human papillomavirus infections in laryngeal cancer. Head & neck. 2011; **33**(4): 581-6.
19. Burington B, Hughes JP, Whittington WL, Stoner B, Garnett G, Aral SO, et al. Estimating duration in partnership studies: issues, methods and examples. Sexually transmitted infections. 2010; **86**(2): 84-9.
20. Copas AJ, Mercer CH, Farewell VT, Nanchahal K, Johnson AM. Recent heterosexual partnerships and patterns of condom use: a weighted analysis. Epidemiology. 2009: 44-51.
21. Iwasawa A, Nieminen P, Lehtinen M, Paavonen J. Human papillomavirus DNA in uterine cervix squamous cell carcinoma and adenocarcinoma detected by polymerase chain reaction. Cancer. 1996; **77**(11): 2275-9.
22. Rubin MA, Kleter B, Zhou M, Ayala G, Cubilla AL, Quint WG, et al. Detection and typing of human papillomavirus DNA in penile carcinoma: evidence for multiple independent pathways of penile carcinogenesis. The American journal of pathology. 2001; **159**(4): 1211-8.
23. Cuzick J, Szarewski A, Cubie H, Hulman G, Kitchener H, Luesley D, et al. Management of women who test positive for high-risk types of human papillomavirus: the HART study. Lancet. 2003; **362**(9399): 1871-6.
24. Mesher D, Szarewski A, Cadman L, Cubie H, Kitchener H, Luesley D, et al. Long-term follow-up of cervical disease in women screened by cytology and HPV testing: results from the HART study. British journal of cancer. 2010; **102**(9): 1405-10.
25. Karnon J, Peters J, Platt J, Chilcott J, McGoogan E, Brewer N. Liquid-based cytology in cervical screening: an updated rapid and systematic review and economic analysis. Health Technol Assess. 2004; **8**(20): iii, 1-78.
26. Beer H, Hibbitts S, Brophy S, Rahman MA, Waller J, Paranjothy S. Does the HPV vaccination programme have implications for cervical screening programmes in the UK? Vaccine. 2014; **32**(16): 1828-33.
27. Palmer TJ, McFadden M, Pollock KGJ, Kavanagh K, Cuschieri K, Cruickshank M, et al. HPV immunisation and increased uptake of cervical screening in Scottish women; observational study of routinely collected national data. British journal of cancer. 2016; **114**(5): 576-81.
28. Bowyer HL, Forster AS, Marlow LA, Waller J. Predicting human papillomavirus vaccination behaviour among adolescent girls in England: results from a prospective survey. The journal of family planning and reproductive health care / Faculty of Family Planning & Reproductive Health Care, Royal College of Obstetricians & Gynaecologists. 2014; **40**(1): 14-22.
29. Wheeler CM. Natural history of human papillomavirus infections, cytologic and histologic abnormalities, and cancer. Obstetrics and gynecology clinics of North America. 2008; **35**(4): 519-36; vii.
30. Malagon T, Drolet M, Boily MC, Franco EL, Jit M, Brisson J, et al. Cross-protective efficacy of two human papillomavirus vaccines: a systematic review and meta-analysis. The Lancet Infectious diseases. 2012; **12**(10): 781-9.
